# Supplementary material for: Health economic evaluations of preventative care for perinatal anxiety and associated disorders: a rapid review
Source: BMJ Open. 2024 Feb 27;14(2):e068941. doi: 10.1136/bmjopen-2022-068941 (PMC10900391; doi:10.1136/bmjopen-2022-068941)
Supplement: Supplementary data [file bmjopen-2022-068941supp001.pdf]

Search strategy

Below is an example of a search strategy for the Medline database.

ID	Search terms
1	exp Pregnancy/
2	(pregnan* or childbearing).ti,ab,kw.
3	(postpartum or post-partum or postnatal or postnatal or perinatal or peri-natal or prenatal or pre-natal or antenatal or ante-natal or matern*).ti,ab,kw.
4	perinatal anxiety.ti,ab,kw. or exp Perinatal anxiety/
5	exp Depression/
6	depress$.ti,ab,kw.
7	5 or 6
8	(Infant or baby or child).ti,ab,kw
9	(care* or treatment).tiab.kw
10	NHS.ti,ab,kw
11	hospitali$ation*.ti,ab,kw
12	exp Resource allocation/
13	economic evaluation$.ti,ab,kw.
14	(cost* or economic* or pharmacoeconomic*).ti.
15	13 or 14
16	exp "costs and cost analysis"/ or exp Health Care Costs/
17	exp Cost-Benefit Analysis/
18	(cost* adj2 (effective* or utility* or benefit* or consequence* or minimi*)).ti,ab,kw.
19	16 or 17 or 18
20	quality-adjusted life year$.ti,ab,kw. or exp Quality-Adjusted Life Years/
21	Or 7 and 15 and 19

Pisavadia K, *et al. BMJ Open* 2024; 14:e068941. doi: 10.1136/bmjopen-2022-068941

Quality appraisal of health economic evaluation studies [24]

**Drummond et al checklist 2015**	**Petrou et al (2002)** [40]	**Petrou et al (2006)** [4]	**Ride et al (2016)** [32]	**Henderson et al (2019)** [38]
1. Was a well defined question posed in an answerable form?	Yes	Yes	Yes	Yes
2. Was a comprehensive description of the competing alternatives given?	n/a	n/a	Yes	Yes
3. Was the effectiveness of the programs or services established?	n/a	n/a	Yes	Yes
4. Were all the important and relevant costs and consequences for each alternative identified?	n/a	n/a	Yes	Yes
5. Were costs and consequences measured accurately in appropriate physical units?	Yes	Yes	Yes	Yes
6. Were costs and consequences valued credibly?	Yes	Yes	Yes	Yes
7. Were costs and consequences adjusted for differential timing	n/a	n/a	No	No
8. Was an incremental analysis of costs and consequences of alternatives performed?	n/a	n/a	No	Yes
9. Was allowance made for uncertainty in the estimates of costs and consequences?	Yes	Yes	Yes	Yes
10. Did the presentation and discussion of study results include all issues of concern to users?	Yes	Yes	Yes	Yes

Source of checklist: Drummond, M. F., Sculpher, M. J., Claxton, K., Stoddart, G. L., & Torrance G W. (2015). *Methods for the economic evaluation of health care programmes* (4th ed.). Oxford: Oxford University Press.

Quality appraisal of health economic modelling studies with CHARMS Checklist [25]

Domain	Key items	Counts et al (2022) - [34]	Franta et al (2022) - (Franta et al., 2022)	Ride (2018) - [45]	Wilkinson et al (2017) - [5]	Bauer et al (2015) (Bauer et al, 2015)	Stevenson et al, (2010) [42]
**SOURCE OF DATA**	Source of data (e.g., cohort, case-control, randomized trial participants.	p.3	p.2	p.575	p.3	p.52	p.581
**PARTICIPANTS**	Participant eligibility and recruitment method (e.g., consecutive participants, location, [illegible]	p.3	p.2	p.575	p.3	p.52	p.581
	Participant description	p.3	p.2	p.575	p.3	p.52	p.581
	Details of treatments received, if	p.5	p.2	p.575	p.3	p.52	N/A
	Study dates	p.4	p.2	p.575	p.3	p.52	p.581
**OUTCOME(S) TO BE PREDICTED**	Definition and method for measurement of outcome	p.4	p.2	p.574	p.4	p.53	p.581-582
	Was the same outcome definition (and method for measurement) used in all	Yes p.5	p.2	p.574	p.4	p.53	p.581-582
	Type of outcome (e.g., single or combined [illegible]	p.3	p.5	p.574	p.4	p.53	p.581
	Was the outcome assessed without knowledge of	No	No	No	No	No	p.581
	Were candidate predictors part of the outcome	No	No	No	No	No	p.581

Pisavadia K, *et al. BMJ Open* 2024; 14:e068941. doi: 10.1136/bmjopen-2022-068941

	Time of outcome occurrence or summary of duration of follow-up	p.5	p.5	p.578	p.4	p.52	p.581
**CANDIDATE PREDICTORS (OR INDEX TESTS)**	Number and type of predictors (e.g., demographics, patient history, physical examination,	p.5	p.5	p.577	p.6	p.55	p.582
	Definition and method for measurement of candidate predictors	p.5	p.5	p.575	p.6	p.55	p.580-582
	Timing of predictor measurement (e.g., at patient presentation, at diagnosis,	p.5	p.5	p.577	p.6	p.55	p.581
	Were predictors assessed blinded for outcome, and for each other	No	No	No	No	No	p.582
	Handling of predictors in the modelling (e.g., continuous, linear, non-linear transformatio	Unclear	Unclear	Unclear	Unclear	p.52	p.582
**SAMPLE SIZE**	Number of participants and number of outcomes/ev	p.3	p.2	p.575	P.3	p.55	p.582
	Number of outcomes/events in relation to the number of candidate predictors (Events Per Variable)	p.5	p.3	p.577	p.20	p.57	p.582
**MISSING DATA**	Number of participants with any missing value (includ	p.4	Unclear	Unclear	Unclear	Unclear	Unclear

	Number of participants with missing data for each predictor	Unclear	Unclear	Unclea r	Unclear	Unclea r	Unclear
	Handling of missing data (e.g., complete-case analysis, imputation, or	Unclear	Unclear	Unclea r	Unclear	Unclea r	Unclear
**MODEL DEVELOPMEN T**	Modelling method (e.g., logistic, survival, neural network, or	Simulate d cohort model	Simulat ed cohort model	Decisi on analyti c model	Simulat ed cohort model	Decisi on analyti c model	Mathemati cal model
	Modelling assumptions satisfied	See Appendix 1 in the supplem ent	p.5	p.577	p.4	p.53	p.580
	Method for selection of predictors **for inclusion** in multivariable modelling (e.g., all candidate predictors, pre-selection based on unadjusted association with the	Unclear	Unclear	p.577	p.4	p.53	p.581
	Method for selection of predictors **during multivariable modelling** (e.g., full model approach, backward or forward selection) and criteria used (e.g., p-value, Akaike Information Criterion)	Unclear	Unclear	Unclea r	Unclear	p.53	Unclear

	Shrinkage of predictor weights or regression coefficients (e.g., no shrinkage, [illegible]	Unclear	Unclear	Unclear	Unclear	Unclear	Unclear
**MODEL PERFORMANCE**	Calibration (calibration plot, calibration slope, Hosmer-Lemeshow test) and Discrimination (C-statistic, D-statistic, log-rank)	p.5	Unclear	Unclear	Unclear	Unclear	Unclear
	Classification measures (e.g., sensitivity, specificity, predictive values, net reclassification improvement) and whether a-priori cut	See e-appendix 3	p.6	p.577	p.6	No	p.581
**MODEL EVALUATION**	Method used for testing model performance: development dataset only (random split of data, resampling methods e.g. bootstrap or cross-validation, none) or separate external validation (e.g. temporal, geographical	See e-appendix 3	Unclear	Unclear	p.6	No	Unclear

	In case of poor validation, whether model was adjusted or updated (e.g., intercept recalibrated, predictor effects	Unclear	Unclear	Unclea r	Unclear	No	Unclear
**RESULTS**	Final and other multivariable models (e.g., basic, extended, simplified) presented, including predictor weights or regression coefficients, intercept, baseline survival, model performance measures (with	Unclear	Unclear	Unclea r	Unclear	No	No
	Any alternative presentation of the final prediction models, e.g., sum score, nomogram, score chart, predictions for specific risk subgroups	No	No	p.578	p.23	No	No
	Comparison of the distribution of predictors (including missing data) for development and validation	No	No	No	No	No	No

**INTERPRETATION AND DISCUSSION**	Interpretation of presented models (confirmatory, i.e., model useful for practice versus exploratory, i.e., more [illegible]	p.7	p.5	p.577	p.6	p.56	p.583
	Comparison with other studies, discussion of generalizability, strengths and limitations.	p.7	p.5	p.577	p.6	p.58	p.583

JBI critical appraisal checklist for Systematic Reviews and Research Syntheses [26]

Citation	Q1. Is the review question clearly and explicitly stated?	Q2. Were the inclusion criteria appropriate for the review question?	Q3. Was the search strategy appropriate?	Q4. Were the sources and resources used to search for studies adequate?	Q5. Were the criteria for appraising studies appropriate?	Q6. Was critical appraisal conducted by two or more reviewers independently?	Q7. Were there methods to minimize errors in data extraction?	Q8. Were the methods used to combine studies appropriate?	Q9. Was the likelihood of publication bias assessed?	Q10. Were recommendations for policy and/or practice supported by the reported data?	Q11. Were the specific directives for new research appropriate?
(Camacho & Shields, 2018)	Yes	Yes	Yes	Yes	Yes	Unclear	Yes	Yes	Yes	Yes	Yes
[37]	Yes	Yes	Yes	Yes	Yes	Unclear	Yes	Unclear	Yes	Yes	Yes
(Moran et al., 2020)	Yes	Yes	Yes	Yes	Yes	Yes	Yes	Yes	Yes	Unclear	Yes
(Morrell et al., 2016)	Yes	Yes	Yes	Yes	Yes	Yes	Yes	Yes	Yes	Yes	Yes

JBI Critical appraisal checklist for randomized controlled trials [27]

Citation	Q1. Was true randomization used for assignment of participants to treatment groups?	Q2. Was allocation to treatment groups concealed?	Q3. Were treatment groups similar at the baseline?	Q4. Were participants blind to treatment assignment?	Q5. Were those delivering treatment blind to treatment	Q6. Were outcomes assessors blind to treatment	Q7. Were treatment groups treated identically other than the	Q8. Was follow up complete and if not, were differences	Q9. Were participants analyzed in the groups to which they were	Q10. Were outcomes measured in the same way for	Q11. Were outcomes measured in a reliable way?	Q12. Was appropriate statistical analysis used?	Q13. Was the trial design appropriate, and any deviations from the standard

					assignment?	assignment?	intervention of interest?	between groups in terms of their follow up adequately described and analyzed?	randomized?	treatment groups?			RCT design (individual randomization, parallel groups) accounted for in the conduct and analysis of the trial?
[29]	Yes	Yes	Yes	Unclear	Yes	Unclear	Yes	Yes	No	Yes	Unclear	Yes	N/A
[44]	Yes	Yes	Yes	Yes	Yes	Unclear	Yes	Yes	Yes	Yes	Yes	Yes	Yes

JBI Critical Appraisal Checklist for Cohort Studies [28]

**Citation**	Q1. Were the two groups similar and recruited from the same population?	Q2. Were the exposures measured similarly to assign people to both exposed and unexposed groups?	Q3. Was the exposure measured in a valid and reliable way?	Q4. Were confounding factors identified?	Q5. Were strategies to deal with confounding factors stated?	Q6. Were the groups/ participants free of the outcome at the start of the study (or at the moment of exposure)?	Q7. Were the outcomes measured in a valid and reliable way?	Q8. Was the follow up time reported and sufficient to be long enough for outcomes to occur?	Q9. Was follow up complete, and if not, were the reasons to loss to follow up described and explored?	Q10. Were strategies to address incomplete follow up utilized?	Q11. Was appropriate statistical analysis used?
(Moore Simas et al., 2020)	Yes	Yes	Yes	No	No	Yes	Yes	Yes	Unclear	N/A	Yes

JBI Critical Appraisal Checklist for Cross-sectional studies [28]

Citation	Q1. Were the criteria for inclusion in the sample clearly defined?	Q2. Were the study subjects and the setting described in detail?	Q3. Was the exposure measured in a valid and reliable way?	Q4. Were objective, standard criteria used for measurement of the condition?	Q5. Were confounding factors identified?	Q6. Were strategies to deal with confounding factors stated?	Q7. Were the outcomes measured in a valid and reliable way?	Q8. Was appropriate statistical analysis used?
Dagher et al., 2012	Yes	Yes	Yes	Yes	No	No	Yes	Yes
Chojenta et al., 2019	Yes	Yes	Yes	Yes	Yes	Yes	Yes	Unclear
Ammerman et al., 2016	Yes	Yes	Yes	Yes	Yes	Yes	Yes	Yes
Roberts et al., 2001	Yes	Yes	Yes	Yes	No	N/A	Yes	Yes

Abbreviations

Abbreviation	Full	Aspect
ANOVA	Analysis of Variance	Analysis
ANRQ-R	Antenatal Risk Questionnaire	Tool
CATi	Computer Assisted Telephone Interviews	Research
CBA	Cognitive Behavioural Approach	Intervention
CBT	Cognitive Behavioural Therapy	Intervention
CEA	Cost Effectiveness Analysis	Analysis
CIDI	Composite International Diagnostic Interview	Research
CUA	Cost Utility Analysis	Analysis
DASS21	Depression, Anxiety and Stress Scale	Tool
DCS	Depression Care Specialist	Staff
DFD	Disease Free Day	Research
DSM-IV	Diagnostic and Statistical Manual for Mental Disorders 4th Edition	Source
eMBI	electronic Mindfulness-based Intervention	Intervention
EPDS	Edinburgh Postnatal Depression Scale	Tool
ePRO	electronic Patient Reported Outcomes	Research
EQ-5D-3L	EuroQol 5 Dimension 3 Level	Tool
GP	General Practitioner	Staff
gCBT	Group cognitive behavioural therapy	Intervention
HRU	Healthcare resource utilization	Analysis
HV	Health Visitor	Staff
ICD	International Classification of Diseases	Source
ICER	Incremental Cost-Effectiveness Ratio	Analysis
IG	Intervention Group	Research
IPT	Interpersonal psychotherapy	Intervention
ITT	Intention to Treat	Research
LGA	Local Government Area	Organisation
MBS	Medical Benefits Schedule	Source
MCH	Maternal and Child Health	Setting
MFAS	Maternal-Fetal Attachment Scale	Tool
MOMcare		*Study name*
MINI	Mini-International Neuropsychiatric Interview	Tool
NHS	National Health Service	Setting
OOP	Out of Pocket	Research
PAD	perinatal anxiety and/or depression	Diagnosis
PBS	Pharmaceutical Benefits Scheme	Source
PCA	Personalised Care Approach	Intervention
PHQ-9	Patient Health Questionnaire	Tool
PND	Postnatal depression	Diagnosis
PND	Post-partum depression	Diagnosis
PoNDER trial	POstNatal Depression Economic evaluation and Randomised trial	*Study name*
PRAQ-R	Pregnancy-Related Anxiety Questionnaire	Tool
PTSD	Post-Traumatic Stress Disorder	Diagnosis
QALY	Quality Adjusted Life Year	Analysis

RCT	Randomised controlled trial	Research
SCL-20	Hopkins Symptom Checklist-20	Tool
SF36	Short-Form 36	Tool
SIDS	Sudden infant death syndrome	Diagnosis
SPARCS	*Sleep, Parenting and Relationships in a Community Setting*	*Study name*
STAI	State-Trait Anxiety Questionnaire	Tool
TAU	Treatment as Usual	Research
TENS	Transcutaneous Electrical Nerve Stimulation	Intervention
WHO	World Health Organisation	Organisation
WWWT	What Were We Thinking	Tool

Pisavadia K, *et al. BMJ Open* 2024; 14:e068941. doi: 10.1136/bmjopen-2022-068941
