# Supplementary material for: Health economic evaluations of preventative care for perinatal anxiety and associated disorders: a rapid review
Source: BMJ Open. 2024 Feb 27;14(2):e068941. doi: 10.1136/bmjopen-2022-068941 (PMC10900391; doi:10.1136/bmjopen-2022-068941)
Supplement: Supplementary data [file bmjopen-2022-068941supp002.pdf]

Data extraction table for studies including perinatal anxiety

Citation (Country)	Study Details	Participants and setting	Key findings	Observations
Ride et al (2016)<br><br>(Ride et al., 2016)<br><br>**Australia**	**Study Design:** Economic evaluation, including cost-effectiveness and cost-utility analyses, conducted alongside a cluster-randomised trial<br><br>**Type of intervention [*exposure*]:** What Were We Thinking (WWWT) - a psychoeducational intervention targeted at the partner relationship, management of infant behaviour and parental fatigue.<br><br>**Data collection methods:** Data were collected from participants via computer-assisted telephone interview at baseline (6 weeks postpartum) and follow-up (26 weeks postpartum).	**Sample size:** 359<br><br>**Participants:** English-speaking first-time mothers who had recently given birth and attended participating Maternal and Child Health Centres (MCHCs)<br><br>**Setting:** 48 Maternal and Child Health Centres in Victoria, Australia.<br><br>**Dates of data collection:** Baseline interviews took place between May 2013 and April 2014, and follow-up interviews between September 2013 and August 2014.	**Primary Findings:**<br>The intervention was estimated to cost $A118.16 per participant. The analysis showed no statistically significant difference between the intervention and control groups in costs or outcomes. The incremental cost-effectiveness ratios were $A36 451 per QALY gained and $A152 per percentage point reduction in 30-day prevalence of depression, anxiety, and adjustment disorders. The estimate lies under the unofficial cost-effectiveness threshold of $A55 000 per QALY; however, there was considerable uncertainty surrounding the results, with a 55% probability that WWWT would be considered cost-effective at that threshold.<br>**Additional Findings:**<br>The results suggest that, although WWWT shows promise as a preventive intervention for postnatal maternal mental health problems, further research is required to reduce the uncertainty over its cost-effectiveness as there were no statistically significant differences in costs or outcomes.	Ride et al (2016) investigated the cost-effectiveness of the What Were We Thinking (WWWT) intervention, for the prevention of postnatal maternal mental health problems. The intervention was estimated to cost $A118.16 per participant. The analysis showed no statistically significant difference between the intervention and control groups in costs or outcomes. The incremental cost-effectiveness ratios were $A36 451 per QALY gained and $A152 per percentage point reduction in 30-day prevalence of depression, anxiety, and adjustment disorders. The estimate lies under the unofficial cost-effectiveness threshold of $A55 000 per QALY; however, there was considerable uncertainty surrounding the results, with a 55% probability that WWWT would be considered cost-effective at that threshold

Data extraction table for studies including maternal depression

Citation (Country)	Study Details	Participants and setting	Key findings	Observations
Ammerman et al (2016)<br><br>(Ammerman et al., 2016)<br><br>USA	**Study design:** Cross-sectional<br><br>**Data collection methods:** MEPS database, a subset of the National Health Interview Survey (NHIS) that includes information on health care utilisation and expenditures for the civilian, non-institutionalised population in the USA.	**Sample size:** 20,531<br><br>**Participants:** 2,310 high-risk mothers with depression and 18,221 high-risk mothers without depression<br><br>**Setting:** USA healthcare setting<br><br>**Dates of data collection:** 1996 to 2011	**Primary findings:**<br>Depressed mothers were more likely to incur insurer (0.88 vs. 0.80) and out of pocket expenses (0.86 vs. 0.77) and to have higher insurer expenses ($4916 vs. $3521) and out of pocket expenses ($786 vs. $522) (in 2015).<br>**Additional findings:**<br>A higher proportion of the depressed sample was Caucasian which were in relatively worse health than women from other ethnic groups. The depressed sample was more likely to have public insurance, to be English-speaking and to have a usual health care provider.	The cross-sectional study from Ammerman et al (2016) the USA conducted between 2006 and 2011 investigated the out-of-pocket expenses and insurer expenses of depressed vs non-depressed mothers. Depressed mothers were more likely to incur insurer and out of pocket expenses and to have higher insurer expenses ($4916 vs. $3521) and out of pocket expenses ($786 vs. $522) (in 2015).
Bauer et al (2015)<br><br>(Bauer et al., 2015)<br><br>UK	**Study Design:** The economic analysis takes a life-course perspective from the viewpoints of the public sector, individual and society. The study analysed the effects of perinatal depression on child development outcomes of children at ages 11 and 16 years from the community-based South London Child Development Study. Economic consequences were attached to those outcomes through simple decision-analytic techniques, building on evidence from studies of epidemiology, health-related quality of life, public sector costs and employment.	**Sample size:** 120<br><br>**Participants:** Mothers and children<br><br>**Setting:** Two antenatal clinics in the UK<br><br>**Dates of data collection:** January to December 1986	**Primary Findings:**<br>Additional risks that children exposed to perinatal depression develop emotional, behavioural, or cognitive problems ranged from 5% to 21%. In addition, there was a high risk (24%) that children would have special educational needs.<br><br>For each child exposed to perinatal depression, public sector costs exceeded £3,030, costs due to reduced earnings were £1,400 and health-related quality of life loss was valued at £3,760.	The study examined some of the outcomes and long-term economic implications experienced by offspring who have been exposed to perinatal depression.
Counts et al (2022)<br><br>(Counts et al., 2022)<br><br>USA	**Study Design:** Modelling study. A decision analytic model used a simulated cohort of 1,000 Medicaid-enrolled pregnant individuals. Health care costs for individuals receiving postpartum depression preventive intervention or not, over 1 or 5 years postpartum, in a variety of scenarios, including varying rates of Medicaid churn (i.e., transitions to a new Medicaid managed care plan, commercial insurance plan, or loss of coverage) were	**Sample size:** 1,000<br><br>**Participants:** simulated cohort of 1,000 Medicaid enrolled pregnant individuals<br><br>**Setting:** USA healthcare system.	**Primary Findings:**<br>The main outcome was the amount of clinician incentive shared in a Value-based payment (VBP) model from providing preventive interventions. The likelihood of the health care payer realising a positive return on investment if it shared 50% of 5-year	This economic modelling study found that providing preventive interventions for PND resulted in an estimated 5-year saving of £602□

	estimated for the period 2020 to 2025. The model was developed between March 5 2021 and July 30 2021.<br><br>**Type of intervention [*exposure*]:** Individual counselling and group-based counselling.<br><br>**Data collection methods:** Simulation based on collected Medicaid data.	**Dates of data collection:** Model developed between March 5 2021 and July 30 2021.	expected savings with a clinician up front was also measured.<br><br>The simulated cohort was designed to be reflective of the demographics characteristics of pregnant individuals receiving Medicaid; however, no specific demographic features were simulated. Providing preventive interventions for postpartum depression resulted in an estimated 5-year savings of $734.12 (95% credible interval [CrI], $217.21-$1235.67) per person. Without health insurance churn, sharing 50% of 5-year expected savings could offer more than double the financial incentives for clinicians to prevent postpartum depression compared with traditional VBP ($367.06 [95% CrI, $108.61-$617.83] vs $177.74 [95% CrI, $52.66-$296.60], respectively), with a high likelihood of positive return for the health care payer (91%). As health insurance churn increased, clinician incentives from sharing estimated savings decreased (73% reduction with 50% annual churn).	
Dagher et al (2012)<br><br>(Dagher et al., 2012<br><br>USA	**Study design:** Cross-sectional<br><br>**Data collection methods:** Prices of service use and EPDS	**Sample size:** 638 women.<br><br>**Participants:** Women receiving maternal healthcare services, from hospital discharge to 11 weeks postpartum.<br><br>**Setting:** USA healthcare setting.<br><br>**Dates of data collection:** The year 2001.	**Primary findings:**<br>The total cost of all mental health counselling visits for the depressed group n =31 was $138 and the cost for the non-depressed group n= 607 was $13. This was a statistically significant difference (p < 0.001).<br><br>**Additional findings:** The total cost of emergency department visits for the postpartum women was $84 for the depressed group n = 31 and $13 for the non-depressed group n = 607. This was a statistically significant difference (p < 0.001).	The Dagher et al., (2012) cross-sectional study from the USA investigated expenditure from health care service from discharge until 11 weeks postpartum. There was a significant difference in healthcare expenditure between depressed and non-depressed women. The EPDS was used to measure depression. The total cost of all mental health counselling visits for the depressed group n =31 was $138 and the cost for the non-depressed group n= 607 was $13. This was a statistically significant difference (p < 0.001).

Franta et al (2022)<br><br>(Franta et al., 2022)<br><br>USA	**Study Design:**<br>Modelling study<br><br>**Type of intervention [*exposure*]:**<br>Comparison of outcomes in pregnant adolescents who received versus did not receive counselling interventions<br><br>**Data collection methods**: Decision-analytic model using TreeAge Pro software	**Sample size:** Theoretical cohort of 180,000 individuals<br><br>**Participants:** pregnant adolescents<br><br>**Setting:** Obstetric setting<br><br>**Dates of data collection:** 2018	**Primary Findings:**<br>• A strategy of referral to counselling interventions was cost effective in the theoretical cohort, with 8,935 fewer cases of perinatal depression, 1,606 fewer cases of chronic depression, 166 fewer preterm deliveries, 4 fewer neonatal deaths, 1 fewer case of cerebral palsy, 20 fewer cases of SIDS. In total, there were 21,976 additional QALYs and cost savings of $223,549,872, making it the dominant strategy (better outcomes with lower costs).<br>• Counselling interventions remained cost saving until the annual direct and indirect cost of chronic, severe depression was set below $30,000, at which point it became cost effective (baseline input: $182,309).<br>• It is cost effective to refer all pregnant adolescents for preventive counselling interventions.	Using a theoretical cohort, Franta et al. (2022) found that counselling was a cost-effective preventative measure, leading to fewer cases of perinatal and chronic depression
Grote et al (2017)<br><br>Grote et al., 2017)<br><br>**USA**	**Study Design:** RCT, cost-benefit study<br><br>**Type of intervention [*exposure*]:** 18 months **MOMCare** collaborative care depression intervention (choice of brief interpersonal psychotherapy or pharmacotherapy or both) with enhanced maternity support services (MSS-Plus).<br><br>**Data collection methods:** Blinded telephone assessments, including depression severity on SCL-20. Unit costs of MOMCare intervention actual salary rate + fringe benefits + 30% overheads	**Sample size:** 152<br><br>**Participants:** 152 pregnant women 12-32 wks. gestation with probable major depression or dysthymia (PTSD). Plus 12 excluded from analysis due to missing final data.<br><br>**Setting:** 10 county public health centres<br><br>**Dates of data collection:** Recruited Jan 2010 – July 2012. Study ended 2014	**Primary Findings:** when controlled for baseline depression severity, women with probable depression and PTSD in MOMCare had 68 more depression-free days over 18 months than those in MSS-Plus (p,.05). Additional $1,312. depression care cost per MOMCare participant with comorbid PTSD. Incremental net benefit of MOMCare was positive if a depression free days was valued at $\geq$ $20<br><br>**Additional Findings:**<br>Unit costs used 2013:<br>$80 per 45-50 min depression care specialist (DCS) visit<br>$31 per 20-30 min DCS phone call<br>(Both included time for outreach efforts and record keeping)<br>$247 fixed cost per patient for caseload supervision and info support<br>Other references to US-based data sources	In this RCT, cost-benefit study, a multicomponent collaborative care intervention for depression (MOMcare - a choice of brief interpersonal psychotherapy or pharmacotherapy or both) with enhanced maternity support services (MSS-Plus) in the public health system of Seattle, USA. The incremental benefit and cost and the net benefit for women with major depression and PTSD was estimated. When controlled for baseline depression severity, women with probable depression and PTSD in MOMCare had 68 more depression-free days over 18 months than those in MSS-Plus (p<.05). There was an additional £1,943[▪▪] depression care cost per MOMCare participant with comorbid PTSD. The incremental net benefit of MOMCare was positive if

				depression free days was valued below £18▪▪. For women with probable major depression and PTSD, MOMCare had a significant clinical benefit over MSS-Plus, with only a moderate increase in health services cost.[1]
Henderson et al (2019)<br><br>(Henderson et al., 2019)<br><br>**UK**	**Study Design:** PONDER Cluster RCT<br><br>**Type of intervention [*exposure*]:** GP practices assigned to usual health visitor (HV) care, HV trained to assess for PND plus offering either a CBA or a person-centred approach (PCA) weekly for 8 weeks<br><br>**Data collection methods:** Postal questionnaires: Baseline including EPDS and SF36 at 6 weeks, Postnatal questionnaires at 6, 12 and 18 months postnatal. Resource use logs were completed by HVs based on their and GP records	**Sample size: From** 101 GP practices, 4,084 participants consented, baseline data from 3,449 participants.<br><br>**Participants:** 2,241 lower risk women completed EPDS at 6 months – 767 control, 1,474 intervention. 1,459 women provided economic data.<br><br>**Setting:** GP practices<br><br>**Dates of data collection:** April 2003 for 3 years	**Primary Findings:** 99% probability of cost effectiveness at £20,000 at 6 months postnatal<br>Compared with controls, adjusted 6 months costs were £82 lower with the interventions<br><br>**Additional Findings:**<br>Little difference CBA to PCA – CBA marginally higher probability of being cost effective.	This study found that CBT had a marginally higher probability of being cost-effective than a person-centred approach.
Moore Simas et al (2020)<br><br>(Moore Simas et al., 2020)<br><br>USA	**Study Design:** Cohort study<br><br>**Type of intervention [*exposure*]:** PND.<br><br>**Data collection methods**: Administrative claims data from the IBM Watson Health MarketScan Databases	**Sample size:** 135,678<br><br>**Participants:** mother-child pairs with and without postpartum depression (PND) exposure<br><br>**Setting:** USA healthcare setting.<br><br>**Dates of data collection:** 2010 to 2016	**Primary Findings:**<br>• 33,314 mother-child pairs with PND exposure were propensity score matched to 102,364 mother-child pairs without PND exposure.<br>• During the 24-month follow-up period, HRU across most service categories was significantly higher among children in the PND exposure cohort than non-PND exposure cohort.<br>• Among outpatient services, the percentages of children with a physician specialist service (68% versus 64%), early-intervention screening (40% versus	This cohort study assessed healthcare resource utilisation (HRU) and costs in children of mothers with and without PND

1 ▪▪ Prices have been inflated and converted to GBP [53].

			37%), and an emergency room visit (48% versus 42%) were greater in children of mothers with PND (all *p* < .001).<br>• Furthermore, children of mothers with PND incurred 12% higher total healthcare costs in the first 24 months of life compared to children of mothers without PND ($24,572 versus $21,946; *p* < .001).<br>• After excluding mothers with preterm delivery, the proportion of children with ER visits, physician specialist services, and outpatient pharmacy claims was significantly higher in the PND exposure cohort than non-PND exposure cohort (all *p* < .001).<br>**Additional Findings:**<br>The results of this analysis suggest that HRU and costs over the first 24 months of life in children of mothers with PND exceeded that of children of mothers without evidence of PND.	
Petrou et al (2002)<br><br>(Petrou et al., 2002)<br><br>**UK**	**Study Design**: Economic evaluation in which unit costs were applied to resource-use data collected alongside a longitudinal study of women at high risk of developing PND. Unit costs were applied to estimates of health and social care resource use made by 206 women recruited from antenatal clinics and their infants. Net costs per mother-infant dyad over the first 18 months post-partum were estimated.<br><br>**Type of intervention [*exposure*]:** Preventative PND intervention.<br><br>**Data collection methods**: primiparous women attending antenatal clinics at 26–28 weeks of gestation were screened using a predictive index for PND. Women identified as being at high risk of developing PND were entered into an RCT of a preventive	**Sample size:** 206<br><br>**Participants:** Primiparous women at high risk of developing PND<br><br>**Setting:** antenatal clinics<br><br>**Dates of data collection:** May 1997 to April 1999	**Primary Findings:**<br>Mean mother-infant dyad costs were estimated at £2,419.00 for women with PND and £2026.90 for women without PND, a mean cost difference of £392.10 (P=0.17). The mean cost differences between women with and without PND reached statistical significance for community care services (P=0.01), but not for other categories of service. Economic costs were higher for women with extended experiences of the condition.	Aimed to estimate the economic costs of PND in a geographically defined cohort of women at high risk of developing the condition.

	intervention for PND delivered by trained health visitors. Economic data of women in the trial and in the observational study were pooled. An independent researcher assessed the mental state of all women at 8 weeks, 18 weeks, 12 months, and 18 months post-partum using the Structured Clinical Interview for DSM–III–R diagnoses (SCID–II).			
Petrou et al (2006)<br><br>(Petrou et al., 2006)<br><br>**UK**	**Study Design** A prospective economic evaluation was conducted alongside a pragmatic RCT<br><br>**Type of intervention [*exposure*]:** psychosocial and psychological interventions including counselling for the prevention of PND.<br><br>**Data collection methods:** Data on health and social care use by women and their infants up to 18 months postpartum were collected, using a combination of prospective diaries and face-to-face interviews	**Sample size:** 151 women<br><br>**Participants:** Women considered at high risk of developing PND were allocated randomly to the preventive intervention (*n* = 74) or to routine primary care (*n* = 77)<br><br>**Setting:** Health care setting.<br><br>**Dates of data collection:** c.2000	**Primary Findings:**<br>• Women in the preventive intervention group were depressed for an average of 2.21 months (9.57 weeks) during the study period, whereas women in the routine primary care group were depressed for an average of 2.70 months (11.71 weeks).<br>• The mean health and social care costs were estimated at £2,396.9 per mother–infant dyad in the preventive intervention group and £2,277.5 per mother–infant dyad in the routine primary care group, providing a mean cost difference of £119.5 (bootstrap 95 percent confidence interval [CI], −535.4, 784.9).<br>• At a willingness to pay threshold of £1,000 per month of PND avoided, the probability that the preventive intervention is cost-effective is .71 and the mean net benefit is £383.4 (bootstrap 95 percent CI, −£863.3–£1,581.5).<br><br>**Additional Findings:**<br>The preventive intervention is likely to be cost-effective even at relatively low willingness to pay thresholds for preventing 1 month of PND during the first 18 months postpartum. Given the negative impact of PND on later child development.	This cost-effectiveness analysis found that given the negative impact of PND on later child development, preventive interventions are likely to be cost-effective even at relatively low willingness to pay thresholds for preventing one month of PND during the first 18 months post-partum.
Roberts et al (2001)<br>[42]	**Study design:** Cross-sectional<br><br>**Data collection methods:** EPDS and the Health and Social Service Utilization Questionnaire (HSUQ)	**Sample size:** 1,250<br><br>**Participants:** mothers of infants.	**Primary findings:**<br>Costs were notably different for mothers with and without depression as determined by the EPDS (score of > 12). The total cost for health and social care $845 for mothers with	A cross-sectional study of 1250 mothers of infants in a Canadian setting used the EPDS to investigate the costs associated with perinatal depression. It was found that

**Canada**		**Setting:** Canadian healthcare setting<br><br>**Dates of data collection:** 1999	depression and their infant's vs $413 for those with lower scores. This was statistically significant difference at the (p < .01).<br>**Additional findings:**<br>Costs for social work visits were higher for mothers with depression and mothers with low incomes.<br>Total health and social care costs were double for mothers with family income below $20,000 ($788 v $399) and for mothers with clinical depression ($845 v $413). Nursing care costs were greater for mothers with high depression scores ($135 v $81).	costs were notably different for mothers with and without depression. The total cost for health and social care was $845 for mothers with depression and their infant's vs $413 for those with lower depression scores. This was statistically significant different at p < .01.
Stevenson et al (2010) (Stevenson et al., 2010)<br><br>UK	**Study Design:** cost-effectiveness analysis to assess group-CBT (gCBT) in comparison with routine primary care for women with PND in the UK.<br><br>**Type of intervention [*exposure*]:** Group-CBT<br><br>**Data collection methods:** SR	**Sample size:** 401<br><br>**Participants:** Data were analysed from 401 women with an EPDS score of 12 or greater at 6 weeks after childbirth, which had completed both the EPDS and the SF-6D questionnaire at both 6 weeks and 6 months<br><br>**Setting:** Postnatal healthcare setting in the UK<br><br>**Dates of data collection:** Pre-July 2009 (when PONDER study was published**).**	**Primary Findings:**<br>The mean cost per QALY from the stochastic analysis was estimated to be £36,062; however, there was considerable uncertainty around this value. The EVPI was estimated to be greater than £64 million; the key uncertainties were in the cost per woman of providing treatment and in the statistical relationship between changes in EPDS values and changes in SF-6D values. The expected value of perfect partial information for both of these parameters was more than £25 million.<br><br>**Additional Findings:**<br>The use of gCBT does not appear to be cost-effective; however, this decision is uncertain. The value of information analyses conducted indicates that further research to provide robust information on key parameters is needed and appears justified in cost-effective terms.	This economic evaluation found that gCBT does not appear to be cost-effective due to the lack of literature providing robust information. Only one study, an RCT, was deemed applicable to the decision problem.
Wilkinson et al (2017)<br><br>(Wilkinson et al., 2017)<br><br>**USA**	**Study Design:** Modelling study<br><br>**Type of intervention [*exposure*]:** N/A<br><br>**Data collection methods**: Hypothetical cohort	**Sample size:** 1,000<br><br>**Participants:** follows a hypothetical cohort of 1000 pregnant women experiencing one live birth over a 2-year time horizon.	**Primary Findings:**<br>• Screening for and treating postpartum depression and psychosis produced 29 more healthy women at a cost of $943 per woman.<br>• The incremental cost-effectiveness ratios of the intervention branch compared to usual care were $13,857 per QALY	This economic modelling study modelled the cost-effectiveness of physicians screening for and treating postpartum depression and psychosis in partnership with a psychiatrist.

		**Setting:** USA healthcare setting.<br><br>**Dates of data collection:** data were obtained from literature published between 1995 and 2015.	gained (below the commonly accepted willingness to pay threshold of $50,000/QALY gained) and $10,182 per remission achieved.<br>• These results were robust in both the deterministic and probabilistic sensitivity analyses of input parameters.<br><br>**Additional Findings:**<br>Screening for and treating postpartum depression is a cost-effective intervention and should be considered as part of usual postnatal care, which aligns with the recently proposed recommendations from the U.S. Preventive Services Task Force.	

Data extraction table for studies including maternal health and well-being

Citation (Country)	Study Details	Participants and setting	Key findings	Observations
Chojenta et al (2019)<br><br>(Chojenta et al., 2019)<br><br>Australia	**Study design:** Cross-sectional<br><br>**Data collection methods:** Health economics modelling study.<br><br>Data were taken from the Australian Longitudinal Study on Women's Health (ALSWH), an ongoing population-based study of health and well-being.	**Sample size:12,689**<br><br>**Participants:** Three cohorts of women born 1973–78, 1946–1951 and 1921–1926, with a fourth cohort born in 1989–1995 added in 2012.<br><br>**Setting:** Australian healthcare setting.<br><br>**Dates of data collection:** 1921 to 1995	**Primary findings:**<br>The healthcare costs for postnatal women who had poor mental health prior to birth was $1,792 (AUSD). This is on average 11% more than for mothers with no previous history of poor mental health.	This modelling study from Australia, utilising cohort data from 1921 to 1995 found that the healthcare costs for postnatal women who had poor mental health prior to birth was $1,792 (AUSD). This is on average 11% more than for mothers with no previous history of poor mental health.
Morrell et al (2000)<br><br>[34]<br><br>**UK**	**Study Design:** RCT<br><br>**Type of intervention [*exposure*]:**<br>Up to 10 home visits in the first postnatal month of up to three hours duration by a community postnatal support worker.<br><br>Impact of community postnatal support worker in addition to usual community midwife care on rest and recovery, health status, satisfaction with services and NHS Resource use and costs.<br><br>**Data collection methods:** Postal questionnaires (including SF36 and EPDS).	**Sample size:** 623<br><br>**Participants:** Postnatal women delivering at a university hospital<br><br>**Setting:** Home and community<br><br>**Dates of data collection:** Recruitment on labour wards from October 1996 to November 1997	**Primary Findings:** 551 completed 6 weeks questionnaire, 493 at 6 months. No evidence of use of fewer NHS services by women using the support worker versus controls at 6 weeks or 6 months.<br>Additional costs per woman at 6 weeks of £179.58 mostly due to support worker training (p<0.001).<br><br>**Additional Findings:** No diff primary outcome at 6 weeks but p<0.05 for physical and social functioning and p=005 EPDS for controls.<br>No difference in SF36 health status scores, EPDS scale or Duke Functional Social Support scale, rate of breastfeeding).	This study found that there were no savings to the NHS over six months after the introduction of a community support worker service and no improvement to the health status among the women in the intervention group, which was measured by an SF-36 questionnaire. At six weeks, the mean total NHS costs were £975▫ for the intervention group and £700 for the control group. At six months, the figures were £1,250 and £980, respectively.
Ride (2018)	**Study Design:** Modelling study (health economics)	**Date of model:** 2018<br><br>The models were developed using TreeAge Pro 2015 software (TreeAge Software, Inc.,	**Primary Findings:** The results suggest that broader boundaries, particularly extension of the time horizon, could make substantial differences to	By ignoring broader sets of costs and outcomes, resources in postnatal mental health may be misallocated, and as a result, some women may not benefit as

(Ride, 2018)<br><br>**UK**	**Data collection methods:** Decision analytic modelling	Williamstown, MA, USA). The population of interest was postnatal women and their children in the United Kingdom, because much of the data came from that setting; this gave an explicit societal threshold of £20,000 to £30,000 per QALY for cost-effectiveness analysis in health care. A health sector perspective was taken, except for the children's model, which expanded to a public sector perspective to accommodate educational costs. A discount rate of 3.5% was applied to costs and QALYs, with discounting applied back to the child's birth. All costs were converted to 2014 pounds sterling.	estimated cost-effectiveness. Inclusion of family effects without extension of the time horizon had little impact, but where a longer time horizon was used, family effects could make a significant difference to the conclusions drawn from cost-effectiveness analysis<br><br>**Additional Findings:**<br>The authors note that it is important not only to consider caregiving but also family health effects in the outcomes of maternal health studies.	much from interventions that might be cost-effective given a broader time-horizon.
