# Supplementary material for: Health economic evaluations of preventative care for perinatal anxiety and associated disorders: a rapid review
Source: BMJ Open. 2024 Feb 27;14(2):e068941. doi: 10.1136/bmjopen-2022-068941 (PMC10900391; doi:10.1136/bmjopen-2022-068941)
Supplement: Author's manuscript [file bmjopen-2022-068941.draft_revisions.pdf]

BMJ Open is committed to open peer review. As part of this commitment we make the peer review history of every article we publish publicly available.

When an article is published we post the peer reviewers' comments and the authors' responses online. We also post the versions of the paper that were used during peer review. These are the versions that the peer review comments apply to.

The versions of the paper that follow are the versions that were submitted during the peer review process. They are not the versions of record or the final published versions. They should not be cited or distributed as the published version of this manuscript.

BMJ Open is an open access journal and the full, final, typeset and author-corrected version of record of the manuscript is available on our site with no access controls, subscription charges or pay-per-view fees (http://bmjopen.bmj.com).

If you have any questions on BMJ Open's open peer review process please email info.bmjopen@bmj.com

BMJ Open

Cost-effectiveness of preventative care for perinatal anxiety and associated disorders: A rapid review

Journal:	*BMJ Open*
Manuscript ID	bmjopen-2022-068941
Article Type:	Original research
Date Submitted by the Author:	11-Oct-2022

Complete List of Authors:	Pisavadia, Kalpa; Bangor University, School of Medical and Health Sciences<br>Spencer, Llinos; Bangor University, Centre for Health Economics and Medicine Evaluation<br>Tuersley, Lorna; Bangor University,<br>Ayers, Susan; City University,<br>Coates, Rose ; City University of London,<br>Edwards, Rhiannon; Bangor University, Centre for Health Economics & Medicines Evaluation

Keywords:	HEALTH ECONOMICS, PREVENTIVE MEDICINE, MENTAL HEALTH

SCHOLARONE™
Manuscripts

I, the Submitting Author has the right to grant and does grant on behalf of all authors of the Work (as defined in the below author licence), an exclusive licence and/or a non-exclusive licence for contributions from authors who are: i) UK Crown employees; ii) where BMJ has agreed a CC-BY licence shall apply, and/or iii) in accordance with the terms applicable for US Federal Government officers or employees acting as part of their official duties; on a worldwide, perpetual, irrevocable, royalty-free basis to BMJ Publishing Group Ltd ("BMJ") its licensees and where the relevant Journal is co-owned by BMJ to the co-owners of the Journal, to publish the Work in this journal and any other BMJ products and to exploit all rights, as set out in our licence.

The Submitting Author accepts and understands that any supply made under these terms is made by BMJ to the Submitting Author unless you are acting as an employee on behalf of your employer or a postgraduate student of an affiliated institution which is paying any applicable article publishing charge ("APC") for Open Access articles. Where the Submitting Author wishes to make the Work available on an Open Access basis (and intends to pay the relevant APC), the terms of reuse of such Open Access shall be governed by a Creative Commons licence – details of these licences and which Creative Commons licence will apply to this Work are set out in our licence referred to above.

Other than as permitted in any relevant BMJ Author's Self Archiving Policies, I confirm this Work has not been accepted for publication elsewhere, is not being considered for publication elsewhere and does not duplicate material already published. I confirm all authors consent to publication of this Work and authorise the granting of this licence.

For peer review only

Cost-effectiveness of preventative care for perinatal anxiety and associated disorders: A rapid review

Miss Kalpa Pisavadia Kalpa.pisavadia@bangor.ac.uk

Dr Llinos Haf Spencer L.spencer@bangor.ac.uk

Dr Lorna Tuersley Lorna.tuersley@gmail.com

Dr Rose Coates Rose.coates@city.ac.uk

Professor Susan Ayers Susan.ayers.1@city.ac.uk

Professor Rhiannon Tudor Edwards R.t.edwards@bangor.ac.uk

Corresponding author: Kalpa Pisavadia Research Project Support Officer, Centre for Health Economics and Medicines Evaluation (CHEME), Ardudwy Hall, Normal Site, Bangor University, Bangor, Gwynedd, Wales, UK, LL57 2PZ. Email: Kalpa.pisavadia@bangor.ac.uk

For peer review only

Abstract

Aim: Perinatal mental health (PMH) problems affect one in five women and cost the United Kingdom (UK) £8.1 billion for every year of births, with 72% of this cost due to the long-term impact on the child. The aim of this rapid review was to evaluate the cost impact of services supporting perinatal mental health on the National Health Service (NHS) and similar systems.

Methodology: This study adopted a rapid review approach, using sections of the standard systematic review process to generate quality evidence. This methodology features a systematic database search, PRISMA diagram, screening of evidence, data extraction, critical appraisal, and narrative synthesis.

Results: Databases yielded 3,212 results published between January 2000 and July 2022. Titles and abstracts were screened, and an additional four papers were retrieved from existing systematic reviews. Of these twenty-one included papers there were economic evaluations (n=8), modelling studies (n=6), SR's (n=4), randomised controlled trials (RCT) (n=2), a cohort study (n=1).

Discussion: The results indicate a lack of economic evaluation specifically for perinatal anxiety, with most study articles focusing on postnatal depression (PND). Interventions to prevent postnatal mental health problems were found to be cost-effective. Modelling studies have also been conducted, which suggest that treating PND with counselling would be cost-effective.

Conclusion: The costs of not intervening in maternal mental health outweigh the costs of preventative interventions. Preventative measures such as screening and counselling for maternal mental health are shown to be cost-effective interventions to improve outcomes for women and children.

Key words: preventative, life-course, perinatal anxiety, postnatal depression, cost of illness, cost-effectiveness, economic modelling.

Article summary

Strengths and limitations of this study

- This review found no RCTs including economic evaluation of perinatal anxiety, which indicates an evidence gap.
- The strength of this rapid review is that it has highlighted costs associated with perinatal mental health interventions in a rigorous, novel way which will benefit the NIHR funded (Award number: NIHR133727), Map Alliance Project team with the economic evaluation for that study (currently in progress).
- Although there are several economic evaluations on perinatal mental health care from the USA, evidence from the UK is limited.
- There is an absence of health economic studies describing the range of public sector costs and costs to individuals from Scotland and Wales in relation to perinatal anxiety.
- Although health economic studies are showing the benefits of investing in PND, there are no published UK-based RCTs investigating perinatal mental

For peer review only - http://bmjopen.bmj.com/site/about/guidelines.xhtml

health interventions, which include information on costs (RCT's is the most scientifically rigorous method of hypothesis testing available and is regarded as the gold standard trial for evaluating the effectiveness of interventions [1]).

Introduction

The perinatal period refers to pregnancy and the first 12 months after childbirth [2]. One in five women experiences mental health problems during this time and the cost is estimated to be £8.1 billion for every year of births in the United Kingdom (UK) [3]. Maternal mental health problems include postnatal depression (PND) (also known as Postpartum Depression (PPD) internationally), characterised by depressed mood and anxiety, feelings of inadequacy, and impaired infant bonding [4]. More severe maternal mental health issues such as postpartum psychosis, can present with feelings of agitation, confusion or even hallucinations and delusions [5]. Crucially, suicide is the leading cause of maternal death in the perinatal period [6]. It is, thus, imperative that proactive planning and cost-effective preventative solutions are a public policy priority.

The Maternal Mental Health Alliance (2020) warns that the COVID-19 pandemic may lead to a potential increase in perinatal mental health (PMH) difficulties. A recent scoping review on the impact of the COVID-19 pandemic on maternal and perinatal health found that during pregnancy, self-reported rates of clinically relevant anxiety and depressive symptoms were higher among pregnant women compared to pre-pandemic levels [7]. Women who experience non-health-related stressors such as marital, housing, and financial difficulties or live in economically deprived areas, were already at higher risk of PMH issues prior to the pandemic [8]. The pandemic has further exacerbated the risk of impaired mental health due to limited antenatal care, reduced family support, social distancing and quarantine rules. These factors, in combination with anxieties surrounding the transmission of the COVID-19 disease, have been found to significantly impact maternal mental health [7].

Untreated maternal mental illness not only impacts mothers, but also adversely impacts their children, significantly contributing to wider societal and National Health Service (NHS) costs. Of the total costs of perinatal mental health difficulties in the UK, 72% is due to the long-term impact on the child [3]. An economic evaluation of a South London cohort found that for each child exposed to maternal perinatal depression, public sector costs exceeded £3,030. Costs due to reduced earnings were £1,400 per child, and health-related quality of life loss was valued at £3,760 per child [9].

Public sector costs are likely to be significantly reduced by utilising a prevention strategy to reduce the incidence of poor maternal mental health [9]. Decreased maternal and infant bonding, reduced breastfeeding initiation rates and duration, low birth weight, and poorer child growth have been associated with PND [10]. The regression analyses from an Australian cohort study revealed that children of mothers experiencing sub-clinical and increasing and persistently high depressive symptoms were twice as likely to have emotional and behavioural difficulties than

For peer review only - http://bmjopen.bmj.com/site/about/guidelines.xhtml

children of mothers reporting minimal symptoms [11]. Delayed or impaired cognitive, linguistic, physical, and psychological health development have been reported in infants and children with mothers with PND (Moore Simas et al., 2020). There is also a risk of intergenerational transmission of socio-economic disadvantage in which maternal mental illness impacts the child's quality of life by having a long-term adverse effect on education and employment prospects [9,12].

Despite the long-term risks of untreated maternal mental health issues, as of 2014 in the UK, only 30-50% of women with PMH problems were identified, and only 7% were referred to specialist care [3]. Most women with PMH problems did not access care [3]. This may have been particularly the case for women with mild to moderate PMH problems or less commonly recognised problems, such as anxiety, obsessive-compulsive disorder (OCD), or post-traumatic stress disorder (PTSD) [3]. Furthermore, access to care may also be limited by maternal time constraints and fears of being judged (Posmontier et al., 2016). Web-based approaches for delivering interventions could be a promisingly cost-effective solution in supporting mothers in the perinatal period by widening access to care, which hospitals could adopt as postnatal care support. A recent cost-effectiveness study, within a randomised controlled trial (RCT), evaluated a web-based approach for delivering a psychoeducational intervention [14]. This web-based approach was not only cost-effective in supporting first-time mothers, but also had the best improvements in self-efficacy, social support, and psychological well-being of women in Singapore.

The National Institute for Health and Care Excellence (National Institute for Health and Care Excellence, 2022) recommend postnatal care for up to eight weeks after birth. Since 2015, it has been recommended that UK midwives carry out emotional well-being checks at antenatal check-ups and at each postnatal contact up to eight weeks after birth. Women should be asked about their emotional wellbeing, what family and social support they have and their usual coping strategies for dealing with day-to-day matters. In 2018, the National Collaborating Centre for Mental Health worked with NICE to develop the Perinatal Mental Health Care Pathway [15]. The guidance in that report follows a process agreed upon by NICE and sets out pathways to deliver a strategic transformation of perinatal mental health care. Psychological interventions, either alone or in conjunction with pharmacological treatment, are recommended for complex or severe mental health problems following referral to a specialist community perinatal mental health team [2].

Since 2015 there have been improvements to funding plans and commitments in the provision of more specialist Community Perinatal Mental Health Services across the UK. For example, in 2019, the Scottish Government revealed that £52 million would be spent on improving access to perinatal and infant mental health services, and from 2018 to 2020, the Welsh Government increased recurrent annual funding from £1.5 million to £2.5 million for specialist PMH services [16]. In England, the Government committed £365 million to provide specialist perinatal community services across the country as announced by NHS England in April 2019 (National Institute of Health and Care Excellence, 2022). However, it is questionable whether there is sufficient funding for long-term plans and where the investment for the workforce across the UK will come from [16].

For peer review only - http://bmjopen.bmj.com/site/about/guidelines.xhtml

Aim

The aim of this rapid review was to evaluate evidence relating to the cost impact of health services within the NHS and similar healthcare systems for perinatal anxiety and associated disorders. The full protocol for this rapid review is available from PROSPERO [18].

Methods

This study adopted a rapid review approach, utilising sections of the standard systematic review process to generate quality evidence in a shorter timeframe. This methodology follows the minimum requirements for rapid reviews, featuring a systematic database search, PRISMA diagram [19] screening of evidence, data extraction, critical appraisal and narrative synthesis. This revised methodology is used by the Wales COVID-19 Evidence Centre [20–22].

Patient and Public Involvement

No patient involvement

Search Strategy

The key evidence sources of this rapid review included PubMed, Cumulative Index to Nursing and Allied Health Literature (CINAHL), Cochrane Library, Applied Social Sciences Index and Abstracts (ASSIA), PsycINFO and MEDLINE. The search terms consisted of words related to perinatal anxiety and/or depression, health and psychiatric services and economic evaluation terms. The searches were conducted on 23rd April 2022. Mendeley reference management software was used to manage study articles found and remove duplicates. See supplementary material for the full search strategy.

The eligibility criteria for the review are presented in Table 1 and are based on the Population, Intervention, Comparison and Outcome (PICO) framework [23]. This consisted of peer-reviewed economic evaluations of perinatal anxiety and associated disorders such as PND and PTSD from Organisation for Economic Co-operation and Development (OECD) countries in English published after January 2000.

For peer review only - http://bmjopen.bmj.com/site/about/guidelines.xhtml

Table 1: Participants, Intervention/exposure, Comparator and Outcomes (PICO) framework

**Question**	
What is the cost of care for women experiencing perinatal anxiety and associated disorders?	
**Participants**	Pregnant women or perinatal women
**Intervention / exposure**	Perinatal anxiety and associated disorders
**Comparator**	No comparator
**Outcomes**	Costs of primary care and support services for women experiencing perinatal anxiety and associated disorders
**Study Considerations**	
Primary, secondary, grey literature, preprints	
**Databases**	
PubMed, CINAHL, Cochrane Library, ASSIA, PsycINFO, MEDLINE	

Selection of studies

One reviewer (KP) independently selected potentially eligible studies based on a screening of titles and abstracts. Two reviewers (LHS and KP) selected additional studies from existing systematic reviews. The full texts of selected studies were assessed for eligibility by three reviewers (KP and LHS, with mediation by LT) in the data extraction process.

Data extraction

Data extraction and study quality assessment were performed by three reviewers (KP, LHS, LT). Data was collected on country, study design, intervention type, data collection methods and dates, sample size, and type of participants (See supplementary material 1 for results tables).

Quality assessment

The quality assessment was undertaken by two reviewers (LHS and KP) and four papers were checked by a third reviewer for quality assurance purposes (LT). The Drummond checklist [24] was used for the quality appraisal of health economic papers and the Checklist for critical appraisal and data extraction for systematic reviews of prediction modelling studies (CHARMS) checklist was used for the

For peer review only - http://bmjopen.bmj.com/site/about/guidelines.xhtml

modelling studies [25]. The Joanna Briggs Institute (JBI) critical appraisal tools were used for the quality appraisal of systematic reviews, randomised clinical trials, cohort studies and cross-sectional studies [26–28] (see supplementary materials 2).

[Insert figure 1 here]

For peer review only

For peer review only - http://bmjopen.bmj.com/site/about/guidelines.xhtml

Results

Searches of databases yielded 3212 results, of which 1226 duplicates were removed. The remaining 1986 results were screened against titles and abstracts, and an additional four papers were retrieved from existing systematic reviews. A total of 28 papers met the criteria for full-text screening. Seven papers were excluded due to not being able to access the full text (n=4), ineligible study design (n=1), or lack of relevancy (non-OECD country*)* (n=2). Twenty-one study articles were included in the final rapid review (see Figure 1 and Table 2). Seventeen of these were identified from database searchers, and four were identified from included Systematic Reviews (SR's).

Of these twenty-one included papers there were economic evaluations (n=8), modelling studies (n=6), SR's (n=4), randomised controlled trials (RCT) (n=2), a cohort study (n=1). All included study articles were peer-reviewed. The included study articles were separated into five sub-categories: children, prevention, cost of maternal health, cost of single interventions, and comparison cost of interventions. The following discussion provides a more detailed overview of the findings (see Table 2).

Table 2: Map of maternal cost of illness studies by evidence type (including studies on depression, anxiety and maternal health and wellbeing)

Type of Evidence	Cost of Illness studies					
	**Children**	**Prevention**	**Cost of maternal health**	**Cost of single interventions**	**Comparison cost of interventions**	**Number of studies**
**Randomised Controlled Trial (RCT)**				Morrell et al. (2000)	Grote et al. (2017)	**2**
**Systematic Review (SR)**		Moran et al. (2020)		Morrell et al. (2016)	Camacho and Shields (2018)	
					Gurung et al., (2018)	**4**
**Cohort study**	Moore Simas et al. (2020)					**1**
**Economic evaluation**		Petrou et al. (2006)	Petrou et al. (2002)		Henderson et al. (2019)	
		Ride et al. (2016)	Dagher et al. (2012)			
		Ride (2018)	Ammerman et al. (2016)			
			Roberts et al. (2001)			**8**
**Economic modelling studies**	Bauer et al. (2015)	Counts et al. (2022)	Franta et al. (2022)	Stevenson et al. (2010)		
		Wilkinson et al. (2017)	Chojenta et al. (2019)			**6**
**Total number of studies**	**2**	**6**	**6**	**3**	**4**	**21**

For peer review only - http://bmjopen.bmj.com/site/about/guidelines.xhtml

The included papers are organised under three different themes. The first theme is studies including perinatal anxiety, the second theme is perinatal depression, and the third theme is perinatal health and wellbeing. These included studies are detailed below.

Studies including perinatal anxiety

This review found only two studies focussing on perinatal anxiety [31,32]. Camacho and Shields conducted a systematic review of eight studies focussing on maternal mental health in the UK. This review searched for economic evaluations of interventions for the prevention or treatment of perinatal anxiety and depression (PAD), intending to guide researchers and commissioners of perinatal mental health services toward potentially cost-effective strategies. Camacho and Shields (2018) found that two interventions were likely to be cost-effective, in which both incorporated identification plus treatment of PND. These treatments included health visitor screening with counselling, GP and psychiatrist collaborative screening and treatment. This systematic review also found that psychiatric day hospital treatment, health visitor counsellors, and telephone-delivered peer support are possibly cost-effective.

The second study found on perinatal anxiety was an economic evaluation that consisted of a cost-effectiveness and cost-utility analysis of the What Were We Thinking (WWWT) intervention which was conducted alongside a cluster-randomised controlled trial by Ride et al. (2016). WWWT is a psychoeducational intervention targeted at the partner relationship, management of infant behaviour and parental fatigue for the prevention of postnatal maternal mental health problems. Although WWWT shows promise as a preventive intervention there is uncertainty over its cost-effectiveness as the analysis showed no statistically significant difference between the intervention and control groups in costs or outcomes. The intervention was estimated to cost £74.48[1] per participant.

Studies including perinatal depression

This review found fifteen studies focussing on perinatal depression [4,5,9,10,29,33–37,39–42,46]. A cross-sectional study from the USA conducted between 2006 and 2011 investigated the out-of-pocket expenses and insurer expenses of depressed mothers compared to non-depressed mothers [33]. Depressed mothers were more likely to incur insurer and out-of-pocket expenses (£1,285 vs £853 □□) and have higher insurer expenses (£10,485 vs £7,508□□). A study by Bauer et al. (2015) used the perspective of the public sector, individuals, and society to examine some of the outcomes and long-term economic implications experienced by offspring who have been exposed to perinatal depression in a South London cohort. Bauer et al. (2015) found that for each child exposed to perinatal depression, public sector costs exceeded £3,380□, costs due to reduced earnings were £1,562□, and health-related quality of life loss was valued at £3760□.[2]

1 □□ Prices have been inflated and converted to GBP [53]

2 □ Prices have been inflated to 2021 prices [54].

For peer review only - http://bmjopen.bmj.com/site/about/guidelines.xhtml

A decision analytic model used a simulated cohort of 1,000 Medicaid-enrolled pregnant individuals to evaluate the health care costs for individuals receiving PND preventive intervention or not, for 1 to 5 years post-partum (Counts et al., 2022). This study found that providing preventive interventions for PPS resulted in an estimated 5-year saving of £602□□.[3]

Dagher et al., (2012) conducted a cross-sectional study in the USA which investigated expenditure on health care services from hospital discharge until 11 weeks postpartum. There was a significant difference in healthcare expenditure between depressed and non-depressed women. The Edinburgh Postnatal Depression Scale (EPDS) was used to measure depression [47]. The total cost of all mental health counselling visits for the depressed group (n=31) was £165□□ and the cost for the non-depressed group (n= 607) was £15.50□□ (in 2007). This was a statistically significant difference ($p < 0.001$).

Using a theoretical cohort of 180,000 individuals, Franta et al. (2022) developed a decision-analytic model using TreeAge Pro software to compare outcomes in pregnant adolescents who received versus did not receive counselling interventions. This study found that it is cost-effective to refer all pregnant adolescents for preventive counselling interventions. Within the theoretical cohort for counselling, there were 8,935 fewer cases of perinatal depression, 1,606 fewer cases of chronic depression, 166 fewer preterm deliveries, four fewer neonatal deaths, 20 fewer cases of sudden infant death syndrome (SIDS), and one fewer case of cerebral palsy. In total, there were 21,976 additional QALYs and cost savings of £183,463,169⅄, making it the dominant strategy that had better outcomes with lower costs.[4]

An RCT trial by Grote et al. (2017) compared a multicomponent collaborative care intervention for depression (MOMcare - a choice of brief interpersonal psychotherapy or pharmacotherapy or both) with enhanced maternity support services (MSS-Plus) in the public health system of Seattle, USA. The incremental benefit and cost and the net benefit for women with major depression and PTSD was estimated. When controlled for baseline depression severity, women with probable depression and PTSD in MOMCare had 68 more depression-free days over 18 months than those in MSS-Plus ($p<.05$). There was an additional £1,943□□ depression care cost per MOMCare participant with comorbid PTSD. The incremental net benefit of MOMCare was positive if depression free days was valued below £18□□. For women with probable major depression and PTSD, MOMCare had a significant clinical benefit over MSS-Plus, with only a moderate increase in health services cost.[5]

A systematic review of papers published between 2000 and 2015 found that a combination of PND screening and treatment was cost-effective and that treatments such as psychological therapy, facilitated self-help, and customised treatment was more cost-effective than standard care. This review also found positive results for preventive strategies which involved peer support or counselling and other specific

3 □□ Prices have been inflated and converted to GBP [52].

4 ⅄ Prices have been converted to GBP [55]

5 □□ Prices have been inflated and converted to GBP [52].

For peer review only - http://bmjopen.bmj.com/site/about/guidelines.xhtml

support. However, group cognitive behavioural therapy (CBT) was not found to be cost-effective compared to standard care in one study [37].

Henderson et al. (2019) conducted a cluster RCT of health visitors trained to assess PND and deliver psychological approaches to women at risk of depression plus either a cognitive behavioural approach or a person-centred approach weekly for eight weeks. A cost-effectiveness analysis was run parallel to this for all mothers at low risk of depression in accordance with the EPDS at six months postnatal. This study found that CBT had a marginally higher probability of being cost-effective than a person-centred approach.

A cohort study with a sample size of 135,678 mother-child pairs with and without PND exposure revealed similar findings (Moore Simas et al., 2020). The results of this analysis suggest that the health resource utilisation and costs over the first 24 months of life in children of mothers with PND exceeded that of children of mothers without evidence of PND £22,940□□ and £20,487□□, respectively. This was a significant difference of £2,453.[6]

A systematic review and meta-analysis conducted by the National Institute for Health and Care Research (NIHR) aimed to evaluate the clinical effectiveness, cost-effectiveness, acceptability, and safety of antenatal and postnatal interventions for pregnant and postnatal women to prevent PND postnatal depression [39]. This review found that the most beneficial and cost-effective interventions appeared to be midwifery redesigned postnatal care, person-centred approach (PCA) and interpersonal psychotherapy (IPT). Women valued seeing the same health worker, partners' involvement, and access to several visits from a midwife or health visitor trained in person-centred or cognitive-behavioural approaches [39].

A longitudinal study by Petrou et al. (2002) estimated the economic costs of PND in a geographically defined cohort of women at high risk of developing the condition with the use of an RCT to identify women considered to be of high risk. Unit costs were applied to estimates of health and social care resource use made by 206 women and their infants recruited from antenatal clinics, and net costs per mother-infant dyad over the first 18 months post-partum were estimated. This study found that costs were £587□ higher for women with PND than for women without PND. Economic costs were also higher for women with extended experiences of the condition.[7]

A cost-effectiveness analysis of preventive interventions, which consisted of counselling and support for the mother–infant relationship, targeted at women at high risk of developing PND, was conducted by Petrou, et al. (2006). This study found that given the negative impact of PND on later child development, preventive interventions are likely to be cost-effective even at relatively low willingness to pay thresholds for preventing one month of PND during the first 18 months post-partum. The mean health and social care costs were estimated at £3,345□ per mother–infant

6 □□ Prices have been inflated and converted to GDP [52].

7 □ Prices have been inflated to 2021 prices [54].

For peer review only - http://bmjopen.bmj.com/site/about/guidelines.xhtml

dyad in the preventive intervention group and £3,277□ per mother–infant dyad in the routine primary care group, providing a mean cost difference of £166□.[8]

A cross-sectional study of 1,250 mothers of infants in a Canadian setting used the EPDS to investigate the costs associated with perinatal depression [41]. It was found that costs were notably different for mothers with and without depression. The total cost for health and social care was £833□□ for mothers with depression and their infants, compared to £406□□ for those with lower depression scores. This was statistically a significant difference at $p < .01$.[9]

An economic evaluation conducted by Stevenson et al. (2010) compared the cost-effectiveness of group Cognitive Behavioural Therapy (gCBT) compared with routine primary care for women with PND in the UK. This economic evaluation found that gCBT does not appear to be cost-effective due to the lack of literature providing robust information. Only one study, an RCT, was deemed applicable to the decision problem.

A cost-effectiveness analysis found that screening for and treating post-partum depression is a cost-effective intervention and should be considered as a part of usual postnatal care [5]. This study followed a hypothetical cohort of 1,000 pregnant women experiencing one live birth over a 2-year time horizon. The analysis found that screening for and treating PND and psychosis produced 29 more healthy women at the cost of £938□□ per woman. The incremental cost-effectiveness ratios (ICERs) of the intervention branch compared to usual care were £13,702□□ per quality-adjusted life year (QALY) gained (below the commonly accepted willingness to pay threshold of $50,000/QALY gained) and $10,182 per remission achieved.

Studies including maternal health and wellbeing

This review found four studies relating to health and wellbeing of perinatal women [30,43,44,48].

A systematic review by Moran et al. (2020) estimated the economic burden of a range of common health problems associated with pregnancy and childbirth. This review found eight studies that reported incremental costs associated with antenatal or postnatal mental health problems. Among the four studies that examined costs during pregnancy, birth, or the immediate post-partum period, the estimated incremental costs of poor maternal mental health ranged from £422 to £742□□.

A RCT by Morrell et al. (2000) aimed to establish the relative cost-effectiveness of postnatal support in the community in addition to the usual care provided by the community midwives. Three hundred and eleven women were allocated to the intervention of up to 10 home visits by a community postnatal support worker within the first postnatal month for a duration of up to three hours. This study found that there were no savings to the NHS over six months after the introduction of a

---

8 □ Prices have been inflated to 2021 prices [52].

9 □□ Prices have been inflated and converted to GBP [56].

For peer review only - http://bmjopen.bmj.com/site/about/guidelines.xhtml

community support worker service and no improvement to the health status among the women in the intervention group, which was measured by an SF-36 questionnaire [49].At six weeks, the mean total NHS costs were £975□ for the intervention group and £700□ for the control group. At six months, the figures were £1,250□ and £980□, respectively.[10]

Authors have suggested that prenatal interventions that do not seem cost-effective in the short term may be cost-effective over a longer time horizon [45]. Ride (2018) noted that it is important to consider caregiving and family health effects in the outcomes of maternal health studies. By not including broader sets of costs and outcomes, resources in postnatal mental health may be misallocated. As a result, some women may not benefit as much from interventions that might be cost-effective given a broader time horizon.

A modelling study from Australia, published in 2019, utilised cohort data from 1921 to 1995 and found that the healthcare costs for postnatal women who had poor mental health prior to birth was £1,066⅄ [30]. This is on average 11% more than for mothers with no previous history of poor mental health.[11]

Discussion

This aim of this rapid review was to identify the costs of support, care and treatments for perinatal anxiety and associated disorders in the UK NHS and similar healthcare systems. Twenty-one papers were included in this review from Australia, Canada, Ireland, the USA and the UK, each examining maternal mental health.

The results indicate a lack of economic evaluation specifically for perinatal anxiety, with most study articles focusing on PND [42]. Only two included papers focussed on anxiety with one being a systematic review looking at anxiety alongside depression [31]. The other was an economic evaluation of a maternal mental health intervention. Treatments for maternal mental health in the WWWT intervention consisted of health visitors with psychiatric training and group sessions focusing on parenting confidence and emotional well-being with online and face-to-face components (Ride et al., 2016). The WWWT intervention shows promise as a preventive intervention. However, there is uncertainty as to its cost-effectiveness. The analysis showed no statistically significant difference between the intervention and control groups in costs or outcomes with the intervention estimated to cost £74.48[12] per participant.

The majority of the studies included (n=15) focussed on the cost of services and interventions for PND. The evidence suggests significant health resource costs outside of mental health services as well as social care costs for PND for mother and mother-infant dyad. Costs were significantly higher for children of mothers with PND than for children of mothers without PND. This was a statistically significant difference of £2,453 (p <.001) [10].

10 □ Prices have been inflated to 2021 prices [54].

11 ⅄ Prices have been converted to GBP [55]

12 □□ Prices have been inflated and converted to GBP [53]

For peer review only

Significantly, counselling was found to be a cost-effective, preventative intervention for high-risk groups such as pregnant adolescents [36]. Using a hypothetical cohort, Franta et al. (2022) found that counselling was a cost-effective preventative measure, leading to fewer cases of perinatal and chronic depression. Counts et al. (2022) estimated that group counselling (costing £114 per mother) cost around £73□ less than individual counselling (£187 per mother) for mothers with PND. Counts et al. (2022) found that screening for PND costs less than £2 per mother. Studies that combined screening for PND with an intervention were also found to be cost-effective resulting in 29 more healthy women at a cost of £938□□ per woman (Wilkinson et al., 2017). The incremental cost-effectiveness ratios of the intervention branch compared to usual care were $13,857 per QALY gained (below the commonly accepted willingness to pay threshold of $50,000/QALY gained) and $10,182 per remission achieved.

Within this rapid review, the EPDS, a validated measure for postnatal depression and anxiety [47], was the most frequently used instrument to detect perinatal and PND in the included studies, followed by the SF-36 scale (Ware, 2000), postal questionnaires such as the Ontario health survey, Health and Social Service Utilisation Questionnaire (HSUQ), blinded telephone assessments and medical records, Medicaid data, resource use logs completed by health visitors based on GP records, and prospective diaries and face-to-face interviews.

In summary, screening was found to be a relatively low-cost method of identifying women in need of mental health support during the perinatal period. Interventions to prevent postnatal mental health problems were found to be cost-effective (Ride et al., 2016). Also, two modelling studies found that treating PND with counselling would be cost-effective (Stevenson et al., 2010; Wilkinson et al., 2017).

Future research in this area should investigate how best to screen all mothers to prevent and treat further adverse outcomes such as anxiety, OCD, or PTSD [3]. Various psycho-social methods could be used to screen and provide treatment over the telephone, online or face-to-face. Interventions could be provided by a range of healthcare professionals such as midwives, health visitors, counsellors, psychologists, and psychiatrists. The effectiveness and cost-effectiveness of each intervention, including screening, should be evaluated.

Web-based approaches are already promising to be cost-effective solutions to support mothers in the perinatal period. Most women of childbearing age, including women who reside in rural areas, now have access to the internet in the UK and similar health care systems. There is concern regarding web-based interventions, for example, the lack of engagement could lead to significant drop out [50]. Being able to access support and treatment using online resources has widened access to care to postnatal care support. A recent cost-effectiveness study alongside an RCT in Singapore, evaluated a web-based approach for delivering a psychoeducational intervention (Zheng et al., 2022). This web-based approach was cost-effective in supporting first-time mothers and provided the best improvements in self-efficacy, social support, and psychological well-being of mothers in the perinatal period.

For peer review only - http://bmjopen.bmj.com/site/about/guidelines.xhtml

The MAP ALLIANCE, funded by the NIHR (Award ID: NIHR133727), project in the UK aims to examine the care offered and accessed by women experiencing perinatal anxiety and associated disorders. This study includes an economic component to evaluate the cost-of-service use for perinatal anxiety and associated disorders. It is anticipated that the MAP ALLIANCE study will lead to recommendations for accessible, integrated care acceptable to women. It will assist NHS commissioners and providers in designing and transforming services for perinatal women. This will increase the chances for women to receive better care to improve maternal and child outcomes [51].

Conclusion

However, as depression during pregnancy is strongly associated with both PND and anxiety following childbirth, study articles that found preventative treatment interventions to be cost-effective were included and reviewed. The findings from this review show that the costs of not intervening in maternal mental health far outweigh the costs of preventative interventions. Maternal mental health has significant long-term economic consequences in which children are affected well into adulthood regarding cognitive, psychological, and physical development, education, and career through the life-course [9,10]. Preventative measures, such as screening, combined with treatment, such as counselling, for maternal mental health are proven to be cost-effective interventions to improve outcomes for women and children.

Recommendations

It is recommended that:

- Mothers should be screened for maternal mental health issues to identify mothers at risk and provide treatment, leading to better outcomes for the mother and child dyad.
- Studies focussing on interventions for perinatal anxiety as distinct condition to other mental health issues such as depression should be conducted.
- The cost of interventions to reduce perinatal anxiety should be carried out.

Acknowledgements: The authors thank the wider MAP ALLIANCE team from City University of London and University of Stirling for input into the development of this review. We would also like to thank Yasmine Noorani, Academic Support Librarian at Bangor University, for her assistance in creating our search strategy. Additional thanks to Dr Catherine Lawrence for early input and feedback on this paper.

Contributors: The review was conceived by RTE, and the protocol was developed by LT, KP and LHS [18]; searches were undertaken by KP; article screening was carried out by KP and LHS with mediation by LT; quality appraisal was undertaken by KP, LHS and LT; data were interpreted by all authors; the manuscript was drafted by KP and LHS and critically reviewed by all authors.

Funding: This review is to compliment the MAP ALLIANCE study, funded by the National Institute for Health and Care Research (NIHR) (Award ID: NIHR133727).

Conflict of interest: All authors declare that they have no conflicts of interest.

For peer review only - http://bmjopen.bmj.com/site/about/guidelines.xhtml

Ethical approval:
This study does not involve human participants.

Data statement: Data extraction tables are available from the corresponding author on request.

For peer review only

For peer review only - http://bmjopen.bmj.com/site/about/guidelines.xhtml

References

[1] Akobeng AK. Principles of evidence based medicine. Arch Dis Child 2005;90:837–40. https://doi.org/10.1136/adc.2005.071761.

[2] NHS England and NHS Improvement. The Perinatal Mental Health Care Pathways NHS England and NHS Improvement. 2018.

[3] Bauer A, Personage M, Knapp M, Lemmi V, Adeleja B. Costs of perinatal mental health problems. London: 2014.

[4] Petrou S, Cooper P, Murray L, Davidson LL, Lawson C, Woolgar M, et al. Cost-effectiveness of a preventive counseling and support package for postnatal depression. Int J Technol Assess Health Care 2006;22:443–53. https://doi.org/10.1017/S0266462306051361.

[5] Wilkinson A, Anderson S, Wheeler SB. Screening for and Treating Postpartum Depression and Psychosis: A Cost-Effectiveness Analysis. Matern Child Health J 2017;21:903–14. https://doi.org/10.1007/s10995-016-2192-9.

[6] Howard LM, Flach C, Mehay A, Sharp D, Tylee A. The prevalence of suicidal ideation identified by the Edinburgh Postnatal Depression Scale in postpartum women in primary care: Findings from the RESPOND trial. BMC Pregnancy Childbirth 2011;11. https://doi.org/10.1186/1471-2393-11-57.

[7] Kotlar B, Gerson E, Petrillo S, Langer A, Tiemeier H. The impact of the COVID-19 pandemic on maternal and perinatal health: a scoping review. Reprod Health 2021;18. https://doi.org/10.1186/S12978-021-01070-6.

[8] Pilav S, Easter A, Silverio SA, de Backer K, Sundaresh S, Roberts S, et al. Experiences of Perinatal Mental Health Care among Minority Ethnic Women during the COVID-19 Pandemic in London: A Qualitative Study. Int J Environ Res Public Health 2022;19. https://doi.org/10.3390/IJERPH19041975.

[9] Bauer A, Pawlby S, Plant DT, King D, Pariante CM, Knapp M. Perinatal depression and child development: Exploring the economic consequences from a South London cohort. Psychol Med 2015;45:51–61. https://doi.org/10.1017/S0033291714001044.

[10] Moore Simas TA, Huang MY, Packnett ER, Zimmerman NM, Moynihan M, Eldar-Lissai A. Matched cohort study of healthcare resource utilization and costs in young children of mothers with postpartum depression in the United States. J Med Econ 2020;23:174–83. https://doi.org/10.1080/13696998.2019.1679157.

[11] Giallo R, Woolhouse H, Gartland D, Hiscock H, Brown S. The emotional–behavioural functioning of children exposed to maternal depressive symptoms across pregnancy and early childhood: a prospective Australian pregnancy cohort study. Eur Child Adolesc Psychiatry 2015;24:1233–44. https://doi.org/10.1007/s00787-014-0672-2.

For peer review only - http://bmjopen.bmj.com/site/about/guidelines.xhtml

[12] Hardcastle K, Bellis MA, Ford K, Hughes K, Garner J, Ramos Rodriguez G. Measuring the relationships between adverse childhood experiences and educational and employment success in England and Wales: findings from a retrospective study. Public Health 2018;165:106–16. https://doi.org/10.1016/J.PUHE.2018.09.014.

[13] Posmontier B, Neugebauer R, Stuart S, Chittams J, Shaughnessy R. Telephone-Administered Interpersonal Psychotherapy by Nurse-Midwives for Postpartum Depression. J Midwifery Womens Health 2016;61:456–66. https://doi.org/10.1111/JMWH.12411.

[14] Zheng Q, Shi L, Zhu L, Jiao N, Chong YS, Chan SWC, et al. Cost-effectiveness of Web-Based and Home-Based Postnatal Psychoeducational Interventions for First-time Mothers: Economic Evaluation Alongside Randomized Controlled Trial. J Med Internet Res 2022;24. https://doi.org/10.2196/25821.

[15] National Institute for Health and Care Excellence. Maternity and mental health 2022. https://www.nice.org.uk/about/what-we-do/into-practice/measuring-the-use-of-nice-guidance/impact-of-our-guidance/niceimpact-maternity/ch2-maternity-and-mental-health (accessed July 27, 2022).

[16] Maternal Mental Health Alliance. Map of Specialist Community Perinatal Mental Health Teams (UK). 2020.

[17] The National Institute of Health and Care Excellence. NICE | The National Institute for Health and Care Excellence 2022. https://www.nice.org.uk/ (accessed February 9, 2022).

[18] Spencer LH, Pisavadia K, Tuersley L, Edwards RT. A structured review of the costs and services for perinatal anxiety and associated disorders: MAP ALLIANCE. PROSPERO 2022 CRD42022347859. PROSPERO 2022.

[19] Page M, McKenzie J, Bossuyt P, Boutron I, Hoffmann T, Mulrow C. The PRISMA 2020 statement: an updated guideline for reporting systematic reviews. BMJ 2021. BMJ 2021. https://doi.org/doi: 10.1136/bmj.n71.

[20] Health and Care Research Wales. Impact of the COVID-19 pandemic on the health and access to health care of disabled people: a rapid review 2022. https://healthandcareresearchwales.org/impact-covid-19-pandemic-disabled-people (accessed May 10, 2022).

[21] Health and Care Research Wales. Modelling studies used to evaluate the effect of population-level non-pharmaceutical interventions on the reproduction number of Severe Acute Respiratory Syndrome Coronavirus 2 (SARS-CoV-2) 2022. https://healthandcareresearchwales.org/modelling-studies-used-evaluate-effect-population-level-non-pharmaceutical-interventions (accessed May 10, 2022).

For peer review only - http://bmjopen.bmj.com/site/about/guidelines.xhtml

[22] Tricco AC, Khalil H, Holly C, Feyissa G, Godfrey C, Evans C, et al. Rapid reviews and the methodological rigor of evidence synthesis. JBI Evid Synth 2022;Publish Ah:944–9. https://doi.org/10.11124/jbies-21-00371.

[23] Schardt C, Adams MB, Owens T, Keitz S, Fontelo P. Utilization of the PICO framework to improve searching PubMed for clinical questions. BMC Med Inform Decis Mak 2007;7:1–6.

[24] Drummond MF, Sculpher MJ, Claxton K, Stoddart GL, Torrance G W. Methods for the economic evaluation of health care programmes. 4th ed. Oxford: Oxford University Press; 2015.

[25] Moons KGM, de Groot JAH, Bouwmeester W, Vergouwe Y, Mallett S, Altman DG, et al. Critical Appraisal and Data Extraction for Systematic Reviews of Prediction Modelling Studies: The CHARMS Checklist. PLoS Med 2014;11. https://doi.org/10.1371/journal.pmed.1001744.

[26] Aromataris E, Fernandez R, Godfrey C, Holly C, Kahlil H, Tungpunkom P. Summarizing systematic reviews: methodological development, conduct and reporting of an Umbrella review approach. Int J Evid Based Healthc 2015 2015;13:132–40.

[27] Tufanaru C, Munn Z, Aromataris E, Campbell J, Hopp L. Systematic reviews of effectiveness. In: Aromataris E, Munn Z, editors. JBI Manual for Evidence Synthesis, JBI; 2020.

[28] Moola S, Munn Z, Tufanaru C, Aromataris E, Sears K, Sfetcu R, et al. Systematic reviews of etiology and risk. In: Aromataris E, Munn Z, editors. Manual for Evidence Synthesis, JBI; 2020.

[29] Grote NK, Simon GE, Russo J, Lohr MJ, Carson K, Katon W. Incremental benefit-cost of MOMCare: Collaborative care for perinatal depression among economically disadvantaged women. Psychiatric Services 2017;68:1164–71. https://doi.org/10.1176/APPI.PS.201600411.

[30] Chojenta C, William J, Martin MA, Byles J, Loxton D. The impact of a history of poor mental health on health care costs in the perinatal period. Arch Womens Ment Health 2019;22:467–73. https://doi.org/10.1007/S00737-018-0912-4.

[31] Camacho EM, Shields GE. Cost-effectiveness of interventions for perinatal anxiety and/or depression: A systematic review. BMJ Open 2018;8. https://doi.org/10.1136/bmjopen-2018-022022.

[32] Ride J, Lorgelly P, Tran T, Wynter K, Rowe H, Fisher J. Preventing postnatal maternal mental health problems using a psychoeducational intervention: the cost-effectiveness of What Were We Thinking. BMJ Ope 2016;6. https://doi.org/10.1136/bmjopen-2016.

[33] Ammerman RT, Chen J, Mallow PJ, Rizzo JA, Folger AT, van Ginkel JB. Annual direct health care expenditures and employee absenteeism costs in high-risk, low-income mothers with major depression. J Affect Disord 2016;190:386–94. https://doi.org/10.1016/J.JAD.2015.10.025.

For peer review only - http://bmjopen.bmj.com/site/about/guidelines.xhtml

[34] Counts NZ, Kuklinski MR, Wong V, Feinberg ME, Creedon TB. Comparison of Estimated Incentives for Preventing Postpartum Depression in Value-Based Payment Models Using the Net Present Value of Care vs Total Cost of Care. JAMA Netw Open 2022;5:e229401. https://doi.org/10.1001/JAMANETWORKOPEN.2022.9401.

[35] Dagher RK, McGovern PM, Dowd BE, Gjerdingen DK. Postpartum depression and health services expenditures among employed women. J Occup Environ Med 2012;54:210–5. https://doi.org/10.1097/JOM.0B013E31823FDF85.

[36] Franta G, Hersh AR, Cirino NH, Caughey AB. Prevention of perinatal depression with counseling in adolescents: a cost-effectiveness analysis. The Journal of Maternal-Fetal & Neonatal Medicine: The Official Journal of the European Association of Perinatal Medicine 2022. https://doi.org/10.1080/14767058.2022.2049746.

[37] Gurung B, Jackson LJ, Monahan M, Butterworth R, Roberts TE. Identifying and assessing the benefits of interventions for postnatal depression: A systematic review of economic evaluations. BMC Pregnancy Childbirth 2018;18. https://doi.org/10.1186/S12884-018-1738-9.

[38] Henderson C, Dixon S, Bauer A, Knapp M, Jane Morrell C, Slade P, et al. Cost-effectiveness of PoNDER health visitor training for mothers at lower risk of depression: Findings on prevention of postnatal depression from a cluster-randomised controlled trial. Psychol Med 2019;49:1324–34. https://doi.org/10.1017/S0033291718001940.

[39] Morrell CJ, Sutcliffe P, Booth A, Stevens J, Scope A, Stevenson M, et al. A systematic review, evidence synthesis and meta-analysis of quantitative and qualitative studies evaluating the clinical effectiveness, the cost-effectiveness, safety and acceptability of interventions to prevent postnatal depression. Health Technol Assess 2016;20:1–414. https://doi.org/10.3310/HTA20370.

[40] Petrou S, Cooper P, Murray L, Davidson LL. Economic costs of post-natal depression in a high-risk British cohort. British Journal of Psychiatry 2002;181:505–12. https://doi.org/10.1192/bjp.181.6.505.

[41] Roberts J, Sword W, Watt S, Gafni A, Krueger P, Sheehan D, et al. Costs of postpartum care examining associations from the Ontario mother and infant survey. Canadian Journal of Nursing Research 2001;33:19–34.

[42] Stevenson MD, Scope A, Sutcliffe PA. The Cost-Effectiveness of Group Cognitive Behavioral Therapy Compared with Routine Primary Care for Women with Postnatal Depression in the UKv he_720 580..584. Value in Health 2010;13:580–4. https://doi.org/10.1111/j.1524-4733.2010.00720.x.

[43] Moran PS, Wuytack F, Turner M, Normand C, Brown S, Begley C, et al. Economic burden of maternal morbidity – A systematic review of cost-of-illness studies. PLoS One 2020;15. https://doi.org/10.1371/JOURNAL.PONE.0227377.

For peer review only - http://bmjopen.bmj.com/site/about/guidelines.xhtml

[44] Morrell CJ, Spiby H, Stewart P, Walters S, Morgan A. Costs and effectiveness of community postnatal support workers: Randomised controlled trial. Br Med J 2000;321:593–8. https://doi.org/10.1136/BMJ.321.7261.593.

[45] Ride J. Setting the Boundaries for Economic Evaluation: Investigating Time Horizon and Family Effects in the Case of Postnatal Depression 2018. https://doi.org/10.1016/j.jval.2017.10.016.

[46] Henderson J. The cost effectiveness of prenatal care - ProQuest 1994. https://www.proquest.com/assia/docview/196971225/abstract/36297FA264EA438FPQ/1?accountid=14874 (accessed May 21, 2022).

[47] Cox JL, Chapman G, Murray D, Jones P. Validation of the Edinburgh postnatal depression scale (EPDS) in non-postnatal women. vol. 39. 1996.

[48] Ride J. Setting the Boundaries for Economic Evaluation: Investigating Time Horizon and Family Effects in the Case of Postnatal Depression. Value in Health 2018;21:573–80. https://doi.org/10.1016/j.jval.2017.10.016.

[49] Ware JE. SF-36 Health Survey Update. Spine (Phila Pa 1976) 2000;25:3130–9.

[50] Davis J, Ohan JL, Gibson LY, Prescott SL, Finlay-Jones AL. Understanding engagement in digital mental health and wellbeing programs for sub-clinical women in the perinatal period: A Systematic Review without Meta-Analysis (SWiM) (Preprint). J Med Internet Res 2022. https://doi.org/10.2196/36620.

[51] Ayers S, Coates R, Sinesi A, Best C, Shakespeare J, Hollins K, et al. OptiMising cAre for Perinatal Anxiety: evaLuation of heaLth servIce utilisAtioN, outComEs and costs (MAP ALLIANCE) - NIHR Funding and Awards. National Institute for Health and Care Research 2022. https://fundingawards.nihr.ac.uk/award/NIHR133727 (accessed August 16, 2022).

[52] U.S. Bureau of Labor Statistics. CPI Inflation Calculator 2022. https://www.bls.gov/data/inflation_calculator.htm (accessed August 18, 2022).

[53] Reserve Bank of Australia 2022.

[54] Bank of England. Inflation calculator 2022. https://www.bankofengland.co.uk/monetary-policy/inflation/inflation-calculator (accessed August 18, 2022).

[55] Google. Google currency converter 2022. https://www.google.com/search?q=currency+convertor&rlz=1C1GCEA_enGB991GB991&oq=currency+convertor&aqs=chrome..69i57j0i10i131i433l2j0i10l7.4286j1j3&sourceid=chrome&ie=UTF-8 (accessed September 5, 2022).

[56] Bank of Canada. Inflation Calculator 2022. https://www.bankofcanada.ca/rates/related/inflation-calculator/ (accessed August 18, 2022).

For peer review only - http://bmjopen.bmj.com/site/about/guidelines.xhtml

Figure 1. PRISMA study selection flowchart.

For peer review only

Figure 1. PRISMA study selection flowchart (Page et al., 2021b)

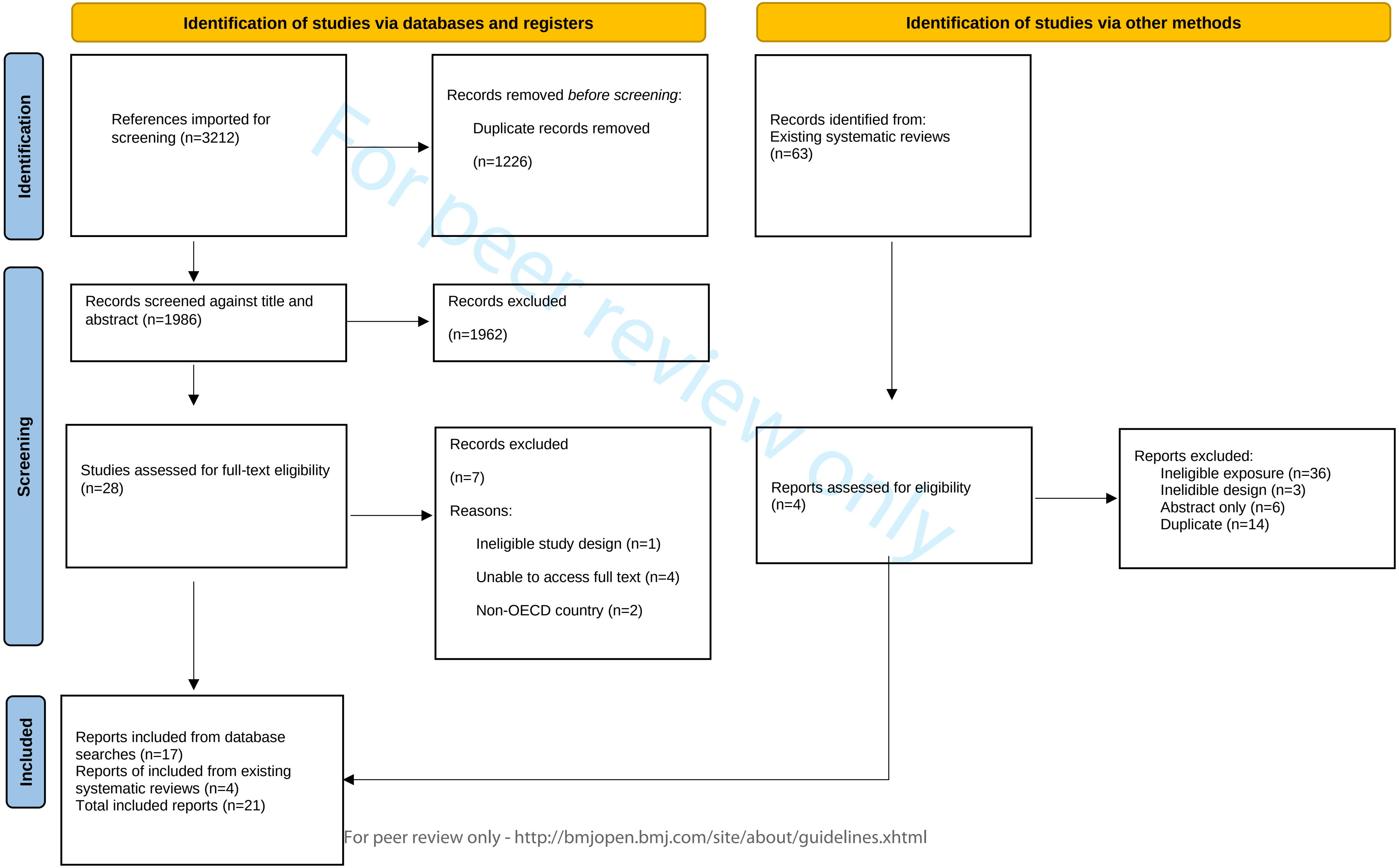

For peer review only

The study selection flow chart is shown as a PRISMA flow chart (Page et al., 2021a)

From: Page MJ, McKenzie JE, Bossuyt PM, Boutron I, Hoffmann TC, Mulrow CD, et al. The PRISMA 2020 statement: an updated guideline for reporting systematic reviews. BMJ 2021;372:n71. doi: 10.1136/bmj.n71. For more information, visit: http://www.prisma-statement.org/

For peer review only

For peer review only

Table 3: Data extraction table for studies including perinatal anxiety

Citation (Country)	Study Details	Participants and setting	Key findings	Observations
Camacho and Shields (2018)<br><br>[31]<br><br>**UK**	**Study Design:** SR of Cost-effectiveness studies of interventions for perinatal anxiety and/or depression<br><br>**Type of intervention [*exposure*]:** Perinatal anxiety and/or depression (PND)<br><br>**Data collection methods:** Search strategy within MEDLINE, PsycINFO and NHS Economic Evaluation and Health Technology Assessment databases	**Sample size:** 8 studies were included in the SR.<br><br>**Participants:** Mothers<br><br>**Setting:** Maternal health care setting in the UK.<br><br>**Dates of data collection:** January 2000 to September 2017.	**Primary Findings:**<br>• 8 studies met the inclusion criteria for the review: all but one focussed solely on PND in mothers.<br>• Interventions included prevention (n=3), treatment (n=3) or identification plus treatment (n=2).<br>• Two interventions were likely to be cost-effective, both incorporated identification plus treatment. Where the cost per QALY gained was reported, interventions ranged from being dominant (cheaper and more effective than usual care) to costing £39,875/QALY.	This SR from the UK found that two interventions were likely to be cost-effective, in which both incorporated identification plus treatment of PND. These treatments included health visitor screening with counselling, GP and psychiatrist collaborative screening and treatment. This systematic review also found that psychiatric day hospital treatment, health visitor counsellors, and telephone-delivered peer support were possibly cost-effective.
Ride et al (2016)<br><br>(Ride et al., 2016)<br><br>**Australia**	**Study Design:** Economic evaluation, including cost-effectiveness and cost-utility analyses, conducted alongside a cluster-randomised trial<br><br>**Type of intervention [*exposure*]:** What Were We Thinking (WWWT) - a psychoeducational intervention targeted at the partner relationship, management of infant behaviour and parental fatigue.<br><br>**Data collection methods:** Data were collected from participants via computer-assisted telephone interview at baseline (6 weeks postpartum)	**Sample size:** 359<br><br>**Participants:** English-speaking first-time mothers who had recently given birth and attended participating Maternal and Child Health Centres (MCHCs)<br><br>**Setting:** 48 Maternal and Child Health Centres in Victoria, Australia.<br><br>**Dates of data collection:** Baseline interviews took place between May 2013 and April 2014, and follow-up interviews between September 2013 and August 2014.	**Primary Findings:**<br>The intervention was estimated to cost $A118.16 per participant. The analysis showed no statistically significant difference between the intervention and control groups in costs or outcomes. The incremental cost-effectiveness ratios were $A36 451 per QALY gained and $A152 per percentage point reduction in 30-day prevalence of depression, anxiety and adjustment disorders. The estimate lies under the unofficial cost-effectiveness threshold of $A55 000 per QALY; however, there was considerable uncertainty surrounding the results, with a 55% probability that WWWT would be considered cost-effective at that threshold<br>**Additional Findings:**<br>The results suggest that, although WWWT shows promise as a preventive intervention for postnatal maternal mental health problems, further research is required to reduce the uncertainty over its cost-effectiveness as there were no statistically significant differences in costs or outcomes.	Cost-effectiveness analysis of the intervention, What Were We Thinking (WWWT), for the prevention of postnatal maternal mental health problems

For peer review only - http://bmjopen.bmj.com/site/about/guidelines.xhtml

	and follow-up (26 weeks postpartum).			

Table 4: Data extraction table for cross-sectional studies including maternal depression

Citation (Country)	Study Details	Participants and setting	Key findings	Observations
Ammerman et al (2016)<br><br>(Ammerman et al., 2016)<br><br>USA	**Study design:** Cross-sectional<br><br>**Data collection methods:** MEPS database, a subset of the National Health Interview Survey (NHIS) that includes information on health care utilisation and expenditures for the civilian, non-institutionalised population in the USA.	**Sample size:** 20,531<br><br>**Participants:** 2,310 high-risk mothers with depression and 18,221 high-risk mothers without depression<br><br>**Setting:** USA healthcare setting<br><br>**Dates of data collection:** 1996 to 2011	**Primary findings:**<br>Depressed mothers were more likely to incur insurer (0.88 vs. 0.80) and out of pocket expenses (0.86 vs. 0.77) and to have higher insurer expenses ($4916 vs. $3521) and out of pocket expenses ($786 vs. $522) (in 2015).<br>**Additional findings:**<br>A higher proportion of the depressed sample was Caucasian and in relatively worse health than women from other ethnic groups. The depressed sample was more likely to have public insurance, to be English-speaking and to have a usual health care provider.	This cross-sectional study from the USA conducted between 2006 and 2011 investigated the out-of-pocket expenses and insurer expenses of depressed vs non-depressed mothers. Depressed mothers were more likely to incur insurer and out of pocket expenses and to have higher insurer expenses ($4916 vs. $3521) and out of pocket expenses ($786 vs. $522) (in 2015).
Dagher et al (2012)<br><br>(Dagher et al., 2012<br><br>USA	**Study design:** Cross-sectional<br><br>**Data collection methods:** Prices of service use and EPDS	**Sample size:** 638 women.<br><br>**Participants:** Women receiving maternal healthcare services, from hospital discharge to 11 weeks postpartum.<br><br>**Setting:** USA healthcare setting.<br><br>**Dates of data collection:** The year 2001.	**Primary findings:**<br>The total cost of all mental health counselling visits for the depressed group n =31 was $138 and the cost for the non-depressed group n= 607 was $13. This was a statistically significant difference (p < 0.001).<br><br>**Additional findings:** The total cost of emergency department visits for the postpartum women was $84 for the depressed group n = 31 and $13 for the non-depressed group n = 607. This was a statistically significant difference (p < 0.001).	The Dagher et al., (2012) cross-sectional study from the USA investigated expenditure from health care service from discharge until 11 weeks postpartum. There was a significant difference in healthcare expenditure between depressed and non-depressed women. The EPDS was used to measure depression. The total cost of all mental health counselling visits for the depressed group n =31 was $138 and the cost for the non-depressed group n= 607 was $13. This was a statistically significant difference (p < 0.001).

Table 5: Data extraction table for economic evaluations on maternal depression

Citation (Country)	Study Details	Participants and setting	Key findings	Observations
Bauer et al (2015)<br><br>(Bauer et al., 2015)<br><br>UK	**Study Design:** The economic analysis takes a life-course perspective from the viewpoints of the public sector, individual and society. The study analysed the effects of perinatal depression on child development outcomes of children at ages 11 and 16 years from the community-based South London Child Development Study. Economic consequences were attached to those outcomes through simple decision-analytic techniques, building on evidence from studies of epidemiology, health-related quality of life, public sector costs and employment.	**Sample size:** 120<br><br>**Participants:** Mothers and children<br><br>**Setting:** Two antenatal clinics in the UK<br><br>**Dates of data collection:** January to December 1986	**Primary Findings:**<br>Additional risks that children exposed to perinatal depression develop emotional, behavioural or cognitive problems ranged from 5% to 21%. In addition, there was a high risk (24%) that children would have special educational needs.<br><br>For each child exposed to perinatal depression, public sector costs exceeded £3,030, costs due to reduced earnings were £1,400 and health-related quality of life loss was valued at £3,760.	The study examined some of the outcomes and long-term economic implications experienced by offspring who have been exposed to perinatal depression.
Counts et al (2022)<br><br>(Counts et al., 2022)<br><br>USA	**Study Design:** Modelling study. A decision analytic model used a simulated cohort of 1,000 Medicaid-enrolled pregnant individuals. Health care costs for individuals receiving postpartum depression preventive intervention or not, over 1 or 5 years postpartum, in a variety of scenarios, including varying rates of Medicaid churn (i.e., transitions to a new Medicaid managed care plan, commercial insurance plan, or loss of coverage) were estimated for the period 2020 to 2025. The model was developed between March 5 2021 and July 30 2021.<br><br>**Type of intervention [*exposure*]:** Individual counselling and group-based counselling.<br><br>**Data collection methods:** Simulation based on collected Medicaid data.	**Sample size:** 1,000<br><br>**Participants:** simulated cohort of 1,000 Medicaid enrolled pregnant individuals<br><br>**Setting:** USA healthcare system.<br><br>**Dates of data collection:** Model developed between March 5 2021 and July 30 2021.	**Primary Findings:**<br>The main outcome was the amount of clinician incentive shared in a Value-based payment (VBP) model from providing preventive interventions. The likelihood of the health care payer realising a positive return on investment if it shared 50% of 5-year expected savings with a clinician up front was also measured.<br><br>The simulated cohort was designed to be reflective of the demographics characteristics of pregnant individuals receiving Medicaid; however, no specific demographic features were simulated. Providing preventive interventions for postpartum depression resulted in an estimated 5-year savings of $734.12 (95% credible interval [CrI], $217.21-$1235.67) per person. Without health insurance churn, sharing 50% of 5-year expected savings could offer more than double the financial incentives for clinicians to prevent postpartum depression compared with traditional VBP ($367.06 [95% CrI, $108.61-$617.83] vs	This economic modelling study found that providing preventive interventions for PND resulted in an estimated 5-year saving of £602□

			$177.74 [95% CrI, $52.66-$296.60], respectively), with a high likelihood of positive return for the health care payer (91%). As health insurance churn increased, clinician incentives from sharing estimated savings decreased (73% reduction with 50% annual churn).	
Franta et al (2022)<br><br>(Franta et al., 2022)<br><br>USA	**Study Design:** Modelling study<br>**Type of intervention [*exposure*]:** Comparison of outcomes in pregnant adolescents who received versus did not receive counselling interventions<br><br>**Data collection methods**: Decision-analytic model using TreeAge Pro software	**Sample size:** Theoretical cohort of 180,000 individuals<br><br>**Participants:** pregnant adolescents<br><br>**Setting:** Obstetric setting<br><br>**Dates of data collection:** 2018	**Primary Findings:**<br>• A strategy of referral to counselling interventions was cost effective in the theoretical cohort, with 8,935 fewer cases of perinatal depression, 1,606 fewer cases of chronic depression, 166 fewer preterm deliveries, 4 fewer neonatal deaths, 1 fewer case of cerebral palsy, 20 fewer cases of SIDS. In total, there were 21,976 additional QALYs and cost savings of $223,549,872, making it the dominant strategy (better outcomes with lower costs).<br>• Counselling interventions remained cost saving until the annual direct and indirect cost of chronic, severe depression was set below $30,000, at which point it became cost effective (baseline input: $182,309).<br>• It is cost effective to refer all pregnant adolescents for preventive counselling interventions.	Using a theoretical cohort, Franta et al. (2022) found that counselling was a cost-effective preventative measure, leading to fewer cases of perinatal and chronic depression
Moore Simas et al (2020)<br><br>(Moore Simas et al., 2020)<br><br>USA	**Study Design:** Cohort study – economic evidence<br><br>**Type of intervention [*exposure*]:** PND.<br><br>**Data collection methods**: Administrative claims data from the IBM Watson Health MarketScan Databases	**Sample size:** 135,678<br><br>**Participants:** mother-child pairs with and without postpartum depression (PND) exposure<br><br>**Setting:** USA healthcare setting.<br><br>**Dates of data collection:** 2010 to 2016	**Primary Findings:**<br>• 33,314 mother-child pairs with PND exposure were propensity score matched to 102,364 mother-child pairs without PND exposure.<br>• During the 24-month follow-up period, HRU across most service categories was significantly higher among children in the PND exposure cohort than non-PND exposure cohort.<br>• Among outpatient services, the percentages of children with a physician specialist service (68% versus 64%), early-intervention screening (40% versus 37%), and an emergency room visit (48% versus 42%)	This cohort study assessed healthcare resource utilization (HRU) and costs in children of mothers with and without PND

For peer review only

			were greater in children of mothers with PND (all $p < .001$).<br>• Furthermore, children of mothers with PND incurred 12% higher total healthcare costs in the first 24 months of life compared to children of mothers without PND ($24,572 versus $21,946; $p < .001$).<br>• After excluding mothers with preterm delivery, the proportion of children with ER visits, physician specialist services, and outpatient pharmacy claims was significantly higher in the PND exposure cohort than non-PND exposure cohort (all $p < .001$).<br>**Additional Findings:**<br>The results of this analysis suggest that HRU and costs over the first 24 months of life in children of mothers with PND exceeded that of children of mothers without evidence of PND.	
Petrou et al (2002)<br><br>(Petrou et al., 2002)<br><br>**UK**	**Study Design**: Economic evaluation in which unit costs were applied to resource-use data collected alongside a longitudinal study of women at high risk of developing PND. Unit costs were applied to estimates of health and social care resource use made by 206 women recruited from antenatal clinics and their infants. Net costs per mother-infant dyad over the first 18 months post-partum were estimated.<br><br>**Type of intervention [*exposure*]:** Preventative PND intervention.<br><br>**Data collection methods**: primiparous women attending antenatal clinics at 26–28 weeks of gestation were screened using a predictive index for PND. Women identified as being at high risk of developing PND were entered into an RCT of a preventive intervention for PND delivered by trained health visitors. Economic data of women in the trial and in the observational study were pooled. An independent researcher assessed the mental state of all women at 8 weeks, 18 weeks, 12 months and 18 months post-partum using the	**Sample size:** 206<br><br>**Participants:** Primiparous women at high risk of developing PND<br><br>**Setting:** antenatal clinics<br><br>**Dates of data collection:** May 1997 to April 1999	**Primary Findings:**<br>Mean mother-infant dyad costs were estimated at £2,419.00 for women with PND and £2026.90 for women without PND, a mean cost difference of £392.10 (P=0.17). The mean cost differences between women with and without PND reached statistical significance for community care services (P=0.01), but not for other categories of service. Economic costs were higher for women with extended experiences of the condition.	Aimed to estimate the economic costs of PND in a geographically defined cohort of women at high risk of developing the condition.

For peer review only

	Structured Clinical Interview for DSM–III–R diagnoses (SCID–II).			
Petrou et al (2006)<br><br>(Petrou et al., 2006)<br><br>**UK**	**Study Design** A prospective economic evaluation was conducted alongside a pragmatic RCT<br><br>**Type of intervention [*exposure*]:** psychosocial and psychological interventions including counselling for the prevention of PND.<br><br>**Data collection methods:** Data on health and social care use by women and their infants up to 18 months postpartum were collected, using a combination of prospective diaries and face-to-face interviews	**Sample size:** 151 women<br><br>**Participants:** Women considered at high risk of developing PND were allocated randomly to the preventive intervention (*n* = 74) or to routine primary care (*n* = 77)<br><br>**Setting:** Health care setting.<br><br>**Dates of data collection:** c.2000	**Primary Findings:**<br>• Women in the preventive intervention group were depressed for an average of 2.21 months (9.57 weeks) during the study period, whereas women in the routine primary care group were depressed for an average of 2.70 months (11.71 weeks).<br>• The mean health and social care costs were estimated at £2,396.9 per mother–infant dyad in the preventive intervention group and £2,277.5 per mother–infant dyad in the routine primary care group, providing a mean cost difference of £119.5 (bootstrap 95 percent confidence interval [CI], −535.4, 784.9).<br>• At a willingness to pay threshold of £1,000 per month of PND avoided, the probability that the preventive intervention is cost-effective is .71 and the mean net benefit is £383.4 (bootstrap 95 percent CI, −£863.3–£1,581.5).<br><br>**Additional Findings:**<br>The preventive intervention is likely to be cost-effective even at relatively low willingness to pay thresholds for preventing 1 month of PND during the first 18 months postpartum. Given the negative impact of PND on later child development.	This cost-effectiveness analysis found that given the negative impact of PND on later child development, preventive interventions are likely to be cost-effective even at relatively low willingness to pay thresholds for preventing one month of PND during the first 18 months post-partum.
Roberts et al (2001) [41]<br><br>**Canada**	**Study design:** Cross-sectional economic evaluation<br><br>**Data collection methods:** EPDS and the Health and Social Service Utilization Questionnaire (HSUQ)	**Sample size:** 1,250<br><br>**Participants:** mothers of infants.<br><br>**Setting:** Canadian healthcare setting<br><br>**Dates of data collection:** 1999	**Primary findings:**<br>Costs were notably different for mothers with and without depression as determined by the EPDS (score of > 12). The total cost for health and social care $845 for mothers with depression and their infant's vs $413 for those with lower scores. This was statistically significant difference at the (p < .01).<br>**Additional findings:**<br>Costs for social work visits were higher for mothers with depression and mothers with low incomes.	A cross-sectional study of 1250 mothers of infants in a Canadian setting used the EPDS to investigate the costs associated with perinatal depression. It was found that<br>costs were notably different for mothers with and without depression. The total cost for health and social care was $845 for mothers with depression and their infant's vs $413 for those with lower

			Total health and social care costs were double for mothers with family income below $20,000 ($788 v $399) and for mothers with clinical depression ($845 v $413). Nursing care costs were greater for mothers with high depression scores ($135 v $81).	depression scores. This was statistically significant different at p < .01.
Stevenson et al (2010) (Stevenson et al., 2010)<br><br>UK	**Study Design:** cost-effectiveness analysis to assess group-CBT (gCBT) in comparison with routine primary care for women with PND in the UK.<br><br>**Type of intervention [*exposure*]:** Group-CBT<br><br>**Data collection methods:** SR	**Sample size:** 401<br><br>**Participants:** Data were analysed from 401 women with an EPDS score of 12 or greater at 6 weeks after childbirth, which had completed both the EPDS and the SF-6D questionnaire at both 6 weeks and 6 months<br><br>**Setting:** Postnatal healthcare setting in the UK<br><br>**Dates of data collection:** Pre July 2009 (when PONDER study was published**).**	**Primary Findings:**<br>The mean cost per QALY from the stochastic analysis was estimated to be £36,062; however, there was considerable uncertainty around this value. The EVPI was estimated to be greater than £64 million; the key uncertainties were in the cost per woman of providing treatment and in the statistical relationship between changes in EPDS values and changes in SF-6D values. The expected value of perfect partial information for both of these parameters was in excess of £25 million.<br><br>**Additional Findings:**<br>The use of gCBT does not appear to be cost-effective; however, this decision is uncertain. The value of information analyses conducted indicates that further research to provide robust information on key parameters is needed and appears justified in cost-effective terms.	This economic evaluation found that gCBT does not appear to be cost-effective due to the lack of literature providing robust information. Only one study, an RCT, was deemed applicable to the decision problem.
Wilkinson et al (2017)<br><br>(Wilkinson et al., 2017)<br><br>**USA**	**Study Design:** Modelling study<br><br>**Type of intervention [*exposure*]:** N/A<br><br>**Data collection methods**: Hypothetical cohort	**Sample size:** 1,000<br><br>**Participants:** follows a hypothetical cohort of 1000 pregnant women experiencing one live birth over a 2-year time horizon.<br><br>**Setting:** USA healthcare setting.<br><br>**Dates of data collection:** data were obtained from literature published between 1995 and 2015.	**Primary Findings:**<br>• Screening for and treating postpartum depression and psychosis produced 29 more healthy women at a cost of $943 per woman.<br>• The incremental cost-effectiveness ratios of the intervention branch compared to usual care were $13,857 per QALY gained (below the commonly accepted willingness to pay threshold of $50,000/QALY gained) and $10,182 per remission achieved.<br>• These results were robust in both the deterministic and probabilistic sensitivity analyses of input parameters.<br><br>**Additional Findings:**	This economic modelling study modelled the cost-effectiveness of physicians screening for and treating postpartum depression and psychosis in partnership with a psychiatrist.

			Screening for and treating postpartum depression is a cost-effective intervention and should be considered as part of usual postnatal care, which aligns with the recently proposed recommendations from the U.S. Preventive Services Task Force.	

Table 6: Data extraction table for Randomised Controlled Trials on maternal depression

**Citation (Country)**	**Study Details**	**Participants and setting**	**Key findings**	**Observations**
Grote et al (2017)<br><br>Grote et al., 2017)<br><br>**USA**	**Study Design:** RCT with blinded assessment<br><br>**Type of intervention [*exposure*]:** 18 months **MOMCare** collaborative care depression intervention (choice of brief interpersonal psychotherapy or pharmacotherapy or both) with enhanced maternity support services (MSS-Plus).<br><br>**Data collection methods:** Blinded telephone assessments, including depression severity on SCL-20. Unit costs of MOMCare intervention actual salary rate + fringe benefits + 30% overheads	**Sample size:** 152<br><br>**Participants:** 152 pregnant women 12-32 wks gestation with probable major depression or dysthymia (PTSD). Plus 12 excluded from analysis due to missing final data.<br><br>**Setting:** 10 county public health centres<br><br>**Dates of data collection:** Recruited Jan 2010 – July 2012. Study ended 2014	**Primary Findings:** when controlled for baseline depression severity, women with probable depression and PTSD in MOMCare had 68 more depression-free days over 18 months than those in MSS-Plus (p,.05). Additional $1,312. depression care cost per MOMCare participant with comorbid PTSD. Incremental net benefit of MOMCare was positive if a depression free days was valued at ≥ $20<br><br>**Additional Findings:**<br>Unit costs used 2013:<br>$80 per 45-50 min depression care specialist (DCS) visit<br>$31 per 20-30 min DCS phone call<br>(Both included time for outreach efforts and record keeping)<br>$247 fixed cost per patient for caseload supervision and info support<br>Other references to US-based data sources	In this RCT a multicomponent collaborative care intervention for depression (MOMcare - a choice of brief interpersonal psychotherapy or pharmacotherapy or both) with enhanced maternity support services (MSS-Plus) in the public health system of Seattle, USA. The incremental benefit and cost and the net benefit for women with major depression and PTSD was estimated. When controlled for baseline depression severity, women with probable depression and PTSD in MOMCare had 68 more depression-free days over 18 months than those in MSS-Plus (p<.05). There was an additional £1,943▪▪ depression care cost per MOMCare participant with comorbid PTSD. The incremental net benefit of MOMCare was positive if depression free days was valued below £18▪▪. For women with probable major depression and PTSD, MOMCare had a significant clinical benefit over MSS-Plus, with only a moderate increase in health services cost.[1]
Henderson et al (2019)	**Study Design:** PONDER Cluster RCT	**Sample size: From** 101 GP practices, 4,084 participants consented, baseline data from 3,449 participants.	**Primary Findings:** 99% probability of cost effectiveness at £20,000 at 6 months postnatal	This study found that CBT had a marginally higher probability of being cost-effective than a person-centred approach.

1 ▪▪ Prices have been inflated and converted to GBP [52].

For peer review only

For peer review only - http://bmjopen.bmj.com/site/about/guidelines.xhtml

(Henderson et al., 2019)<br><br>**UK**	**Type of intervention [*exposure*]:** GP practices assigned to usual health visitor (HV) care, HV trained to assess for PND plus offering either a CBA or a person-centred approach (PCA) weekly for 8 weeks<br><br>**Data collection methods:** Postal questionnaires: Baseline incl EPDS and SF36 at 6 weeks, Postnatal questionnaires at 6, 12 and 18 months postnatal. Resource use logs were completed by HVs based on their and GP records	**Participants:** 2,241 lower risk women completed EPDS at 6 months – 767 control, 1,474 intervention. 1,459 women provided economic data.<br><br>**Setting:** GP practices<br><br>**Dates of data collection:** April 2003 for 3 years	Compared with controls, adjusted 6 months costs were £82 lower with the interventions<br><br>**Additional Findings:**<br>Little difference CBA to PCA – CBA marginally higher probability of being cost effective.	

Table 7: Data extraction table for Systematic Reviews on maternal depression

Citation (Country)	Study Details	Participants and setting	Key findings	Observations
Gurung et al (2018)<br><br>[37]<br><br>**UK**	**Study Design:** SR of economic evaluations<br><br>**Type of intervention [*exposure*]:** PND<br><br>**Data collection methods:** Search strategy across eight electronic databases and other sources.	**Sample size:** 17 economic evaluations met the criteria for this SR.<br><br>**Setting:** UK maternal healthcare setting.<br><br>**Dates of data collection:** papers found from 2000-2015	**Primary Findings:**<br>• Three studies found that a combination of PND screening and treatment was cost-effective.<br>• Three studies reported that treatments such as psychological therapy, facilitated self-help and customized treatment were more cost-effective than standard care<br>• Four studies found positive results for preventive strategies which involved peer support or counselling and other specific support<br>• Group CBT was not found to be cost-effective compared to standard care in one study.<br><br>**Additional Findings:** Study aimed to identify interventions to prevent or treat PND for which an economic evaluation had been conducted and to	This SR found positive results for preventive strategies which involved peer support or counselling and other specific support. However, group cognitive behavioural therapy (CBT) was not found to be cost-effective compared to standard care in one study.

			evaluate the health and non-health outcomes included.	
Morrell et al (2016)<br><br>(Morrell et al., 2016)<br><br>UK	**Study Design:** SR, evidence synthesis and meta-analysis.<br><br>**Type of intervention [*exposure*]:** person-centred approach (PCA)-based and CBT, interpersonal psychotherapy (IPT) and education on preparing for parenting, promoting parent–infant interaction, peer support, the involvement of partners and access to several visits from a midwife or health visitor trained in person-centred or cognitive–behavioural approaches.<br><br>**Data collection methods**: MEDLINE, EMBASE, Science Citation Index and other databases.	**Sample size:** 122 studies met the inclusion criteria for this SR<br><br>**Participants:** postnatal women, their infants, and their families.<br><br>**Setting:** UK maternal healthcare setting.<br><br>**Dates of data collection:** December 2012 to July 2013.	**Primary Findings:**<br>• The most beneficial interventions appeared to be midwifery redesigned postnatal care [as shown by the mean 12-month EPDS score difference of -1.43 (95% credible interval -4.00 to 1.36)], person-centred approach (PCA)-based and cognitive-behavioural therapy (CBT) interpersonal psychotherapy (IPT) and education on preparing for parenting, promoting parent-infant interaction, peer support, IPT and PCA and CBT.<br>• Women valued seeing the same health worker, the involvement of partners and access to several visits from a midwife or health visitor trained in person-centred or cognitive-behavioural approaches.<br>• The most cost-effective interventions were estimated to be midwifery redesigned postnatal care, PCA and IPT-based intervention.<br>• Expected value of partial perfect information (EVPPI) for efficacy data was in excess of £150M for each population. Given the EVPPI values, future trials assessing the relative efficacies of promising interventions appears to represent value for money.	This SR with meta-analysis found that the most beneficial and cost-effective interventions appeared to be midwifery redesigned postnatal care, person-centred approach (PCA) and interpersonal psychotherapy (IPT). Women valued seeing the same health worker, partners' involvement, and access to several visits from a midwife or health visitor trained in person-centred or cognitive-behavioural approaches

For peer review only

Table 8: Data extraction table for studies including maternal health and wellbeing

Citation (Country)	Study Details	Participants and setting	Key findings	Observations

Chojenta et al (2019)<br><br>(Chojenta et al., 2019)<br><br>Australia	**Study design:** Cross- sectional<br><br>**Data collection methods:** Health economics modelling study.<br><br>Data were taken from the Australian Longitudinal Study on Women's Health (ALSWH), an ongoing population-based study of health and well-being.	**Sample size:12,689**<br><br>**Participants:** Three cohorts of women born 1973–78, 1946–1951 and 1921–1926, with a fourth cohort born in 1989–1995 added in 2012.<br><br>**Setting:** Australian healthcare setting.<br><br>**Dates of data collection:** 1921 to 1995	**Primary findings:**<br>The healthcare costs for postnatal women who had poor mental health prior to birth was $1,792 (AUSD). This is on average 11% more than for mothers with no previous history of poor mental health.	This modelling study from Australia, utilising cohort data from 1921 to 1995 found that the healthcare costs for postnatal women who had poor mental health prior to birth was $1,792 (AUSD). This is on average 11% more than for mothers with no previous history of poor mental health.
Moran et al (2020)<br><br>(Moran et al., 2020)<br><br>Ireland	**Study Design:** SR<br><br>**Type of intervention [*exposure*]:**<br>SR with the aim to estimate the economic burden of common health problems associated with pregnancy and childbirth, such as incontinence, mental health problems, or gestational diabetes, excluding acute complications of labour or birth, or severe acute adverse maternal outcomes.<br><br>**Data collection methods**: Searches of Medline, Embase, CINAHL, PsycINFO and EconLit databases.	**Sample size:** 38 studies met the inclusion criteria for this SR.<br><br>**Participants:** pregnant women and women who have given birth<br><br>**Setting:** Irish healthcare setting.<br><br>**Dates of data collection:** Up to November 2019	**Primary Findings:**<br>Thirty-eight relevant studies were identified, some of which reported incremental costs for more than one health problem (16 gestational diabetes, 13 overweight/obesity, 8 mental health, 4 hypertensive disorders, 2 nausea and vomiting, 2 epilepsy, 1 intimate partner violence). A high level of heterogeneity was observed in both the methods used, and the incremental cost estimates obtained for each morbidity. Average incremental costs tended to be higher in studies that modelled a hypothetical cohort of women using data from a range of sources (compared to analyses of primary data), and in studies set in the United States. No studies that examined the economic burden of some common pregnancy-related morbidities, such as incontinence, pelvic girdle pain, or sexual health problems, were identified.<br><br>**Additional Findings:**<br>Our findings indicate that maternal morbidity is associated with significant costs to health systems and society, but large gaps remain in the evidence base for the economic burden of some common health problems associated with pregnancy and childbirth. More research is needed to examine the economic burden of a range of common maternal health problems, and future research should adopt consistent methodological approaches to ensure comparability of results.	In this SR, among the four included studies that examined costs during pregnancy, birth, or the immediate post-partum period, the estimated incremental costs of poor maternal mental health ranged from £422 to £742.
Morrell et al (2000)	**Study Design:** RCT	**Sample size:** 623	**Primary Findings:** 551 completed 6 weeks questionnaire, 493 at 6 months.	This study found that there were no savings to the NHS

For peer review only

[44]<br><br>**UK**	**Type of intervention [*exposure*]:**<br>Up to 10 home visits in the first postnatal month of up to three hours duration by a community postnatal support worker.<br><br>Impact of community postnatal support worker in addition to usual community midwife care on rest and recovery, health status, satisfaction with services and NHS Resource use and costs.<br><br>**Data collection methods:** Postal questionnaires (including SF36 and EPDS).	**Participants:** Postnatal women delivering at a university hospital<br><br>**Setting:** Home and community<br><br>**Dates of data collection:** Recruitment on labour wards from October 1996 to November 1997	No evidence of use of fewer NHS services by women using the support worker versus controls at 6 weeks or 6 months.<br>Additional costs per woman at 6 weeks of £179.58 mostly due to support worker training (p<0.001).<br><br>**Additional Findings:** No diff primary outcome at 6 weeks but p<0.05 for physical and social functioning and p=005 EPDS for controls.<br>No difference in SF36 health status scores, EPDS scale or Duke Functional Social Support scale, rate of breastfeeding).	over six months after the introduction of a community support worker service and no improvement to the health status among the women in the intervention group, which was measured by an SF-36 questionnaire. At six weeks, the mean total NHS costs were £975□ for the intervention group and £700 for the control group. At six months, the figures were £1,250 and £980, respectively.
Ride (2018)<br><br>(Ride, 2018)<br><br>**UK**	**Study Design:** Modelling study (health economics)<br><br>**Data collection methods:** Decision analytic modelling	**Date of model:** 2018<br><br>The models were developed using TreeAge Pro 2015 software (TreeAge Software, Inc., Williamstown, MA, USA). The population of interest was postnatal women and their children in the United Kingdom, because much of the data came from that setting; this gave an explicit societal threshold of £20,000 to £30,000 per QALY for cost-effectiveness analysis in health care. A health sector perspective was taken, except for the children's model, which expanded to a public sector perspective to accommodate educational costs. A discount rate of 3.5% was applied to costs and QALYs, with discounting applied back to the child's birth. All costs were converted to 2014 pounds sterling.	**Primary Findings:** The results suggest that broader boundaries, particularly extension of the time horizon, could make substantial differences to estimated cost-effectiveness. Inclusion of family effects without extension of the time horizon had little impact, but where a longer time horizon was used, family effects could make a significant difference to the conclusions drawn from cost-effectiveness analysis<br><br>**Additional Findings:**<br>The authors note that it is important not only to consider caregiving but also family health effects in the outcomes of maternal health studies.	By ignoring broader sets of costs and outcomes, resources in postnatal mental health may be misallocated, and as a result, some women may not benefit as much from interventions that might be cost-effective given a broader time-horizon.

For peer review only

For peer review only

Search strategy

Below is an example of a search strategy for the Medline database.

ID	Search terms
1	exp Pregnancy/
2	(pregnan* or childbearing).ti,ab,kw.
3	(postpartum or post-partum or postnatal or postnatal or perinatal or peri-natal or prenatal or pre-natal or antenatal or ante-natal or matern*).ti,ab,kw.
4	perinatal anxiety.ti,ab,kw. or exp Perinatal anxiety/
5	exp Depression/
6	depress$.ti,ab,kw.
7	5 or 6
8	(Infant or baby or child).ti,ab,kw
9	(care* or treatment).tiab.kw
10	NHS.ti,ab,kw
11	hospitali$ation*.ti,ab,kw
12	exp Resource allocation/
13	economic evaluation$.ti,ab,kw.
14	(cost* or economic* or pharmacoeconomic*).ti.
15	13 or 14
16	exp "costs and cost analysis"/ or exp Health Care Costs/
17	exp Cost-Benefit Analysis/
18	(cost* adj2 (effective* or utility* or benefit* or consequence* or minimi*)).ti,ab,kw.
19	16 or 17 or 18
20	quality-adjusted life year$.ti,ab,kw. or exp Quality-Adjusted Life Years/
21	Or 7 and 15 and 19

For peer review only

Abbreviations

Abbreviation	Full	Aspect
ANOVA	Analysis of Variance	Analysis
ANRQ-R	Antenatal Risk Questionnaire	Tool
CATi	Computer Assisted Telephone Interviews	Research
CBA	Cognitive Behavioural Approach	Intervention
CBT	Cognitive Behavioural Therapy	Intervention
CEA	Cost Effectiveness Analysis	Analysis
CIDI	Composite International Diagnostic Interview	Research
CUA	Cost Utility Analysis	Analysis
DASS21	Depression, Anxiety and Stress Scale	Tool

DCS	Depression Care Specialist	Staff
DFD	Disease Free Day	Research
DSM-IV	Diagnostic and Statistical Manual for Mental Disorders 4th Edition	Source
eMBI	electronic Mindfulness-based Intervention	Intervention
EPDS	Edinburgh Postnatal Depression Scale	Tool
ePRO	electronic Patient Reported Outcomes	Research
EQ-5D-3L	EuroQol 5 Dimension 3 Level	Tool
GP	General Practitioner	Staff
gCBT	Group cognitive behavioural therapy	Intervention
HRU	Healthcare resource utilization	Analysis
HV	Health Visitor	Staff
ICD	International Classification of Diseases	Source
ICER	Incremental Cost-Effectiveness Ratio	Analysis
IG	Intervention Group	Research
IPT	Interpersonal psychotherapy	Intervention
ITT	Intention to Treat	Research
LGA	Local Government Area	Organisation
MBS	Medical Benefits Schedule	Source
MCH	Maternal and Child Health	Setting
MFAS	Maternal-Fetal Attachment Scale	Tool
MOMcare		*Study name*
MINI	Mini-International Neuropsychiatric Interview	Tool
NHS	National Health Service	Setting
OOP	Out of Pocket	Research
PAD	perinatal anxiety and/or depression	Diagnosis
PBS	Pharmaceutical Benefits Scheme	Source
PCA	Personalised Care Approach	Intervention
PHQ-9	Patient Health Questionnaire	Tool
PND	Postnatal depression	Diagnosis
PND	Post-partum depression	Diagnosis
PoNDER trial	POstNatal Depression Economic evaluation and Randomised trial	*Study name*
PRAQ-R	Pregnancy-Related Anxiety Questionnaire	Tool
PTSD	Post-Traumatic Stress Disorder	Diagnosis
QALY	Quality Adjusted Life Year	Analysis
RCT	Randomised controlled trial	Research
SCL-20	Hopkins Symptom Checklist-20	Tool
SF36	Short-Form 36	Tool
SIDS	Sudden infant death syndrome	Diagnosis
SPARCS	*Sleep, Parenting and Relationships in a Community Setting*	*Study name*
STAI	State-Trait Anxiety Questionnaire	Tool
TAU	Treatment as Usual	Research
TENS	Transcutaneous Electrical Nerve Stimulation	Intervention
WHO	World Health Organisation	Organisation
WWWT	What Were We Thinking	Tool

For peer review only

Quality appraisal of health economic evaluation studies (Drummond et al., 2015)

Drummond et al checklist 2015	**Petrou et al (2002)** (Petrou et al., 2002)	**Petrou et al (2006)** (Petrou et al., 2006)	**Ride et al (2016)** (Ride et al., 2016)	**Henderson et al (2019)** (Henderson et al., 2019)
1. Was a well defined question posed in an answerable form?	Yes	Yes	Yes	Yes
2. Was a comprehensive description of the competing alternatives given?	n/a	n/a	Yes	Yes
3. Was the effectiveness of the programs or services established?	n/a	n/a	Yes	Yes
4. Were all the important and relevant costs and consequences for each alternative identified?	n/a	n/a	Yes	Yes
5. Were costs and consequences measured accurately in appropriate physical units?	Yes	Yes	Yes	Yes
6. Were costs and consequences valued credibly?	Yes	Yes	Yes	Yes
7. Were costs and consequences adjusted for differential timing	n/a	n/a	No	No
8. Was an incremental analysis of costs and consequences of alternatives performed?	n/a	n/a	No	Yes
9. Was allowance made for uncertainty in the estimates of costs and consequences?	Yes	Yes	Yes	Yes
10. Did the presentation and discussion of study results include all issues of concern to users?	Yes	Yes	Yes	Yes

Source of checklist: Drummond, M. F., Sculpher, M. J., Claxton, K., Stoddart, G. L., & Torrance G W. (2015). *Methods for the economic evaluation of health care programmes* (4th ed.). Oxford: Oxford University Press.

For peer review only

Quality appraisal of health economic modelling studies with CHARMS Checklist (Moons et al., 2014)

Domain	Key items	**Counts et al (2022) -** (Counts et al., 2022)	**Franta et al (2022) -** (Franta et al., 2022)	**Ride (2018) -** (Ride, 2018)	**Wilkinson et al (2017) -** (Wilkinson et al., 2017)	**Bauer et al (2015)** (Bauer et al, 2015)	**Stevenson et al, (2010)** (Stevenson et al., 2010)
**SOURCE OF DATA**	Source of data (e.g., cohort, case-control, randomized trial participants	p.3	p.2	p.575	p.3	p.52	p.581
**PARTICIPANTS**	Participant eligibility and recruitment method (e.g., consecutive participants, location,	p.3	p.2	p.575	p.3	p.52	p.581
	Participant description	p.3	p.2	p.575	p.3	p.52	p.581
	Details of treatments received, if	p.5	p.2	p.575	p.3	p.52	N/A
	Study dates	p.4	p.2	p.575	p.3	p.52	p.581
**OUTCOME(S) TO BE PREDICTED**	Definition and method for measurement of outcome	p.4	p.2	p.574	p.4	p.53	p.581-582
	Was the same outcome definition (and method for measurement) used in all	Yes p.5	p.2	p.574	p.4	p.53	p.581-582
	Type of outcome (e.g., single or combined	p.3	p.5	p.574	p.4	p.53	p.581
	Was the outcome assessed without knowledge of	No	No	No	No	No	p.581
	Were candidate predictors part of the outcome	No	No	No	No	No	p.581

For peer review only

	Time of outcome occurrence or summary of duration of follow-up	p.5	p.5	p.578	p.4	p.52	p.581
**CANDIDATE PREDICTORS (OR INDEX TESTS)**	Number and type of predictors (e.g., demographics, patient history, physical examination,	p.5	p.5	p.577	p.6	p.55	p.582
	Definition and method for measurement of candidate predictors	p.5	p.5	p.575	p.6	p.55	p.580-582
	Timing of predictor measurement (e.g., at patient presentation, at diagnosis,	p.5	p.5	p.577	p.6	p.55	p.581
	Were predictors assessed blinded for outcome, and for each other	No	No	No	No	No	p.582
	Handling of predictors in the modelling (e.g., continuous, linear, non-linear transformatio	Unclear	Unclear	Unclear	Unclear	p.52	p.582
**SAMPLE SIZE**	Number of participants and number of outcomes/ev	p.3	p.2	p.575	P.3	p.55	p.582
	Number of outcomes/events in relation to the number of candidate predictors (Events Per Variable)	p.5	p.3	p.577	p.20	p.57	p.582
**MISSING DATA**	Number of participants with any missing value	p.4	Unclear	Unclear	Unclear	Unclear	Unclear

For peer review only

	Number of participants with missing data for each predictor	Unclear	Unclear	Unclea r	Unclear	Unclea r	Unclear
	Handling of missing data (e.g., complete-case analysis, imputation, or	Unclear	Unclear	Unclea r	Unclear	Unclea r	Unclear
**MODEL DEVELOPMEN T**	Modelling method (e.g., logistic, survival, neural network, or	Simulate d cohort model	Simulat ed cohort model	Decisi on analyti c model	Simulat ed cohort model	Decisi on analyti c model	Mathemati cal model
	Modelling assumptions satisfied	See Appendix 1 in the supplem ent	p.5	p.577	p.4	p.53	p.580
	Method for selection of predictors **for inclusion** in multivariable modelling (e.g., all candidate predictors, pre-selection based on unadjusted association with the	Unclear	Unclear	p.577	p.4	p.53	p.581
	Method for selection of predictors **during multivariable modelling** (e.g., full model approach, backward or forward selection) and criteria used (e.g., p-value, Akaike Information Criterion)	Unclear	Unclear	Unclea r	Unclear	p.53	Unclear

	Shrinkage of predictor weights or regression coefficients (e.g., no shrinkage, [illegible]	Unclear	Unclear	Unclear	Unclear	Unclear	Unclear
**MODEL PERFORMANCE**	Calibration (calibration plot, calibration slope, Hosmer-Lemeshow test) and Discrimination (C-statistic, D-statistic, log-rank)	p.5	Unclear	Unclear	Unclear	Unclear	Unclear
	Classification measures (e.g., sensitivity, specificity, predictive values, net reclassification improvement) and whether a-priori cut [illegible]	See e-appendix 3	p.6	p.577	p.6	No	p.581
**MODEL EVALUATION**	Method used for testing model performance: development dataset only (random split of data, resampling methods e.g. bootstrap or cross-validation, none) or separate external validation (e.g. temporal, geographical [illegible]	See e-appendix 3	Unclear	Unclear	p.6	No	Unclear

For peer review only

	In case of poor validation, whether model was adjusted or updated (e.g., intercept recalibrated, predictor effects	Unclear	Unclear	Unclea r	Unclear	No	Unclear
RESULTS	Final and other multivariable models (e.g., basic, extended, simplified) presented, including predictor weights or regression coefficients, intercept, baseline survival, model performance measures (with	Unclear	Unclear	Unclea r	Unclear	No	No
	Any alternative presentation of the final prediction models, e.g., sum score, nomogram, score chart, predictions for specific risk subgroups	No	No	p.578	p.23	No	No
	Comparison of the distribution of predictors (including missing data) for development and validation	No	No	No	No	No	No

For peer review only

**INTERPRETATION AND DISCUSSION**	Interpretation of presented models (confirmatory, i.e., model useful for practice versus exploratory, i.e., more research	p.7	p.5	p.577	p.6	p.56	p.583
	Comparison with other studies, discussion of generalizability, strengths and limitations.	p.7	p.5	p.577	p.6	p.58	p.583

JBI critical appraisal checklist for Systematic Reviews and Research Syntheses (Aromataris et al., 2015)

Citation	Q1. Is the review question clearly and explicitly stated?	Q2. Were the inclusion criteria appropriate for the review question?	Q3. Was the search strategy appropriate?	Q4. Were the sources and resources used to search for studies adequate?	Q5. Were the criteria for appraising studies appropriate?	Q6. Was critical appraisal conducted by two or more reviewers independently?	Q7. Were there methods to minimize errors in data extraction?	Q8. Were the methods used to combine studies appropriate?	Q9. Was the likelihood of publication bias assessed?	Q10. Were recommendations for policy and/or practice supported by the reported data?	Q11. Were the specific directives for new research appropriate?
(Camacho & Shields, 2018)	Yes	Yes	Yes	Yes	Yes	Unclear	Yes	Yes	Yes	Yes	Yes
(Gurung et al., 2018)	Yes	Yes	Yes	Yes	Yes	Unclear	Yes	Unclear	Yes	Yes	Yes
(Moran et al., 2020)	Yes	Yes	Yes	Yes	Yes	Yes	Yes	Yes	Yes	Unclear	Yes
(Morrell et al., 2016)	Yes	Yes	Yes	Yes	Yes	Yes	Yes	Yes	Yes	Yes	Yes

JBI Critical appraisal checklist for randomized controlled trials (Tufanaru et al., 2020)

Citation	Q1. Was true randomization used for assignment of	Q2. Was allocation to treatment groups	Q3. Were treatment groups simila	Q4. Were participants blind to treatment	Q5. Were those delivering treatment blind	Q6. Were outcomes assessors blind to	Q7. Were treatment groups treated identica	Q8. Was follow up complete and if not,	Q9. Were participants analyzed in the groups	Q10. Were outcomes measured in the	Q11. Were outcomes measured in a	Q12. Was appropriate statistical analysi	Q13. Was the trial design appropriate, and any

	participants to treatment groups?	concealed?	r at the baseline?	assignment?	to treatment assignment?	treatment assignment?	lly other than the intervention of interest?	were differences between groups in terms of their follow up adequately described and analyzed?	to which they were randomized?	same way for treatment groups?	reliable way?	s used?	deviations from the standard RCT design (individual randomization, parallel groups) accounted for in the conduct and analysis of the trial?
(Grote et al., 2017)	Yes	Yes	Yes	Unclear	Yes	Unclear	Yes	Yes	No	Yes	Unclear	Yes	N/A
(Morrell et al., 2000)	Yes	Yes	Yes	Yes	Yes	Unclear	Yes	Yes	Yes	Yes	Yes	Yes	Yes

JBI Critical Appraisal Checklist for Cohort Studies (Moola et al., 2020)

**Citation**	Q1. Were the two groups similar and recruited from the same population?	Q2. Were the exposures measured similarly to assign people to both exposed and unexposed groups?	Q3. Was the exposure measured in a valid and reliable way?	Q4. Were confounding factors identified?	Q5. Were strategies to deal with confounding factors stated?	Q6. Were the groups/ participants free of the outcome at the start of the study (or at the moment of exposure)?	Q7. Were the outcomes measured in a valid and reliable way?	Q8. Was the follow up time reported and sufficient to be long enough for outcomes to occur?	Q9. Was follow up complete, and if not, were the reasons to loss to follow up described and explored?	Q10. Were strategies to address incomplete follow up utilized?	Q11. Was appropriate statistical analysis used?
(Moore Simas et al., 2020)	Yes	Yes	Yes	No	No	Yes	Yes	Yes	Unclear	N/A	Yes

JBI Critical Appraisal Checklist for Cross-sectional studies (Moola et al., 2020)

Citation	Q1. Were the criteria for inclusion in the sample clearly defined?	Q2. Were the study subjects and the setting described in detail?	Q3. Was the exposure measured in a valid and reliable way?	Q4. Were objective, standard criteria used for measurement of the condition?	Q5. Were confounding factors identified?	Q6. Were strategies to deal with confounding factors stated?	Q7. Were the outcomes measured in a valid and reliable way?	Q8. Was appropriate statistical analysis used?
Dagher et al., 2012	Yes	Yes	Yes	Yes	No	No	Yes	Yes

Chojenta et al., 2019	Yes	Yes	Yes	Yes	Yes	Yes	Yes	Unclear
Ammerman et al., 2016	Yes	Yes	Yes	Yes	Yes	Yes	Yes	Yes
Roberts et al., 2001	Yes	Yes	Yes	Yes	No	N/A	Yes	Yes

For peer review only

PRISMA 2020 Checklist

Section and Topic	Item #	Checklist item	Location where item is reported
**TITLE**			
Title	1	Identify the report as a systematic review.	Page 2
**ABSTRACT**			
Abstract	2	See the PRISMA 2020 for Abstracts checklist.	Figure 1
**INTRODUCTION**			
Rationale	3	Describe the rationale for the review in the context of existing knowledge.	Page 4
Objectives	4	Provide an explicit statement of the objective(s) or question(s) the review addresses.	Page 4
**METHODS**			
Eligibility criteria	5	Specify the inclusion and exclusion criteria for the review and how studies were grouped for the syntheses.	Page 5
Information sources	6	Specify all databases, registers, websites, organisations, reference lists and other sources searched or consulted to identify studies. Specify the date when each source was last searched or consulted.	Page 5
Search strategy	7	Present the full search strategies for all databases, registers and websites, including any filters and limits used.	Supplementary Material
Selection process	8	Specify the methods used to decide whether a study met the inclusion criteria of the review, including how many reviewers screened each record and each report retrieved, whether they worked independently, and if applicable, details of automation tools used in the process.	Page 6
Data collection process	9	Specify the methods used to collect data from reports, including how many reviewers collected data from each report, whether they worked independently, any processes for obtaining or confirming data from study investigators, and if applicable, details of automation tools used in the process.	Page 6
Data items	10a	List and define all outcomes for which data were sought. Specify whether all results that were compatible with each outcome domain in each study were sought (e.g. for all measures, time points, analyses), and if not, the methods used to decide which results to collect.	Page 6
	10b	List and define all other variables for which data were sought (e.g. participant and intervention characteristics, funding sources). Describe any assumptions made about any missing or unclear information.	Page 6
Study risk of bias assessment	11	Specify the methods used to assess risk of bias in the included studies, including details of the tool(s) used, how many reviewers assessed each study and whether they worked independently, and if applicable, details of automation tools used in the process.	Supplementary Material
Effect measures	12	Specify for each outcome the effect measure(s) (e.g. risk ratio, mean difference) used in the synthesis or presentation of results.	Page 8
Synthesis methods	13a	Describe the processes used to decide which studies were eligible for each synthesis (e.g. tabulating the study intervention characteristics and comparing against the planned groups for each synthesis (item #5)).	Page 6
	13b	Describe any methods required to prepare the data for presentation or synthesis, such as handling of missing summary statistics, or data conversions.	Page 6
	13c	Describe any methods used to tabulate or visually display results of individual studies and syntheses.	From Page 9
	13d	Describe any methods used to synthesize results and provide a rationale for the choice(s). If meta-analysis was performed, describe the model(s), method(s) to identify the presence and extent of statistical heterogeneity, and software package(s) used.	Page 8
	13e	Describe any methods used to explore possible causes of heterogeneity among study results (e.g. subgroup analysis, meta-regression).	N/A
	13f	Describe any sensitivity analyses conducted to assess robustness of the synthesized results.	N/A
Reporting bias assessment	14	Describe any methods used to assess risk of bias due to missing results in a synthesis (arising from reporting biases).	Supplementary Material
Certainty	15	Describe any methods used to assess certainty (or confidence) in the body of evidence for an outcome.	

For peer review only - http://bmjopen.bmj.com/site/about/guidelines.xhtml

PRISMA 2020 Checklist

Section and Topic	Item #	Checklist item	Location where item is reported
assessment			
**RESULTS**			
Study selection	16a	Describe the results of the search and selection process, from the number of records identified in the search to the number of studies included in the review, ideally using a flow diagram.	Page 8
	16b	Cite studies that might appear to meet the inclusion criteria, but which were excluded, and explain why they were excluded.	Page 8
Study characteristics	17	Cite each included study and present its characteristics.	Page 8
Risk of bias in studies	18	Present assessments of risk of bias for each included study.	Page 8
Results of individual studies	19	For all outcomes, present, for each study: (a) summary statistics for each group (where appropriate) and (b) an effect estimate and its precision (e.g. confidence/credible interval), ideally using structured tables or plots.	Page 8
Results of syntheses	20a	For each synthesis, briefly summarise the characteristics and risk of bias among contributing studies.	Page 8
	20b	Present results of all statistical syntheses conducted. If meta-analysis was done, present for each the summary estimate and its precision (e.g. confidence/credible interval) and measures of statistical heterogeneity. If comparing groups, describe the direction of the effect.	Page 8
	20c	Present results of all investigations of possible causes of heterogeneity among study results.	Page 8
	20d	Present results of all sensitivity analyses conducted to assess the robustness of the synthesized results.	N/A
Reporting biases	21	Present assessments of risk of bias due to missing results (arising from reporting biases) for each synthesis assessed.	Supplementary Material
Certainty of evidence	22	Present assessments of certainty (or confidence) in the body of evidence for each outcome assessed.	Supplementary Material
**DISCUSSION**			
Discussion	23a	Provide a general interpretation of the results in the context of other evidence.	Page 25
	23b	Discuss any limitations of the evidence included in the review.	Page 2
	23c	Discuss any limitations of the review processes used.	N/A
	23d	Discuss implications of the results for practice, policy, and future research.	Page 26
**OTHER INFORMATION**			
Registration and protocol	24a	Provide registration information for the review, including register name and registration number, or state that the review was not registered.	Page 5
	24b	Indicate where the review protocol can be accessed, or state that a protocol was not prepared.	Page 5
	24c	Describe and explain any amendments to information provided at registration or in the protocol.	N/A
Support	25	Describe sources of financial or non-financial support for the review, and the role of the funders or sponsors in the review.	Page 27
Competing interests	26	Declare any competing interests of review authors.	Page 27
Availability of data, code and other materials	27	Report which of the following are publicly available and where they can be found: template data collection forms; data extracted from included studies; data used for all analyses; analytic code; any other materials used in the review.	Page 9 onwards

For peer review only

PRISMA 2020 Checklist

From: Page MJ, McKenzie JE, Bossuyt PM, Boutron I, Hoffmann TC, Mulrow CD, et al. The PRISMA 2020 statement: an updated guideline for reporting systematic reviews. BMJ 2021;372:n71. doi: 10.1136/bmj.n71
For more information, visit: http://www.prisma-statement.org/

For peer review only

BMJ Open

Health economic evaluations of preventative care for perinatal anxiety and associated disorders: A rapid review

Journal:	*BMJ Open*
Manuscript ID	bmjopen-2022-068941.R1
Article Type:	Original research
Date Submitted by the Author:	04-Apr-2023
Complete List of Authors:	Pisavadia, Kalpa; Bangor University, School of Medical and Health Sciences<br>Spencer, Llinos; Bangor University, Centre for Health Economics and Medicine Evaluation<br>Tuersley, Lorna; Bangor University,<br>Coates, Rose ; City University of London,<br>Ayers, Susan; City University,<br>Edwards, Rhiannon; Bangor University, Centre for Health Economics & Medicines Evaluation
**Primary Subject Heading**:	Health economics
Secondary Subject Heading:	Health economics
Keywords:	HEALTH ECONOMICS, PREVENTIVE MEDICINE, MENTAL HEALTH, Postpartum Women < Postpartum Period

SCHOLARONE™
Manuscripts

I, the Submitting Author has the right to grant and does grant on behalf of all authors of the Work (as defined in the below author licence), an exclusive licence and/or a non-exclusive licence for contributions from authors who are: i) UK Crown employees; ii) where BMJ has agreed a CC-BY licence shall apply, and/or iii) in accordance with the terms applicable for US Federal Government officers or employees acting as part of their official duties; on a worldwide, perpetual, irrevocable, royalty-free basis to BMJ Publishing Group Ltd ("BMJ") its licensees and where the relevant Journal is co-owned by BMJ to the co-owners of the Journal, to publish the Work in this journal and any other BMJ products and to exploit all rights, as set out in our licence.

The Submitting Author accepts and understands that any supply made under these terms is made by BMJ to the Submitting Author unless you are acting as an employee on behalf of your employer or a postgraduate student of an affiliated institution which is paying any applicable article publishing charge ("APC") for Open Access articles. Where the Submitting Author wishes to make the Work available on an Open Access basis (and intends to pay the relevant APC), the terms of reuse of such Open Access shall be governed by a Creative Commons licence – details of these licences and which Creative Commons licence will apply to this Work are set out in our licence referred to above.

Other than as permitted in any relevant BMJ Author's Self Archiving Policies, I confirm this Work has not been accepted for publication elsewhere, is not being considered for publication elsewhere and does not duplicate material already published. I confirm all authors consent to publication of this Work and authorise the granting of this licence.

For peer review only

Health economic evaluations of preventative care for perinatal anxiety and associated disorders: A rapid review

Miss Kalpa Pisavadia Kalpa.pisavadia@bangor.ac.uk

Dr Llinos Haf Spencer L.spencer@bangor.ac.uk

Dr Lorna Tuersley Lorna.tuersley@gmail.com

Dr Rose Coates Rose.coates@city.ac.uk

Professor Susan Ayers Susan.ayers.1@city.ac.uk

Professor Rhiannon Tudor Edwards R.t.edwards@bangor.ac.uk

Corresponding author: Kalpa Pisavadia Research Project Support Officer, Centre for Health Economics and Medicines Evaluation (CHEME), Ardudwy Hall, Normal Site, Bangor University, Bangor, Gwynedd, Wales, UK, LL57 2PZ. Email: Kalpa.pisavadia@bangor.ac.uk

For peer review only

Abstract

Background: Perinatal mental health (PMH) problems affect one in five women and cost the United Kingdom (UK) £8.1 billion for every year of births, with 72% of this cost due to the long-term impact on the child.

Aim: The aim of this rapid review was to investigate the type of health economic evaluations of preventative care for perinatal anxiety and associated disorders carried out within the National Health Service (NHS) and similar healthcare systems.

Methodology: This study adopted a rapid review approach, using principles of the standard systematic review process to generate quality evidence. This methodology features a systematic database search, PRISMA diagram, screening of evidence, data extraction, critical appraisal, and narrative synthesis.

Results: Database searches yielded 3,212 results published between January 2000 and July 2022. Titles and abstracts were screened, and Seventeen studies were included. Of these seventeen included papers, there were cost-effectiveness studies (n=6), modelling studies (n=6), cost-benefit study (n=1), a cost analysis study (n=1) and cost of illness studies (n=4).

Discussion: The results indicate a lack of economic evaluation specifically for perinatal anxiety, with most studies focussing on postnatal depression (PND). Interventions to prevent postnatal mental health problems being cost-effective. Modelling studies have also been conducted, which suggest that treating PND with counselling would be cost-effective.

Conclusion: The costs of not intervening in maternal mental health outweigh the costs of preventative interventions. Preventative measures such as screening and counselling for maternal mental health are shown to be cost-effective interventions to improve outcomes for women and children.

Key words: preventative, life-course, perinatal anxiety, postnatal depression, cost of illness, cost-effectiveness, economic modelling.

Article summary

Strengths of the rapid review

- The strength of this rapid review is that it has highlighted costs associated with perinatal mental health interventions in a rigorous, novel way which will benefit the NIHR funded (Award number: NIHR133727) Map Alliance Project team with the economic evaluation for that study (currently in progress).

Limitations of the rapid review

- There is an absence of health economic studies describing the range of public sector costs and costs to individuals from Scotland and Wales in relation to perinatal anxiety.
- Although health economic studies are showing the benefits of investing in PND, there are no published UK-based RCTs investigating perinatal mental

For peer review only - http://bmjopen.bmj.com/site/about/guidelines.xhtml

health interventions, which include information on costs (RCT's is the most scientifically rigorous method of hypothesis testing available and is regarded as the gold standard trial for evaluating the effectiveness of interventions). This indicates an evidence gap.

Introduction

The perinatal period refers to pregnancy and the first 12 months after childbirth [1]. One in five women experienced mental health problems during this time, and the cost is estimated to be £8.1 billion for every year of births in the United Kingdom (UK) [2] (see supplementary file 1 for a list of abbreviations). Maternal mental health problems include postnatal depression (PND) (also known as Postpartum Depression (PPD) internationally), characterised by depressed mood and anxiety, feelings of inadequacy, and impaired infant bonding [3]. More severe maternal mental health issues, such as postpartum psychosis, can present with feelings of agitation, confusion, hallucinations, and delusions [4]. Crucially, suicide is the leading cause of maternal death in the perinatal period [5]. It is, thus, imperative that proactive planning and cost-effective preventative solutions are a public policy priority.

The Maternal Mental Health Alliance warns that the COVID-19 pandemic may lead to a potential increase in perinatal mental health (PMH) difficulties [6]. A recent scoping review on the impact of the COVID-19 pandemic on maternal and perinatal health found that during pregnancy, self-reported rates of clinically relevant anxiety and depressive symptoms were higher among pregnant women compared to pre-pandemic levels [7]. Women who experience non-health-related stressors such as marital, housing, and financial difficulties or live in economically deprived areas were already at higher risk of PMH issues prior to the pandemic [8]. The COVID-19 pandemic further exacerbated the risk of impaired mental health due to limited antenatal care, reduced family support, social distancing, and quarantine rules. These factors, in combination with anxieties surrounding the transmission of the COVID-19 disease, have been found to significantly impact maternal mental health [7].

Untreated maternal mental illness not only impacts mothers but also adversely impacts their children, significantly contributing to wider societal and National Health Service (NHS) costs. Of the total costs of perinatal mental health difficulties in the UK, 72% is due to the long-term impact on the child [2]. An economic evaluation of a South London cohort found that for each child exposed to maternal perinatal depression, public sector costs exceeded £3,030. Costs due to reduced earnings were £1,400 per child, and health-related quality of life loss was valued at £3,760 per child [9].

For peer review only - http://bmjopen.bmj.com/site/about/guidelines.xhtml

Public sector costs are likely to be significantly reduced by utilising a prevention strategy to reduce the incidence of poor maternal mental health [9]. Decreased maternal and infant bonding, reduced breastfeeding initiation rates and duration, low birth weight, and poorer child growth have been associated with PND [10]. The regression analyses from an Australian cohort study revealed that children of mothers experiencing sub-clinical and increasing and persistently high depressive symptoms were twice as likely to have emotional and behavioural difficulties than children of mothers reporting minimal symptoms [11]. Delayed or impaired cognitive, linguistic, physical, and psychological health development has been reported in infants and children with mothers with PND [10]. There is also a risk of intergenerational transmission of socio-economic disadvantage in which maternal mental illness impacts the child's quality of life by having a long-term adverse effect on education and employment prospects [9,12].

Despite the long-term risks of untreated maternal mental health issues, as of 2014 in the UK, only 30-50% of women with PMH problems were identified, and only 7% were referred to specialist care [2]. Most women with PMH problems did not access care [2]. This may have been particularly the case for women with mild to moderate PMH problems or less commonly recognised problems, such as anxiety, obsessive-compulsive disorder (OCD), or post-traumatic stress disorder (PTSD) [2]. Furthermore, access to care may also be limited by maternal time constraints and fears of being judged [13]. Web-based approaches for delivering interventions could be a promisingly cost-effective solution in supporting mothers in the perinatal period by widening access to care, which hospitals could adopt as postnatal care support. A recent cost-effectiveness study, within a randomised controlled trial (RCT), evaluated a web-based approach for delivering a psychoeducational intervention [14]. This web-based approach was not only cost-effective in supporting first-time mothers but also had the best improvements in self-efficacy, social support, and psychological well-being of women in Singapore.

The National Institute for Health and Care Excellence recommend postnatal care for up to eight weeks after birth [15]. Since 2015, it has been recommended that UK midwives carry out emotional well-being checks at antenatal check-ups and at each postnatal contact up to eight weeks after birth. Women should be asked about their emotional well-being, what family and social support they have and their usual coping strategies for dealing with day-to-day matters. In 2018, the National Collaborating Centre for Mental Health worked with NICE to develop the Perinatal Mental Health Care Pathway [16]. The guidance in that report follows a process agreed upon by NICE and sets out pathways to deliver a strategic transformation of perinatal mental health care. Psychological interventions, either alone or in conjunction with pharmacological treatment, are recommended for complex or severe mental health problems following referral to a specialist community perinatal mental health team [1].

For peer review only - http://bmjopen.bmj.com/site/about/guidelines.xhtml

Since 2015 there have been improvements to funding plans and commitments in the provision of more specialist Community Perinatal Mental Health Services across the UK. For example, in 2019, the Scottish Government revealed that £52 million would be spent on improving access to perinatal and infant mental health services, and from 2018 to 2020, the Welsh Government increased recurrent annual funding from £1.5 million to £2.5 million for specialist PMH services [6]. In England, the Government committed £365 million to provide specialist perinatal community services across the country, as announced by NHS England in April 2019 [15]. However, it is questionable whether there is sufficient funding for long-term plans and where the investment for the workforce across the UK will come from [6].

Four systematic reviews investigating the clinical and cost-effectiveness of interventions to prevent postnatal depression have been published over the last decade [17–20]. The interventions included cognitive–behavioural therapy (CBT) approaches, psychotherapy, educational approaches, and peer-support based interventions to improve outcomes for women with poor postnatal mental health. Some studies investigated economic costs, and some studies investigated the clinical effectiveness and cost-effectiveness of interventions to prevent poor postnatal mental health. Some interventions were neither clinically effective nor cost-effective.

Aim

The aim of this rapid review was to investigate the type of health economic evaluations of preventative care for perinatal anxiety and associated disorders carried out within the National Health Service (NHS) and similar healthcare systems. The full protocol for this rapid review is available from PROSPERO [21].

Methods

This study adopted a rapid review approach, utilising principles from the standard systematic review process to generate quality evidence in a shorter time frame. This methodology follows the minimum requirements for rapid reviews, featuring a systematic database search, PRISMA diagram [22] (see figure 1) screening of evidence, data extraction, critical appraisal, and narrative synthesis. This revised methodology is used by the Wales COVID-19 Evidence Centre [23–25]. Cost-effectiveness outcomes are reported according to The Professional Society for Health Economics and Outcomes Research (ISPOR) guidelines [26].

Patient and Public Involvement

None

Search Strategy

The key evidence sources of this rapid review included PubMed, Cumulative Index to Nursing and Allied Health Literature (CINAHL), Cochrane Library, Applied Social

For peer review only - http://bmjopen.bmj.com/site/about/guidelines.xhtml

Sciences Index and Abstracts (ASSIA), PsycINFO and MEDLINE. The search terms consisted of words related to perinatal anxiety and/or depression, health and psychiatric services and economic evaluation terms. The searches were conducted on 23 April 2022. Mendeley reference management software was used to manage study articles found and remove duplicates. See supplementary file 1 for the full search strategy.

The eligibility criteria for the review are presented in Table 1 and are based on the Population, Intervention, Comparison and Outcome (PICO) framework [27]. This consisted of peer-reviewed economic evaluations of perinatal anxiety and associated disorders such as PND and PTSD from Organisation for Economic Co-operation and Development (OECD) countries in English published after January 2000.

Table 1: Participants, Intervention/exposure, Comparator and Outcomes (PICO) framework

**Question**	
What is the cost of care for women experiencing perinatal anxiety and associated disorders?	
**Participants**	Pregnant women or perinatal women
**Intervention / exposure**	Perinatal anxiety and associated disorders
**Comparator**	No comparator
**Outcomes**	Costs of primary care and support services for women experiencing perinatal anxiety and associated disorders
**Study Considerations**	
Primary research, secondary research, grey literature, and preprints	
**Databases**	
PubMed, CINAHL, Cochrane Library, ASSIA, PsycINFO, and MEDLINE	

Selection of studies

One reviewer (KP) independently selected potentially eligible studies based on a screening of titles and abstracts. Two reviewers (LHS and KP) selected additional studies from existing systematic reviews. The full texts of selected studies were assessed for eligibility by three reviewers (KP and LHS, with mediation by LT) in the data extraction process.

For peer review only - http://bmjopen.bmj.com/site/about/guidelines.xhtml

Data extraction

Data extraction and study quality assessment were performed by three reviewers (KP, LHS, LT). Data was collected on country, study design, intervention type, data collection methods and dates, sample size, and type of participants (See supplementary file 2 for data extraction tables).

Quality assessment

The quality assessment was undertaken by two reviewers (LHS and KP), and four papers were checked by a third reviewer for quality assurance purposes (LT). The Drummond checklist [28] was used for the quality appraisal of health economic papers, and the checklist for critical appraisal and data extraction for systematic reviews of prediction modelling studies (CHARMS) checklist was used for the modelling studies [29]. The Joanna Briggs Institute (JBI) critical appraisal tools were used for the quality appraisal, randomised clinical trials, cohort studies and cross-sectional studies [30–32] (see supplementary file 1).

[Insert figure 1 here]

For peer review only

Results

Searches of databases yielded 3212 results, of which 1226 duplicates were removed. The remaining 1986 results were screened against titles and abstracts, and an additional four papers were retrieved from existing systematic reviews. A total of 17 papers met the criteria for full-text screening. Eleven papers were excluded due to not being able to access the full text (n=4), ineligible study design (n=5), or lack of relevancy (non-OECD country) (n=2). Seventeen studies were included in this rapid review (see Figure 1 and Table 2).

Of these seventeen included papers, there were cost-effectiveness studies (n=5), modelling studies (n=6), cost-benefit study (n=1), a cost analysis study (n=1) and cost of illness studies (n=4). All included studies were peer-reviewed. The included studies were categorised according to main intervention: children, prevention, cost of maternal health, cost of single interventions, and comparison cost of interventions. The following discussion provides a more detailed overview of the findings.

Table 2: Map of maternal cost of illness studies by evidence type (including studies on depression, anxiety and maternal health and well-being)

**Type of Evidence**	**Type of intervention**					
	**Children**	**Prevention**	**Cost of maternal health**	**Cost of single interventions**	**Comparison cost of interventions**	**Number of studies**
**Cost-effectiveness**		Petrou et al. (2006) [3]		Morrell et al. (2000) [33]	Henderson et al. (2019) [34]	**5**
		Ride et al. (2016) [35]		Stevenson et al. (2010) [36]		
**Cost-benefit**					Grote et al. (2017) [37]	**1**
**Cost-analysis**	Moore Simas et al. (2020) [10]					**1**
**Cost-of-illness**			Petrou et al. (2002) [38]			**4**
			Dagher et al. (2012) [39]			
			Ammerman et al. (2016) [40]			
			Roberts et al, (2001) [41]			
**Economic modelling studies**	Bauer et al. (2015) [9]	Counts et al. (2022) [42,43]	Franta et al. (2022) [43]			**6**
	Ride (2018) [44]	Wilkinson et al. (2017) [4]	Chojenta et al. (2019) [45]			
**Total number of studies**	**3**	**4**	**6**	**2**	**2**	**17**

For peer review only - http://bmjopen.bmj.com/site/about/guidelines.xhtml

Table 3: Methodological considerations and cost-effectiveness results

Lead author (Year)	Intervention	Perspective (reasons)	Time horizon used in economic evaluation (reasons)	Discounting	Key cost-effectiveness results
Henderson et al (2019) [34]	**Intervention group:** PoNDER: Health visitor (HV) training to assess postnatal depression (PND) and deliver psychological approaches to women at risk of depression. **Control group:** Usual care	NHS and social care perspective.	Resource use data from 6 weeks to 6 months were collected on a resource use log completed by HVs based on their own and GP records	No discounting was necessary due to the duration of the follow-up period.	Costs and outcomes data were available for 1459 participants. 6-month adjusted costs were £82 lower in intervention than control groups, with 0.002 additional QALY gained. The probability of cost-effectiveness at £20,000 was very high (99%).
Morrell et al (2000) [33]	**Intervention group:** up to 10 home visits in the first postnatal month of up to three hours duration by a community postnatal support worker. **Control group:** Usual care	NHS perspective	Up to 10 home visits in the first postnatal month of up to three hours duration by a community postnatal support worker, and a 6-month follow-up.	No	Cost data showed that at six weeks the mean total NHS costs were £635 for the intervention group and £456 for the control group (P = 0.001). At six months figures were £815 and £639 (P = 0.001).<br><br>However, due to there being no differences between the groups in use of social services or personal costs, no cost-effectiveness analysis was conducted.
Petrou et al (2006) [3]	**Intervention group:** counselling and specific support for the mother relationship, targeted at women at high risk of developing postnatal depression. **Control group:** Usual care	The economic evaluation was conducted from a public sector perspective.	The time horizon for the economic evaluation mirrored the time horizon for the randomized controlled trial, namely the period between randomization and 18 months postpartum.	Various discounting rates were applied as necessary: 0 percent, 1.5 percent, 3 percent, 6 percent, and 10 percent.	The mean health and social care costs were estimated at £2,396.9 per mother-infant dyad in the preventive intervention group and £2,277.5 per mother-infant dyad in the routine primary care group, providing a mean cost difference of £119.5 (bootstrap 95 percent confidence interval [CI], -535.4, 784.9). At a willingness to pay threshold of £1,000 per month of postnatal depression avoided, the probability that the preventive intervention is cost-effective is.71 and the mean net benefit is £383.4 (bootstrap 95 percent CI, -£863.3-£1,581.5).
Ride et al (2016) [35]	Intervention group: What Were We Thinking (WWWT) - a psychoeducational intervention targeted at the partner	A range of perspectives including patient, NHS, and social services.	The time horizon of 6 months mirrored the trial follow-up period. No	No discounting was necessary due to the duration of the follow-up period.	The incremental cost-effectiveness ratios were $A36 451 per QALY gained and $A152 per percentage point reduction in 30-

For peer review only - http://bmjopen.bmj.com/site/about/guidelines.xhtml

	relationship, management of infant behaviour and parental fatigue. **Control group:** Usual care				day prevalence of depression, anxiety, and adjustment disorders. The estimate lies under the unofficial cost-effectiveness threshold of $A55 000 per QALY; however, there was considerable uncertainty surrounding the results, with a 55% probability that WWWT would be considered cost-effective at that threshold.
Stevenson et al (2010) [36]	**Intervention group:** Cognitive Behaviour Therapy (gCBT). **Control group:** Usual care	Health sector perspective	Treatment up to 8 weeks, and a 6-month follow-up.	No discounting was necessary due to the duration of the follow-up period.	The use of gCBT does not appear to be cost-effective.<br><br>The mean cost per quality adjusted life year (QALY) from the stochastic analysis was estimated to be £36,062; however, there was considerable uncertainty around this value. The expected value of perfect information (EVPI) was estimated to be greater than £64 million; the key uncertainties were in the cost per woman of providing treatment and in the statistical relationship between changes in the Edinburgh Postnatal Depression Scale (EPDS) values and changes in the Short Form – 6 Dimensions (SF-6D) values. The expected value of perfect partial information for both of these parameters was in excess of £25 million.

The included papers are organised under three different themes. The first theme is studies including perinatal anxiety, the second theme is perinatal depression, and the third theme is perinatal health and well-being. These included studies are detailed below, and all non-UK prices have been converted to pound sterling currency and inflated to the latest available prices [46–50].

For peer review only

For peer review only - http://bmjopen.bmj.com/site/about/guidelines.xhtml

Summary of studies including perinatal anxiety

This review found one study focussing on perinatal anxiety [17,35]. This study focusing on perinatal anxiety was an economic evaluation that consisted of a cost-effectiveness and cost-utility analysis of the What Were We Thinking (WWWT) intervention which was conducted alongside a cluster-randomised controlled trial [35]. WWWT is a psychoeducational intervention targeted at the partner relationship, management of infant behaviour and parental fatigue for the prevention of postnatal maternal mental health problems (See Table 3 for further details). There were no statistically significant differences in either costs or effectiveness. Limitations of the study included a short time-horizon (6 months), and there was no extrapolation beyond the time horizon as there were no significant differences in the period. Also, only costs for mothers and infants were collected, and costs and outcomes for partners were not, despite knowledge that health problems in couples tend to co-occur.

Summary of studies including perinatal depression

This review found fifteen studies focussing on perinatal depression [3,4,9,10,19,20,36–43,51]. A cross-sectional study from the USA conducted between 2006 and 2011 investigated the out-of-pocket expenses and insurer expenses of depressed mothers compared to non-depressed mothers [40]. Depressed mothers were more likely to incur insurer out-of-pocket expenses (£1,285 vs £853 □□) and have higher insurer expenses (£10,485 vs £7,508□□). The main limitation of this study was that the data was self-reported and therefore subject to recall bias, and as a result, the true medical costs associated with depression in high-risk mothers may be under-reported.

A study by [9] used the perspective of the public sector, individuals, and society to examine some of the outcomes and long-term economic implications experienced by offspring who have been exposed to perinatal depression in a South London cohort. Bauer et al. (2015) found that for each child exposed to perinatal depression, public sector costs exceeded £3,380□, costs due to reduced earnings were £1,562□, and health-related quality of life loss was valued at £3760□. A major limitation is that the model estimates were derived from small samples. Also, only mother, and infant costs were collected and costs from other individuals (such as other family members) were not included.

A decision analytic model used a simulated cohort of 1,000 Medicaid-enrolled pregnant individuals to evaluate the health care costs for individuals receiving PND preventive intervention or not, for 1 to 5 years post-partum [42]. This study found that providing preventive interventions for PPD resulted in an estimated 5-year saving of £602□□. The main limitation of this paper is that the model used a series of assumptions which may not be applicable to a particular group of individuals receiving specific PPD prevention interventions.

Dagher et al., (2012) conducted a cross-sectional study in the USA which investigated expenditure on healthcare services from hospital discharge until 11 weeks postpartum. There was a significant difference in healthcare expenditure between depressed and non-depressed women. The Edinburgh Postnatal Depression Scale (EPDS) was used to measure depression [52]. The total cost of all

For peer review only

mental health counselling visits for the depressed group (n=31) was £165□□, and the cost for the non-depressed group (n= 607) was £15.50□□ (in 2007). This was a statistically significant difference ($p < 0.001$). The main limitation was that the data was self-reported. Also, the cross-sectional nature of this study prohibited causal inferences.

Using a theoretical cohort of 180,000 individuals, a decision-analytic model using TreeAge Pro software was used to compare outcomes in pregnant adolescents who received versus did not receive counselling interventions [43]. This study found that it is cost-effective to refer all pregnant adolescents for preventive counselling interventions. Within the theoretical cohort for counselling, there were 8,935 fewer cases of PND, 1,606 fewer cases of chronic depression, 166 fewer preterm deliveries, four fewer neonatal deaths, 20 fewer cases of sudden infant death syndrome (SIDS), and one fewer case of cerebral palsy. In total, there were 21,976 additional QALYs and cost savings of £183,463,169⅄, making it the dominant strategy that had better outcomes with lower costs. The main limitation of this modelling study was that the model did not include the entire social and economic costs of infant death, which is a large contributing factor to perinatal depression.

An RCT trial compared a multicomponent collaborative care intervention for depression (MOMcare - a choice of brief interpersonal psychotherapy or pharmacotherapy or both) with enhanced maternity support services (MSS-Plus) in the public health system of Seattle, USA [37]. The incremental benefit and cost and the net benefit for women with major depression and PTSD were estimated. When controlled for baseline depression severity, women with probable depression and PTSD in MOMCare had 68 more depression-free days over 18 months than those in MSS-Plus ($p<.05$). There was an additional £1,943□□ depression care cost per MOMCare participant with comorbid PTSD. The incremental net benefit of MOMCare was positive if depression free days were valued below £18□□. For women with probable major depression and PTSD, MOMCare had a significant clinical benefit over MSS-Plus, with only a moderate increase in health services cost. The main limitation was that self-report measures were used to estimate the costs of mental health services, and these may have been over-estimated.

A cluster RCT of health visitors trained to assess PND and deliver psychological approaches to women at risk of depression plus either a cognitive behavioural approach or a person-centred approach weekly for eight weeks was conducted in 2019 [34]. A cost-effectiveness analysis was run parallel to this for all mothers at low risk of depression in accordance with the EPDS at six months postnatal. This study found that CBT had a marginally higher probability of being cost-effective than a person-centred approach. The main limitation was the short time horizon of 6 months postnatally which means that the risks of long-term adverse effects were not factored into the analysis.

A cohort study with a sample size of 135,678 mother-child pairs with and without PND exposure revealed similar findings [10]. The results of this analysis suggest that the health resource utilisation and costs over the first 24 months of life in children of mothers with PND exceeded that of children of mothers without evidence of PND £22,940□□ and £20,487□□, respectively. This was a significant difference of £2,453. A limitation of this study was that analysis was conducted on the commercially insured

For peer review only - http://bmjopen.bmj.com/site/about/guidelines.xhtml

population; thus, the results of this analysis may not be generalisable to PND patients with other or no insurance, likely representing persons of higher socio-economic status.

A longitudinal study (18 months) conducted in 2002 estimated the economic costs of PND in a geographically defined cohort of women at high-risk of developing the condition with the use of an RCT to identify women considered to be of high-risk [38]. Unit costs were applied to estimates of health and social care resource use made by 206 women and their infants recruited from antenatal clinics, and net costs per mother-infant dyad over the first 18 months post-partum were estimated. This study found that costs were £587□ higher for women with PND than for women without PND. Economic costs were also higher for women with extended experiences of the condition. Limitations of the study included the public sector approach that was taken. This did not allow measurement of non-medical costs such as travel and child-care costs.

A cost-effectiveness analysis of preventive interventions, which consisted of counselling and support for the mother–infant relationship, targeted at women at high-risk of developing PND, was conducted in 2006 [3]. This study found that given the negative impact of PND on later child development, preventive interventions are likely to be cost-effective even at relatively low willingness to pay thresholds for preventing one month of PND during the first 18 months post-partum. The mean health and social care costs were estimated at £3,345□ per mother–infant dyad in the preventive intervention group and £3,277□ per mother–infant dyad in the routine primary care group, providing a mean cost difference of £166□. The main limitations of this cost-effectiveness study were that the numbers in the intervention and control groups were relatively low (74 and 77 respectively). Also, the time horizon of 18 months was likely to underestimate the long-term effectiveness of the prevention intervention.

A cross-sectional study of 1,250 mothers of infants in a Canadian setting used the EPDS to investigate the costs associated with perinatal depression [41]. It was found that costs were notably different for mothers with and without depression. The total cost for health and social care was £833□□ for mothers with depression and their infants, compared to £406□□ for those with lower depression scores. This was statistically a significant difference at $p < .01$. The main limitation of this study was that only subjective measurements were used, which depended on phone calls to mothers about health and social service use in the past four weeks. The mothers may have overestimated their service use.

An economic evaluation conducted in 2010 compared the cost-effectiveness of group Cognitive Behavioural Therapy (gCBT) compared with routine primary care for women with PND in the UK [36]. This economic evaluation found that gCBT does not appear to be cost-effective due to the lack of literature providing robust information. Only one study, an RCT, was deemed applicable to the decision problem. However, there was no data available comparing gCBT with CBT which is a limitation of this study. Additionally, there is a possibility that the results are influenced by the therapist due to the small number of participants and clinicians.

For peer review only - http://bmjopen.bmj.com/site/about/guidelines.xhtml

A cost-effectiveness analysis found that screening for and treating post-partum depression is a cost-effective intervention and should be considered as a part of usual postnatal care [4]. This study followed a hypothetical cohort of 1,000 pregnant women experiencing one live birth over a 2-year time horizon. The analysis found that screening for and treating PND and psychosis produced 29 more healthy women at the cost of £938□□ per woman. The incremental cost-effectiveness ratios (ICERs) of the intervention branch compared to usual care were £13,702□□ per quality-adjusted life year (QALY) gained (below the commonly accepted willingness to pay threshold of $50,000/QALY gained) and $10,182 per remission achieved. The main limitation of this study is that due to the lack of data on other adverse events, this study only considered suicide ideation within its analysis. Therefore, there is a possibility that other adverse events may significantly decrease the cost-effectiveness of the intervention. Moreover, some adverse events may increase the cost-effectiveness of the intervention, for example, the long-term effects of untreated PND.

Summary of studies including maternal health and well-being

This review found four studies relating to the health and well-being of perinatal women [18,33,44,45]. An RCT conducted in 2000 aimed to establish the relative cost-effectiveness of postnatal support in the community in addition to the usual care provided by the community midwives [33]. Three hundred and eleven women were allocated to the intervention of up to ten home visits by a community postnatal support worker. The authors found no health benefit of additional home visits by community postnatal support workers compared with traditional community midwifery visiting, as measured by the Short Form 36 measure. At six months, there was no significant improvement in health status among the women in the intervention group despite there being a significant difference in costs of £1,250□ (intervention group) and £980□ (usual care group), (P = 0.001). Although there were no savings to the NHS over six months after the introduction of the community postnatal support worker service, the women in the intervention group were very satisfied with the support worker visits. A major limitation of the findings is that the time horizon was only six weeks, and wider public health implications were not explored.

Authors have suggested that prenatal interventions that do not seem cost-effective in the short term may be cost-effective over a longer time horizon [53]. Ride (2018) conducted a decision analytic modelling study and noted that it is important to consider caregiving and family health effects in the outcomes of maternal health studies. By not including broader sets of costs and outcomes, resources in postnatal mental health may be misallocated. As a result, some women may not benefit as much from interventions that might be cost-effective given a broader time horizon. Ride (2018) noted that the uncertainty surrounding the results in the decision analytic model may reflect decisions and investment in PND interventions.

A modelling study from Australia, published in 2019, utilised cohort data from 1921 to 1995 and found that the healthcare costs for postnatal women who had poor mental health prior to birth were £1,066˄ [45]. This is, on average, 11% more than for mothers with no previous history of poor mental health. These figures do not include out-of-pocket expenditure for the women who may have also purchased their own over-the-counter medications and had other patient expenses which were not captured in the analysis.

For peer review only - http://bmjopen.bmj.com/site/about/guidelines.xhtml

Discussion

The aim of this rapid review was to investigate the type of health economic evaluations of preventative care for perinatal anxiety and associated disorders carried out within the National Health Service (NHS) and similar healthcare systems. Twenty-one papers were included in this review from Australia, Canada, Ireland, the USA, and the UK, each examining maternal mental health.

The results indicate a lack of economic evaluation specifically for perinatal anxiety, with most study articles focusing on PND [36]. Only two included papers focussed on anxiety, with one being a systematic review looking at anxiety alongside depression [17]. The other was an economic evaluation of a maternal mental health intervention. Treatments for maternal mental health in the WWWT intervention consisted of health visitors with psychiatric training and group sessions focusing on parenting confidence and emotional well-being with online and face-to-face components [35]. The WWWT intervention shows promise as a preventive intervention. However, there is uncertainty as to its cost-effectiveness. However, there is uncertainty as to its cost-effectiveness. The analysis showed no statistically significant difference in costs or outcomes between the intervention and control groups, with the intervention estimated to cost £74.48 per participant.

Most of the studies included (n=15 of the 17 included studies) focussed on the cost of services and interventions for PND. The evidence suggests significant health resource costs outside of mental health services as well as social care costs for PND for mother and mother-infant dyad. Costs were significantly higher for children of mothers with PND than for children of mothers without PND. This was a statistically significant difference of £2,453 (p <.001) [10].

Significantly, counselling was found to be a cost-effective, preventative intervention for high-risk groups such as pregnant adolescents [43]. Using a hypothetical cohort, a found that counselling was a cost-effective preventative measure, leading to fewer cases of perinatal and chronic depression [43]. Another study estimated that group counselling (costing £114 per mother) cost around £73□ less than individual counselling (£187 per mother) for mothers with PND [42]. This study found that screening for PND costs less than £2 per mother [42]. Studies that combined screening for PND with an intervention were also found to be cost-effective, resulting in 29 more healthy women at a cost of £938□□ per woman [4]. The incremental cost-effectiveness ratios of the intervention branch compared to usual care were $13,857 per QALY gained (below the commonly accepted willingness to pay threshold of $50,000/QALY gained) and $10,182 per remission achieved.

Within this rapid review, the EPDS, a validated measure for postnatal depression and anxiety [52], was the most frequently used instrument to detect perinatal and PND in the included studies, followed by the SF-36 scale, postal questionnaires such as the Ontario health survey, Health and Social Service Utilisation Questionnaire (HSUQ), blinded telephone assessments and medical records, Medicaid data,

For peer review only

resource use logs completed by health visitors based on GP records, and prospective diaries and face-to-face interviews.

In summary, screening was found to be a relatively low-cost method of identifying women in need of mental health support during the perinatal period. Interventions to prevent postnatal mental health problems were found to be cost-effective [35]. Also, two modelling studies found that treating PND with counselling would be cost-effective [4,36].

Future research in this area should investigate how best to screen all mothers to prevent and treat further adverse outcomes such as anxiety, OCD, or PTSD [2]. Various psycho-social methods could be used to screen and provide treatment over the telephone, online or face-to-face. Interventions could be provided by a range of healthcare professionals, such as midwives, health visitors, counsellors, psychologists, and psychiatrists. The effectiveness and cost-effectiveness of each intervention, including screening, should be evaluated.

Web-based approaches are already promising to be cost-effective solutions to support mothers in the perinatal period. Most women of childbearing age, including women who reside in rural areas, now have access to the internet in the UK and similar health care systems. There is concern regarding web-based interventions. For example, the lack of engagement could lead to significant dropout [54]. Being able to access support and treatment using online resources has widened access to care to postnatal care support. A recent cost-effectiveness study alongside an RCT in Singapore evaluated a web-based approach for delivering a psychoeducational intervention [14]. This web-based approach was cost-effective in supporting first-time mothers and provided the best improvements in self-efficacy, social support, and psychological well-being of mothers in the perinatal period.

The MAP ALLIANCE study is funded by the NIHR (Award ID: NIHR133727) and is a UK based project which aims to examine the care offered and accessed by women experiencing perinatal anxiety and associated disorders. This study includes an economic component to evaluate the cost-of-service use for perinatal anxiety and associated disorders. It is anticipated that the MAP ALLIANCE study will lead to recommendations for accessible, integrated care acceptable to women. It will assist NHS commissioners and providers in designing and transforming services for perinatal women. This will increase the chances for women to receive better care to improve maternal and child outcomes [55].

Conclusion

This RR demonstrated that very few economic evaluations have focussed on perinatal anxiety, and those which reported on cost of perinatal depression had short time horizons which did not allow for long-term outcomes for the mother and child dyad to be addressed. However, there was some evidence that preventative measures, such as postnatal depression screening, combined with treatment, such as counselling for maternal mental health, are proven to be effective interventions to improve outcomes for women and children.

For peer review only - http://bmjopen.bmj.com/site/about/guidelines.xhtml

Recommendations

It is recommended that:

- Mothers should be screened for maternal mental health issues to identify mothers at risk and provide treatment, leading to better outcomes for the mother and child dyad.
- Studies focussing on interventions for perinatal anxiety as a distinct condition to other mental health issues such as depression should be conducted.
- Cost of intervention studies related to perinatal anxiety should be conducted.

Acknowledgements: The authors thank the wider MAP ALLIANCE team from City University of London and the University of Stirling for input into the development of this review. We would also like to thank Yasmine Noorani, Academic Support Librarian at Bangor University, for her assistance in creating our search strategy. Additional thanks to Dr Catherine Lawrence for early input and feedback on this paper.

Contributors: The review was conceived by RTE, and the protocol was developed by LT, KP and LHS; searches were undertaken by KP; article screening was carried out by KP and LHS with mediation by LT; quality appraisal was undertaken by KP, LHS and LT; data were interpreted by all authors; the manuscript was drafted by KP and LHS and critically reviewed by all authors, RC, SA and RTE.

Funding: This review is to complement the MAP ALLIANCE study, funded by the National Institute for Health and Care Research (NIHR) (Award ID: NIHR133727). This rapid review was partially funded by Health and Care Economics Cymru (HCEC), an organisation funded by Health and Care Research Wales.

Conflict of interest: All authors declare that they have no conflicts of interest.

Ethical approval: The MAP ALLIANCE study received ethical approval from the West of Scotland Research Ethics Committee on 6th May 2022 (REC 3. Reference: 22/WS/0029).

Data statement: No datasets were generated and/or analysed for this study

For peer review only - http://bmjopen.bmj.com/site/about/guidelines.xhtml

References

[1] NHS England and NHS Improvement. The Perinatal Mental Health Care Pathways NHS England and NHS Improvement. 2018.

[2] Bauer A, Personage M, Knapp M, Lemmi V, Adeleja B. Costs of perinatal mental health problems. London: 2014.

[3] Petrou S, Cooper P, Murray L, Davidson LL, Lawson C, Woolgar M, et al. Cost-effectiveness of a preventive counseling and support package for postnatal depression. Int J Technol Assess Health Care 2006;22:443–53. https://doi.org/10.1017/S0266462306051361.

[4] Wilkinson A, Anderson S, Wheeler SB. Screening for and Treating Postpartum Depression and Psychosis: A Cost-Effectiveness Analysis. Matern Child Health J 2017;21:903–14. https://doi.org/10.1007/s10995-016-2192-9.

[5] Howard LM, Flach C, Mehay A, Sharp D, Tylee A. The prevalence of suicidal ideation identified by the Edinburgh Postnatal Depression Scale in postpartum women in primary care: Findings from the RESPOND trial. BMC Pregnancy Childbirth 2011;11. https://doi.org/10.1186/1471-2393-11-57.

[6] Maternal Mental Health Alliance. Map of Specialist Community Perinatal Mental Health Teams (UK). 2020.

[7] Kotlar B, Gerson E, Petrillo S, Langer A, Tiemeier H. The impact of the COVID-19 pandemic on maternal and perinatal health: a scoping review. Reprod Health 2021;18. https://doi.org/10.1186/S12978-021-01070-6.

[8] Pilav S, Easter A, Silverio SA, de Backer K, Sundaresh S, Roberts S, et al. Experiences of Perinatal Mental Health Care among Minority Ethnic Women during the COVID-19 Pandemic in London: A Qualitative Study. Int J Environ Res Public Health 2022;19. https://doi.org/10.3390/IJERPH19041975.

[9] Bauer A, Pawlby S, Plant DT, King D, Pariante CM, Knapp M. Perinatal depression and child development: Exploring the economic consequences from a South London cohort. Psychol Med 2015;45:51–61. https://doi.org/10.1017/S0033291714001044.

[10] Moore Simas TA, Huang MY, Packnett ER, Zimmerman NM, Moynihan M, Eldar-Lissai A. Matched cohort study of healthcare resource utilization and costs in young children of mothers with postpartum depression in the United States. J Med Econ 2020;23:174–83. https://doi.org/10.1080/13696998.2019.1679157.

[11] Giallo R, Woolhouse H, Gartland D, Hiscock H, Brown S. The emotional–behavioural functioning of children exposed to maternal depressive symptoms across pregnancy and early childhood: a prospective Australian pregnancy cohort study. Eur Child Adolesc Psychiatry 2015;24:1233–44. https://doi.org/10.1007/s00787-014-0672-2.

For peer review only - http://bmjopen.bmj.com/site/about/guidelines.xhtml

[12] Hardcastle K, Bellis MA, Ford K, Hughes K, Garner J, Ramos Rodriguez G. Measuring the relationships between adverse childhood experiences and educational and employment success in England and Wales: findings from a retrospective study. Public Health 2018;165:106–16. https://doi.org/10.1016/J.PUHE.2018.09.014.

[13] Posmontier B, Neugebauer R, Stuart S, Chittams J, Shaughnessy R. Telephone-Administered Interpersonal Psychotherapy by Nurse-Midwives for Postpartum Depression. J Midwifery Womens Health 2016;61:456–66. https://doi.org/10.1111/jmwh.12411.

[14] Zheng Q, Shi L, Zhu L, Jiao N, Chong YS, Chan SWC, et al. Cost-effectiveness of Web-Based and Home-Based Postnatal Psychoeducational Interventions for First-time Mothers: Economic Evaluation Alongside Randomized Controlled Trial. J Med Internet Res 2022;24. https://doi.org/10.2196/25821.

[15] The National Institute of Health and Care Excellence. NICE | The National Institute for Health and Care Excellence 2022. https://www.nice.org.uk/ (accessed February 9, 2022).

[16] National Institute for Health and Care Excellence. Maternity and mental health 2022. https://www.nice.org.uk/about/what-we-do/into-practice/measuring-the-use-of-nice-guidance/impact-of-our-guidance/niceimpact-maternity/ch2-maternity-and-mental-health (accessed July 27, 2022).

[17] Camacho EM, Shields GE. Cost-effectiveness of interventions for perinatal anxiety and/or depression: A systematic review. BMJ Open 2018;8. https://doi.org/10.1136/bmjopen-2018-022022.

[18] Moran PS, Wuytack F, Turner M, Normand C, Brown S, Begley C, et al. Economic burden of maternal morbidity – A systematic review of cost-of-illness studies. PLoS One 2020;15. https://doi.org/10.1371/JOURNAL.PONE.0227377.

[19] Gurung B, Jackson LJ, Monahan M, Butterworth R, Roberts TE. Identifying and assessing the benefits of interventions for postnatal depression: A systematic review of economic evaluations. BMC Pregnancy Childbirth 2018;18. https://doi.org/10.1186/S12884-018-1738-9.

[20] Morrell CJ, Sutcliffe P, Booth A, Stevens J, Scope A, Stevenson M, et al. A systematic review, evidence synthesis and meta-analysis of quantitative and qualitative studies evaluating the clinical effectiveness, the cost-effectiveness, safety and acceptability of interventions to prevent postnatal depression. Health Technol Assess 2016;20:1–414. https://doi.org/10.3310/HTA20370.

[21] Spencer LH, Pisavadia K, Tuersley L, Edwards RT. A structured review of the costs and services for perinatal anxiety and associated disorders: MAP ALLIANCE. PROSPERO 2022 CRD42022347859. PROSPERO 2022.

For peer review only - http://bmjopen.bmj.com/site/about/guidelines.xhtml

[22] Page M, McKenzie J, Bossuyt P, Boutron I, Hoffmann T, Mulrow C. The PRISMA 2020 statement: an updated guideline for reporting systematic reviews. BMJ 2021;10. https://doi.org/doi.org/10.1186/s13643-021-01626-4.

[23] Health and Care Research Wales. Impact of the COVID-19 pandemic on the health and access to health care of disabled people: a rapid review 2022. https://healthandcareresearchwales.org/impact-covid-19-pandemic-disabled-people (accessed May 10, 2022).

[24] Health and Care Research Wales. Modelling studies used to evaluate the effect of population-level non-pharmaceutical interventions on the reproduction number of Severe Acute Respiratory Syndrome Coronavirus 2 (SARS-CoV-2) 2022. https://healthandcareresearchwales.org/modelling-studies-used-evaluate-effect-population-level-non-pharmaceutical-interventions (accessed May 10, 2022).

[25] Tricco AC, Khalil H, Holly C, Feyissa G, Godfrey C, Evans C, et al. Rapid reviews and the methodological rigor of evidence synthesis. JBI Evid Synth 2022;Publish Ah:944–9. https://doi.org/10.11124/jbies-21-00371.

[26] Mandrik O, Severens J, Bardach A, Ghabri S, Hamel C, Mathes T, et al. Critical Appraisal of Systematic Reviews With Costs and Cost-Effectiveness Outcomes: An ISPOR Good Practices Task Force Report 2021. https://doi.org/10.1016/j.

[27] Schardt C, Adams MB, Owens T, Keitz S, Fontelo P. Utilization of the PICO framework to improve searching PubMed for clinical questions. BMC Med Inform Decis Mak 2007;7:1–6.

[28] Drummond MF, Sculpher MJ, Claxton K, Stoddart GL, Torrance G W. Methods for the economic evaluation of health care programmes. 4th ed. Oxford: Oxford University Press; 2015.

[29] Moons KGM, de Groot JAH, Bouwmeester W, Vergouwe Y, Mallett S, Altman DG, et al. Critical Appraisal and Data Extraction for Systematic Reviews of Prediction Modelling Studies: The CHARMS Checklist. PLoS Med 2014;11. https://doi.org/10.1371/journal.pmed.1001744.

[30] Aromataris E, Fernandez R, Godfrey C, Holly C, Kahlil H, Tungpunkom P. Summarizing systematic reviews: methodological development, conduct and reporting of an Umbrella review approach. Int J Evid Based Healthc 2015 2015;13:132–40.

[31] Tufanaru C, Munn Z, Aromataris E, Campbell J, Hopp L. Systematic reviews of effectiveness. In: Aromataris E, Munn Z, editors. JBI Manual for Evidence Synthesis, JBI; 2020.

[32] Moola S, Munn Z, Tufanaru C, Aromataris E, Sears K, Sfetcu R, et al. Systematic reviews of etiology and risk. In: Aromataris E, Munn Z, editors. Manual for Evidence Synthesis, JBI; 2020.

For peer review only - http://bmjopen.bmj.com/site/about/guidelines.xhtml

[33] Morrell CJ, Spiby H, Stewart P, Walters S, Morgan A. Costs and effectiveness of community postnatal support workers: Randomised controlled trial. Br Med J 2000;321:593–8. https://doi.org/10.1136/BMJ.321.7261.593.

[34] Henderson C, Dixon S, Bauer A, Knapp M, Jane Morrell C, Slade P, et al. Cost-effectiveness of PoNDER health visitor training for mothers at lower risk of depression: Findings on prevention of postnatal depression from a cluster-randomised controlled trial. Psychol Med 2019;49:1324–34. https://doi.org/10.1017/S0033291718001940.

[35] Ride J, Lorgelly P, Tran T, Wynter K, Rowe H, Fisher J. Preventing postnatal maternal mental health problems using a psychoeducational intervention: the cost-effectiveness of What Were We Thinking. BMJ Open 2016;6. https://doi.org/10.1136/bmjopen-2016.

[36] Stevenson MD, Scope A, Sutcliffe PA. The Cost-Effectiveness of Group Cognitive Behavioral Therapy Compared with Routine Primary Care for Women with Postnatal Depression in the UK. Value in Health 2010;13:580–4. https://doi.org/10.1111/j.1524-4733.2010.00720.x.

[37] Grote NK, Simon GE, Russo J, Lohr MJ, Carson K, Katon W. Incremental benefit-cost of MOMCare: Collaborative care for perinatal depression among economically disadvantaged women. Psychiatric Services 2017;68:1164–71. https://doi.org/10.1176/APPI.PS.201600411.

[38] Petrou S, Cooper P, Murray L, Davidson LL. Economic costs of post-natal depression in a high-risk British cohort. British Journal of Psychiatry 2002;181:505–12. https://doi.org/10.1192/bjp.181.6.505.

[39] Dagher RK, McGovern PM, Dowd BE, Gjerdingen DK. Postpartum depression and health services expenditures among employed women. J Occup Environ Med 2012;54:210–5. https://doi.org/10.1097/JOM.0B013E31823FDF85.

[40] Ammerman RT, Chen J, Mallow PJ, Rizzo JA, Folger AT, Van Ginkel JB. Annual direct health care expenditures and employee absenteeism costs in high-risk, low-income mothers with major depression. J Affect Disord 2016;190:386–94. https://doi.org/10.1016/J.JAD.2015.10.025.

[41] Roberts J, Sword W, Watt S, Gafni A, Krueger P, Sheehan D, et al. Costs of postpartum care examining associations from the Ontario mother and infant survey. Canadian Journal of Nursing Research 2001;33:19–34.

[42] Counts NZ, Kuklinski MR, Wong V, Feinberg ME, Creedon TB. Comparison of Estimated Incentives for Preventing Postpartum Depression in Value-Based Payment Models Using the Net Present Value of Care vs Total Cost of Care. JAMA Netw Open 2022;5:e229401. https://doi.org/10.1001/JAMANETWORKOPEN.2022.9401.

[43] Franta G, Hersh AR, Cirino NH, Caughey AB. Prevention of perinatal depression with counseling in adolescents: a cost-effectiveness analysis. The Journal of Maternal-Fetal & Neonatal Medicine: The Official Journal of the

For peer review only - http://bmjopen.bmj.com/site/about/guidelines.xhtml

European Association of Perinatal Medicine 2022. https://doi.org/10.1080/14767058.2022.2049746.

[44] Ride J. Setting the Boundaries for Economic Evaluation: Investigating Time Horizon and Family Effects in the Case of Postnatal Depression. Value in Health 2018;21:573–80. https://doi.org/10.1016/j.jval.2017.10.016.

[45] Chojenta C, William J, Martin MA, Byles J, Loxton D. The impact of a history of poor mental health on health care costs in the perinatal period. Arch Womens Ment Health 2019;22:467–73. https://doi.org/10.1007/S00737-018-0912-4.

[46] Bank of England. Inflation calculator 2022. https://www.bankofengland.co.uk/monetary-policy/inflation/inflation-calculator (accessed August 18, 2022).

[47] U.S. Bureau of Labor Statistics. CPI Inflation Calculator 2022. https://www.bls.gov/data/inflation_calculator.htm (accessed August 18, 2022).

[48] Google. Google currency converter 2022. https://www.google.com/search?q=currency+convertor&rlz=1C1GCEA_enGB991GB991&oq=currency+convertor&aqs=chrome..69i57j0i10i131i433l2j0i10l7.4286j1j3&sourceid=chrome&ie=UTF-8 (accessed September 5, 2022).

[49] Bank of Canada. Inflation Calculator 2022. https://www.bankofcanada.ca/rates/related/inflation-calculator/ (accessed August 18, 2022).

[50] Reserve Bank of Australia 2022. https://www.rba.gov.au/calculator/annualDecimal.html (accessed March 29, 2023).

[51] Henderson J. The cost effectiveness of prenatal care - ProQuest 1994. https://www.proquest.com/assia/docview/196971225/abstract/36297FA264EA438FPQ/1?accountid=14874 (accessed May 21, 2022).

[52] Cox JL, Chapman G, Murray D, Jones P. Validation of the Edinburgh postnatal depression scale (EPDS) in non-postnatal women. vol. 39. 1996.

[53] Ride J. Setting the Boundaries for Economic Evaluation: Investigating Time Horizon and Family Effects in the Case of Postnatal Depression 2018. https://doi.org/10.1016/j.jval.2017.10.016.

[54] Davis J, Ohan JL, Gibson LY, Prescott SL, Finlay-Jones AL. Understanding engagement in digital mental health and wellbeing programs for sub-clinical women in the perinatal period: A Systematic Review without Meta-Analysis (SWiM) (Preprint). J Med Internet Res 2022. https://doi.org/10.2196/36620.

[55] Ayers S, Coates R, Sinesi A, Best C, Shakespeare J, Hollins K, et al. OptiMising cAre for Perinatal Anxiety: evaLuation of heaLth servIce utilisAtioN, outComEs and costs (MAP ALLIANCE) - NIHR Funding and Awards. National Institute for Health and Care Research 2022.

For peer review only - http://bmjopen.bmj.com/site/about/guidelines.xhtml

https://fundingawards.nihr.ac.uk/award/NIHR133727 (accessed August 16, 2022).

For peer review only

For peer review only

Figure 1. PRISMA study selection flowchart (Page et al., 2021b)

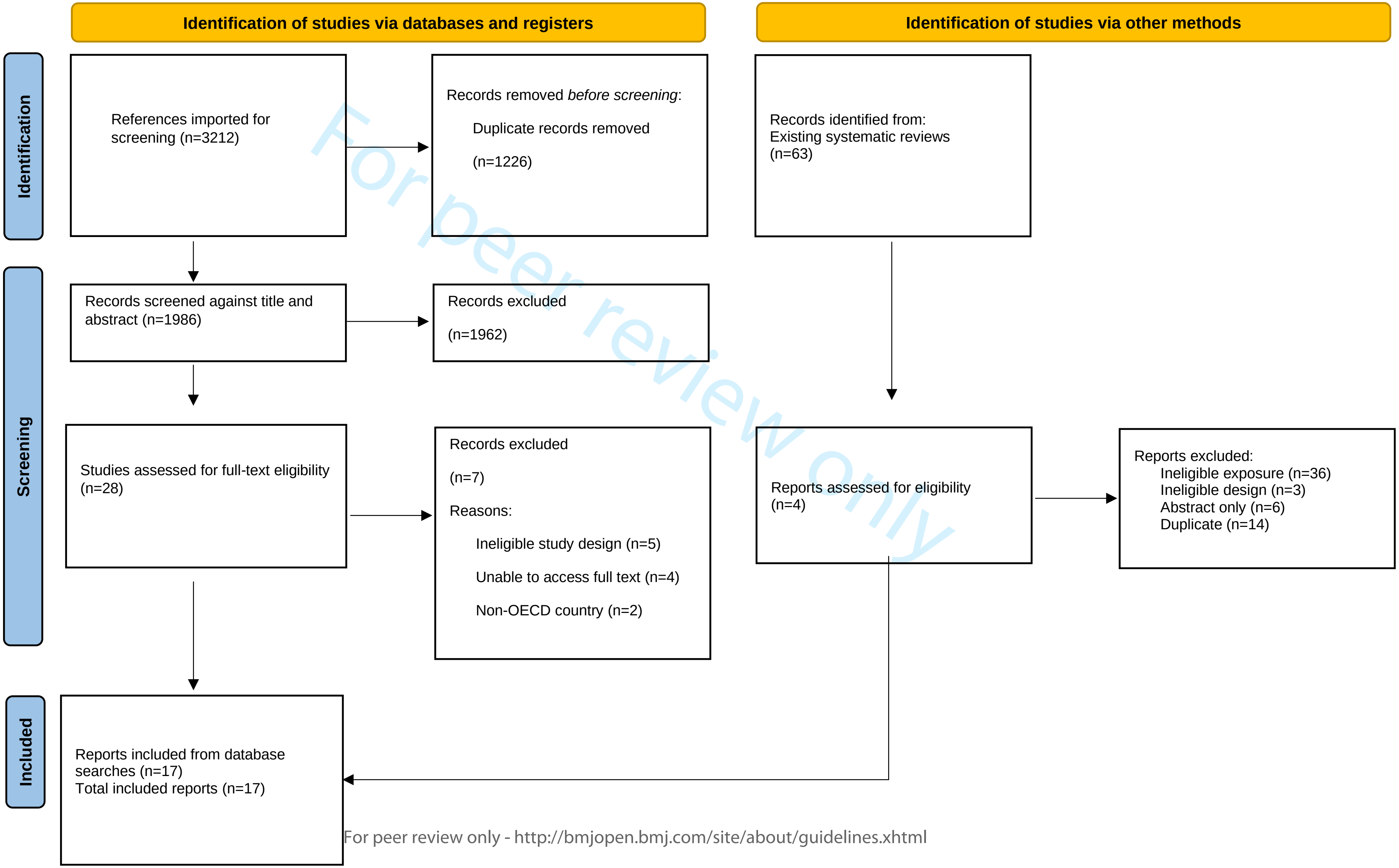

For peer review only

For peer review only - http://bmjopen.bmj.com/site/about/guidelines.xhtml

Search strategy

Below is an example of a search strategy for the Medline database.

ID	Search terms
1	exp Pregnancy/
2	(pregnan* or childbearing).ti,ab,kw.
3	(postpartum or post-partum or postnatal or postnatal or perinatal or peri-natal or prenatal or pre-natal or antenatal or ante-natal or matern*).ti,ab,kw.
4	perinatal anxiety.ti,ab,kw. or exp Perinatal anxiety/
5	exp Depression/
6	depress$.ti,ab,kw.
7	5 or 6
8	(Infant or baby or child).ti,ab,kw
9	(care* or treatment).tiab.kw
10	NHS.ti,ab,kw
11	hospitali$ation*.ti,ab,kw
12	exp Resource allocation/
13	economic evaluation$.ti,ab,kw.
14	(cost* or economic* or pharmacoeconomic*).ti.
15	13 or 14
16	exp "costs and cost analysis"/ or exp Health Care Costs/
17	exp Cost-Benefit Analysis/
18	(cost* adj2 (effective* or utility* or benefit* or consequence* or minimi*)).ti,ab,kw.
19	16 or 17 or 18
20	quality-adjusted life year$.ti,ab,kw. or exp Quality-Adjusted Life Years/
21	Or 7 and 15 and 19

For peer review only

Quality appraisal of health economic evaluation studies [24]

Drummond et al checklist 2015	Petrou et al (2002) [40]	Petrou et al (2006) [4]	Ride et al (2016) [32]	Henderson et al (2019) [38]
1. Was a well defined question posed in an answerable form?	Yes	Yes	Yes	Yes
2. Was a comprehensive description of the competing alternatives given?	n/a	n/a	Yes	Yes
3. Was the effectiveness of the programs or services established?	n/a	n/a	Yes	Yes
4. Were all the important and relevant costs and consequences for each alternative identified?	n/a	n/a	Yes	Yes
5. Were costs and consequences measured accurately in appropriate physical units?	Yes	Yes	Yes	Yes
6. Were costs and consequences valued credibly?	Yes	Yes	Yes	Yes
7. Were costs and consequences adjusted for differential timing	n/a	n/a	No	No
8. Was an incremental analysis of costs and consequences of alternatives performed?	n/a	n/a	No	Yes
9. Was allowance made for uncertainty in the estimates of costs and consequences?	Yes	Yes	Yes	Yes
10. Did the presentation and discussion of study results include all issues of concern to users?	Yes	Yes	Yes	Yes

Source of checklist: Drummond, M. F., Sculpher, M. J., Claxton, K., Stoddart, G. L., & Torrance G W. (2015). *Methods for the economic evaluation of health care programmes* (4th ed.). Oxford: Oxford University Press.

For peer review only - http://bmjopen.bmj.com/site/about/guidelines.xhtml

Quality appraisal of health economic modelling studies with CHARMS Checklist [25]

Domain	Key items	**Counts et al (2022) -** [34]	**Franta et al (2022) -** (Franta et al., 2022)	**Ride (2018)** - [45]	**Wilkinson et al (2017) -** [5]	**Bauer et al (2015)** (Bauer et al, 2015)	**Stevenson et al, (2010)** [42]
**SOURCE OF DATA**	Source of data (e.g., cohort, case-control, randomized trial participants.	p.3	p.2	p.575	p.3	p.52	p.581
**PARTICIPANTS**	Participant eligibility and recruitment method (e.g., consecutive participants, location,	p.3	p.2	p.575	p.3	p.52	p.581
	Participant description	p.3	p.2	p.575	p.3	p.52	p.581
	Details of treatments received, if	p.5	p.2	p.575	p.3	p.52	N/A
	Study dates	p.4	p.2	p.575	p.3	p.52	p.581
**OUTCOME(S) TO BE PREDICTED**	Definition and method for measurement of outcome	p.4	p.2	p.574	p.4	p.53	p.581-582
	Was the same outcome definition (and method for measurement) used in all	Yes p.5	p.2	p.574	p.4	p.53	p.581-582
	Type of outcome (e.g., single or combined	p.3	p.5	p.574	p.4	p.53	p.581
	Was the outcome assessed without knowledge of	No	No	No	No	No	p.581
	Were candidate predictors part of the outcome	No	No	No	No	No	p.581

For peer review only - http://bmjopen.bmj.com/site/about/guidelines.xhtml

	Time of outcome occurrence or summary of duration of follow-up	p.5	p.5	p.578	p.4	p.52	p.581
**CANDIDATE PREDICTORS (OR INDEX TESTS)**	Number and type of predictors (e.g., demographics, patient history, physical examination,	p.5	p.5	p.577	p.6	p.55	p.582
	Definition and method for measurement of candidate predictors	p.5	p.5	p.575	p.6	p.55	p.580-582
	Timing of predictor measurement (e.g., at patient presentation, at diagnosis,	p.5	p.5	p.577	p.6	p.55	p.581
	Were predictors assessed blinded for outcome, and for each other	No	No	No	No	No	p.582
	Handling of predictors in the modelling (e.g., continuous, linear, non-linear transformatio	Unclear	Unclear	Unclea r	Unclear	p.52	p.582
**SAMPLE SIZE**	Number of participants and number of outcomes/ev	p.3	p.2	p.575	P.3	p.55	p.582
	Number of outcomes/ev ents in relation to the number of candidate predictors (Events Per Variable)	p.5	p.3	p.577	p.20	p.57	p.582
**MISSING DATA**	Number of participants with any missing value (includ	p.4	Unclear	Unclea r	Unclear	Unclea r	Unclear

For peer review only - http://bmjopen.bmj.com/site/about/guidelines.xhtml

	Number of participants with missing data for each predictor	Unclear	Unclear	Unclea r	Unclear	Unclea r	Unclear
	Handling of missing data (e.g., complete-case analysis, imputation, or	Unclear	Unclear	Unclea r	Unclear	Unclea r	Unclear
**MODEL DEVELOPMEN T**	Modelling method (e.g., logistic, survival, neural network, or	Simulate d cohort model	Simulat ed cohort model	Decisi on analyti c model	Simulat ed cohort model	Decisi on analyti c model	Mathemati cal model
	Modelling assumptions satisfied	See Appendix 1 in the supplem ent	p.5	p.577	p.4	p.53	p.580
	Method for selection of predictors **for inclusion** in multivariable modelling (e.g., all candidate predictors, pre-selection based on unadjusted association with the	Unclear	Unclear	p.577	p.4	p.53	p.581
	Method for selection of predictors **during multivariable modelling** (e.g., full model approach, backward or forward selection) and criteria used (e.g., p-value, Akaike Information Criterion)	Unclear	Unclear	Unclea r	Unclear	p.53	Unclear

For peer review only - http://bmjopen.bmj.com/site/about/guidelines.xhtml

	Shrinkage of predictor weights or regression coefficients (e.g., no shrinkage, [illegible]	Unclear	Unclear	Unclear	Unclear	Unclear	Unclear
**MODEL PERFORMANCE**	Calibration (calibration plot, calibration slope, Hosmer-Lemeshow test) and Discrimination (C-statistic, D-statistic, log-rank)	p.5	Unclear	Unclear	Unclear	Unclear	Unclear
	Classification measures (e.g., sensitivity, specificity, predictive values, net reclassification improvement) and whether a-priori cut [illegible]	See e-appendix 3	p.6	p.577	p.6	No	p.581
**MODEL EVALUATION**	Method used for testing model performance: development dataset only (random split of data, resampling methods e.g. bootstrap or cross-validation, none) or separate external validation (e.g. temporal, geographical	See e-appendix 3	Unclear	Unclear	p.6	No	Unclear

For peer review only

	In case of poor validation, whether model was adjusted or updated (e.g., intercept recalibrated, predictor effects	Unclear	Unclear	Unclea r	Unclear	No	Unclear
**RESULTS**	Final and other multivariable models (e.g., basic, extended, simplified) presented, including predictor weights or regression coefficients, intercept, baseline survival, model performance measures (with	Unclear	Unclear	Unclea r	Unclear	No	No
	Any alternative presentation of the final prediction models, e.g., sum score, nomogram, score chart, predictions for specific risk subgroups	No	No	p.578	p.23	No	No
	Comparison of the distribution of predictors (including missing data) for development and validation	No	No	No	No	No	No

For peer review only

**INTERPRETATION AND DISCUSSION**	Interpretation of presented models (confirmatory, i.e., model useful for practice versus exploratory, i.e., more research	p.7	p.5	p.577	p.6	p.56	p.583
	Comparison with other studies, discussion of generalizability, strengths and limitations.	p.7	p.5	p.577	p.6	p.58	p.583

JBI critical appraisal checklist for Systematic Reviews and Research Syntheses [26]

Citation	Q1. Is the review question clearly and explicitly stated?	Q2. Were the inclusion criteria appropriate for the review question?	Q3. Was the search strategy appropriate?	Q4. Were the sources and resources used to search for studies adequate?	Q5. Were the criteria for appraising studies appropriate?	Q6. Was critical appraisal conducted by two or more reviewers independently?	Q7. Were there methods to minimize errors in data extraction?	Q8. Were the methods used to combine studies appropriate?	Q9. Was the likelihood of publication bias assessed?	Q10. Were recommendations for policy and/or practice supported by the reported data?	Q11. Were the specific directives for new research appropriate?
(Camacho & Shields, 2018)	Yes	Yes	Yes	Yes	Yes	Unclear	Yes	Yes	Yes	Yes	Yes
[37]	Yes	Yes	Yes	Yes	Yes	Unclear	Yes	Unclear	Yes	Yes	Yes
(Moran et al., 2020)	Yes	Yes	Yes	Yes	Yes	Yes	Yes	Yes	Yes	Unclear	Yes
(Morrell et al., 2016)	Yes	Yes	Yes	Yes	Yes	Yes	Yes	Yes	Yes	Yes	Yes

JBI Critical appraisal checklist for randomized controlled trials [27]

Citation	Q1. Was true randomization used for assignment of participants to treatment groups?	Q2. Was allocation to treatment groups concealed?	Q3. Were treatment groups similar at the baseline?	Q4. Were participants blind to treatment assignment?	Q5. Were those delivering treatment blind to treatment	Q6. Were outcomes assessors blind to treatment	Q7. Were treatment groups treated identically other than the	Q8. Was follow up complete and if not, were differences	Q9. Were participants analyzed in the groups to which they were	Q10. Were outcomes measured in the same way for	Q11. Were outcomes measured in a reliable way?	Q12. Was appropriate statistical analysis used?	Q13. Was the trial design appropriate, and any deviations from the standard

8

					assignment?	assignment?	intervention of interest?	between groups in terms of their follow up adequately described and analyzed?	randomized?	treatment groups?			RCT design (individual randomization, parallel groups) accounted for in the conduct and analysis of the trial?
[29]	Yes	Yes	Yes	Unclear	Yes	Unclear	Yes	Yes	No	Yes	Unclear	Yes	N/A
[44]	Yes	Yes	Yes	Yes	Yes	Unclear	Yes	Yes	Yes	Yes	Yes	Yes	Yes

JBI Critical Appraisal Checklist for Cohort Studies [28]

Citation	Q1. Were the two groups similar and recruited from the same population?	Q2. Were the exposures measured similarly to assign people to both exposed and unexposed groups?	Q3. Was the exposure measured in a valid and reliable way?	Q4. Were confounding factors identified?	Q5. Were strategies to deal with confounding factors stated?	Q6. Were the groups/ participants free of the outcome at the start of the study (or at the moment of exposure)?	Q7. Were the outcomes measured in a valid and reliable way?	Q8. Was the follow up time reported and sufficient to be long enough for outcomes to occur?	Q9. Was follow up complete, and if not, were the reasons to loss to follow up described and explored?	Q10. Were strategies to address incomplete follow up utilized?	Q11. Was appropriate statistical analysis used?
(Moore Simas et al., 2020)	Yes	Yes	Yes	No	No	Yes	Yes	Yes	Unclear	N/A	Yes

JBI Critical Appraisal Checklist for Cross-sectional studies [28]

Citation	Q1. Were the criteria for inclusion in the sample clearly defined?	Q2. Were the study subjects and the setting described in detail?	Q3. Was the exposure measured in a valid and reliable way?	Q4. Were objective, standard criteria used for measurement of the condition?	Q5. Were confounding factors identified?	Q6. Were strategies to deal with confounding factors stated?	Q7. Were the outcomes measured in a valid and reliable way?	Q8. Was appropriate statistical analysis used?
Dagher et al., 2012	Yes	Yes	Yes	Yes	No	No	Yes	Yes
Chojenta et al., 2019	Yes	Yes	Yes	Yes	Yes	Yes	Yes	Unclear
Ammerman et al., 2016	Yes	Yes	Yes	Yes	Yes	Yes	Yes	Yes
Roberts et al., 2001	Yes	Yes	Yes	Yes	No	N/A	Yes	Yes

For peer review only - http://bmjopen.bmj.com/site/about/guidelines.xhtml

Abbreviations

Abbreviation	Full	Aspect
ANOVA	Analysis of Variance	Analysis
ANRQ-R	Antenatal Risk Questionnaire	Tool
CATi	Computer Assisted Telephone Interviews	Research
CBA	Cognitive Behavioural Approach	Intervention
CBT	Cognitive Behavioural Therapy	Intervention
CEA	Cost Effectiveness Analysis	Analysis
CIDI	Composite International Diagnostic Interview	Research
CUA	Cost Utility Analysis	Analysis
DASS21	Depression, Anxiety and Stress Scale	Tool
DCS	Depression Care Specialist	Staff
DFD	Disease Free Day	Research
DSM-IV	Diagnostic and Statistical Manual for Mental Disorders 4th Edition	Source
eMBI	electronic Mindfulness-based Intervention	Intervention
EPDS	Edinburgh Postnatal Depression Scale	Tool
ePRO	electronic Patient Reported Outcomes	Research
EQ-5D-3L	EuroQol 5 Dimension 3 Level	Tool
GP	General Practitioner	Staff
gCBT	Group cognitive behavioural therapy	Intervention
HRU	Healthcare resource utilization	Analysis
HV	Health Visitor	Staff
ICD	International Classification of Diseases	Source
ICER	Incremental Cost-Effectiveness Ratio	Analysis
IG	Intervention Group	Research
IPT	Interpersonal psychotherapy	Intervention
ITT	Intention to Treat	Research
LGA	Local Government Area	Organisation
MBS	Medical Benefits Schedule	Source
MCH	Maternal and Child Health	Setting
MFAS	Maternal-Fetal Attachment Scale	Tool
MOMcare		*Study name*
MINI	Mini-International Neuropsychiatric Interview	Tool
NHS	National Health Service	Setting
OOP	Out of Pocket	Research
PAD	perinatal anxiety and/or depression	Diagnosis
PBS	Pharmaceutical Benefits Scheme	Source
PCA	Personalised Care Approach	Intervention
PHQ-9	Patient Health Questionnaire	Tool
PND	Postnatal depression	Diagnosis
PND	Post-partum depression	Diagnosis
PoNDER trial	POstNatal Depression Economic evaluation and Randomised trial	*Study name*
PRAQ-R	Pregnancy-Related Anxiety Questionnaire	Tool
PTSD	Post-Traumatic Stress Disorder	Diagnosis
QALY	Quality Adjusted Life Year	Analysis

For peer review only - http://bmjopen.bmj.com/site/about/guidelines.xhtml

RCT	Randomised controlled trial	Research
SCL-20	Hopkins Symptom Checklist-20	Tool
SF36	Short-Form 36	Tool
SIDS	Sudden infant death syndrome	Diagnosis
SPARCS	*Sleep, Parenting and Relationships in a Community Setting*	*Study name*
STAI	State-Trait Anxiety Questionnaire	Tool
TAU	Treatment as Usual	Research
TENS	Transcutaneous Electrical Nerve Stimulation	Intervention
WHO	World Health Organisation	Organisation
WWWT	What Were We Thinking	Tool

For peer review only

Data extraction table for studies including perinatal anxiety

Citation (Country)	Study Details	Participants and setting	Key findings	Observations
Ride et al (2016)<br><br>(Ride et al., 2016)<br><br>**Australia**	**Study Design:** Economic evaluation, including cost-effectiveness and cost-utility analyses, conducted alongside a cluster-randomised trial<br><br>**Type of intervention [*exposure*]:** What Were We Thinking (WWWT) - a psychoeducational intervention targeted at the partner relationship, management of infant behaviour and parental fatigue.<br><br>**Data collection methods:** Data were collected from participants via computer-assisted telephone interview at baseline (6 weeks postpartum) and follow-up (26 weeks postpartum).	**Sample size:** 359<br><br>**Participants:** English-speaking first-time mothers who had recently given birth and attended participating Maternal and Child Health Centres (MCHCs)<br><br>**Setting:** 48 Maternal and Child Health Centres in Victoria, Australia.<br><br>**Dates of data collection:** Baseline interviews took place between May 2013 and April 2014, and follow-up interviews between September 2013 and August 2014.	**Primary Findings:**<br>The intervention was estimated to cost $A118.16 per participant. The analysis showed no statistically significant difference between the intervention and control groups in costs or outcomes. The incremental cost-effectiveness ratios were $A36 451 per QALY gained and $A152 per percentage point reduction in 30-day prevalence of depression, anxiety, and adjustment disorders. The estimate lies under the unofficial cost-effectiveness threshold of $A55 000 per QALY; however, there was considerable uncertainty surrounding the results, with a 55% probability that WWWT would be considered cost-effective at that threshold.<br>**Additional Findings:**<br>The results suggest that, although WWWT shows promise as a preventive intervention for postnatal maternal mental health problems, further research is required to reduce the uncertainty over its cost-effectiveness as there were no statistically significant differences in costs or outcomes.	Ride et al (2016) investigated the cost-effectiveness of the What Were We Thinking (WWWT) intervention, for the prevention of postnatal maternal mental health problems. The intervention was estimated to cost $A118.16 per participant. The analysis showed no statistically significant difference between the intervention and control groups in costs or outcomes. The incremental cost-effectiveness ratios were $A36 451 per QALY gained and $A152 per percentage point reduction in 30-day prevalence of depression, anxiety, and adjustment disorders. The estimate lies under the unofficial cost-effectiveness threshold of $A55 000 per QALY; however, there was considerable uncertainty surrounding the results, with a 55% probability that WWWT would be considered cost-effective at that threshold

For peer review only - http://bmjopen.bmj.com/site/about/guidelines.xhtml

Data extraction table for studies including maternal depression

Citation (Country)	Study Details	Participants and setting	Key findings	Observations
Ammerman et al (2016)<br><br>(Ammerman et al., 2016)<br><br>USA	**Study design:** Cross-sectional<br><br>**Data collection methods:** MEPS database, a subset of the National Health Interview Survey (NHIS) that includes information on health care utilisation and expenditures for the civilian, non-institutionalised population in the USA.	**Sample size:** 20,531<br><br>**Participants:** 2,310 high-risk mothers with depression and 18,221 high-risk mothers without depression<br><br>**Setting:** USA healthcare setting<br><br>**Dates of data collection:** 1996 to 2011	**Primary findings:**<br>Depressed mothers were more likely to incur insurer (0.88 vs. 0.80) and out of pocket expenses (0.86 vs. 0.77) and to have higher insurer expenses ($4916 vs. $3521) and out of pocket expenses ($786 vs. $522) (in 2015).<br>**Additional findings:**<br>A higher proportion of the depressed sample was Caucasian which were in relatively worse health than women from other ethnic groups. The depressed sample was more likely to have public insurance, to be English-speaking and to have a usual health care provider.	The cross-sectional study from Ammerman et al (2016) the USA conducted between 2006 and 2011 investigated the out-of-pocket expenses and insurer expenses of depressed vs non-depressed mothers. Depressed mothers were more likely to incur insurer and out of pocket expenses and to have higher insurer expenses ($4916 vs. $3521) and out of pocket expenses ($786 vs. $522) (in 2015).
Bauer et al (2015)<br><br>(Bauer et al., 2015)<br><br>UK	**Study Design:** The economic analysis takes a life-course perspective from the viewpoints of the public sector, individual and society. The study analysed the effects of perinatal depression on child development outcomes of children at ages 11 and 16 years from the community-based South London Child Development Study. Economic consequences were attached to those outcomes through simple decision-analytic techniques, building on evidence from studies of epidemiology, health-related quality of life, public sector costs and employment.	**Sample size:** 120<br><br>**Participants:** Mothers and children<br><br>**Setting:** Two antenatal clinics in the UK<br><br>**Dates of data collection:** January to December 1986	**Primary Findings:**<br>Additional risks that children exposed to perinatal depression develop emotional, behavioural, or cognitive problems ranged from 5% to 21%. In addition, there was a high risk (24%) that children would have special educational needs.<br><br>For each child exposed to perinatal depression, public sector costs exceeded £3,030, costs due to reduced earnings were £1,400 and health-related quality of life loss was valued at £3,760.	The study examined some of the outcomes and long-term economic implications experienced by offspring who have been exposed to perinatal depression.
Counts et al (2022)<br><br>(Counts et al., 2022)<br><br>USA	**Study Design:** Modelling study. A decision analytic model used a simulated cohort of 1,000 Medicaid-enrolled pregnant individuals. Health care costs for individuals receiving postpartum depression preventive intervention or not, over 1 or 5 years postpartum, in a variety of scenarios, including varying rates of Medicaid churn (i.e., transitions to a new Medicaid managed care plan, commercial insurance plan, or loss of coverage) were	**Sample size:** 1,000<br><br>**Participants:** simulated cohort of 1,000 Medicaid enrolled pregnant individuals<br><br>**Setting:** USA healthcare system.	**Primary Findings:**<br>The main outcome was the amount of clinician incentive shared in a Value-based payment (VBP) model from providing preventive interventions. The likelihood of the health care payer realising a positive return on investment if it shared 50% of 5-year	This economic modelling study found that providing preventive interventions for PND resulted in an estimated 5-year saving of £602▫

2

For peer review only - http://bmjopen.bmj.com/site/about/guidelines.xhtml

	estimated for the period 2020 to 2025. The model was developed between March 5 2021 and July 30 2021.<br><br>**Type of intervention [*exposure*]:** Individual counselling and group-based counselling.<br><br>**Data collection methods:** Simulation based on collected Medicaid data.	**Dates of data collection:** Model developed between March 5 2021 and July 30 2021.	expected savings with a clinician up front was also measured.<br><br>The simulated cohort was designed to be reflective of the demographics characteristics of pregnant individuals receiving Medicaid; however, no specific demographic features were simulated. Providing preventive interventions for postpartum depression resulted in an estimated 5-year savings of $734.12 (95% credible interval [CrI], $217.21-$1235.67) per person. Without health insurance churn, sharing 50% of 5-year expected savings could offer more than double the financial incentives for clinicians to prevent postpartum depression compared with traditional VBP ($367.06 [95% CrI, $108.61-$617.83] vs $177.74 [95% CrI, $52.66-$296.60], respectively), with a high likelihood of positive return for the health care payer (91%). As health insurance churn increased, clinician incentives from sharing estimated savings decreased (73% reduction with 50% annual churn).	
Dagher et al (2012)<br><br>(Dagher et al., 2012<br><br>USA	**Study design:** Cross-sectional<br><br>**Data collection methods:** Prices of service use and EPDS	**Sample size:** 638 women.<br><br>**Participants:** Women receiving maternal healthcare services, from hospital discharge to 11 weeks postpartum.<br><br>**Setting:** USA healthcare setting.<br><br>**Dates of data collection:** The year 2001.	**Primary findings:**<br>The total cost of all mental health counselling visits for the depressed group n =31 was $138 and the cost for the non-depressed group n= 607 was $13. This was a statistically significant difference ($p < 0.001$).<br><br>**Additional findings:** The total cost of emergency department visits for the postpartum women was $84 for the depressed group n = 31 and $13 for the non-depressed group n = 607. This was a statistically significant difference ($p < 0.001$).	The Dagher et al., (2012) cross-sectional study from the USA investigated expenditure from health care service from discharge until 11 weeks postpartum. There was a significant difference in healthcare expenditure between depressed and non-depressed women. The EPDS was used to measure depression. The total cost of all mental health counselling visits for the depressed group n =31 was $138 and the cost for the non-depressed group n= 607 was $13. This was a statistically significant difference ($p < 0.001$).

For peer review only

Franta et al (2022)<br><br>(Franta et al., 2022)<br><br>USA	**Study Design:**<br>Modelling study<br><br>**Type of intervention [*exposure*]:** Comparison of outcomes in pregnant adolescents who received versus did not receive counselling interventions<br><br>**Data collection methods**: Decision-analytic model using TreeAge Pro software	**Sample size:** Theoretical cohort of 180,000 individuals<br><br>**Participants:** pregnant adolescents<br><br>**Setting:** Obstetric setting<br><br>**Dates of data collection:** 2018	**Primary Findings:**<br>• A strategy of referral to counselling interventions was cost effective in the theoretical cohort, with 8,935 fewer cases of perinatal depression, 1,606 fewer cases of chronic depression, 166 fewer preterm deliveries, 4 fewer neonatal deaths, 1 fewer case of cerebral palsy, 20 fewer cases of SIDS. In total, there were 21,976 additional QALYs and cost savings of $223,549,872, making it the dominant strategy (better outcomes with lower costs).<br>• Counselling interventions remained cost saving until the annual direct and indirect cost of chronic, severe depression was set below $30,000, at which point it became cost effective (baseline input: $182,309).<br>• It is cost effective to refer all pregnant adolescents for preventive counselling interventions.	Using a theoretical cohort, Franta et al. (2022) found that counselling was a cost-effective preventative measure, leading to fewer cases of perinatal and chronic depression
Grote et al (2017)<br><br>Grote et al., 2017)<br><br>**USA**	**Study Design:** RCT, cost-benefit study<br><br>**Type of intervention [*exposure*]:** 18 months **MOMCare** collaborative care depression intervention (choice of brief interpersonal psychotherapy or pharmacotherapy or both) with enhanced maternity support services (MSS-Plus).<br><br>**Data collection methods:** Blinded telephone assessments, including depression severity on SCL-20. Unit costs of MOMCare intervention actual salary rate + fringe benefits + 30% overheads	**Sample size:** 152<br><br>**Participants:** 152 pregnant women 12-32 wks. gestation with probable major depression or dysthymia (PTSD). Plus 12 excluded from analysis due to missing final data.<br><br>**Setting:** 10 county public health centres<br><br>**Dates of data collection:** Recruited Jan 2010 – July 2012. Study ended 2014	**Primary Findings:** when controlled for baseline depression severity, women with probable depression and PTSD in MOMCare had 68 more depression-free days over 18 months than those in MSS-Plus (p,.05). Additional $1,312. depression care cost per MOMCare participant with comorbid PTSD. Incremental net benefit of MOMCare was positive if a depression free days was valued at $\geq$ $20<br><br>**Additional Findings:**<br>Unit costs used 2013:<br>$80 per 45-50 min depression care specialist (DCS) visit<br>$31 per 20-30 min DCS phone call<br>(Both included time for outreach efforts and record keeping)<br>$247 fixed cost per patient for caseload supervision and info support<br>Other references to US-based data sources	In this RCT, cost-benefit study, a multicomponent collaborative care intervention for depression (MOMcare - a choice of brief interpersonal psychotherapy or pharmacotherapy or both) with enhanced maternity support services (MSS-Plus) in the public health system of Seattle, USA. The incremental benefit and cost and the net benefit for women with major depression and PTSD was estimated. When controlled for baseline depression severity, women with probable depression and PTSD in MOMCare had 68 more depression-free days over 18 months than those in MSS-Plus (p<.05). There was an additional £1,943▪▪ depression care cost per MOMCare participant with comorbid PTSD. The incremental net benefit of MOMCare was positive if

For peer review only

For peer review only - http://bmjopen.bmj.com/site/about/guidelines.xhtml

				depression free days was valued below £18▪▪. For women with probable major depression and PTSD, MOMCare had a significant clinical benefit over MSS-Plus, with only a moderate increase in health services cost.[1]
Henderson et al (2019)<br><br>(Henderson et al., 2019)<br><br>**UK**	**Study Design:** PONDER Cluster RCT<br><br>**Type of intervention [*exposure*]:** GP practices assigned to usual health visitor (HV) care, HV trained to assess for PND plus offering either a CBA or a person-centred approach (PCA) weekly for 8 weeks<br><br>**Data collection methods:** Postal questionnaires: Baseline including EPDS and SF36 at 6 weeks, Postnatal questionnaires at 6, 12 and 18 months postnatal. Resource use logs were completed by HVs based on their and GP records	**Sample size: From** 101 GP practices, 4,084 participants consented, baseline data from 3,449 participants.<br><br>**Participants:** 2,241 lower risk women completed EPDS at 6 months – 767 control, 1,474 intervention. 1,459 women provided economic data.<br><br>**Setting:** GP practices<br><br>**Dates of data collection:** April 2003 for 3 years	**Primary Findings:** 99% probability of cost effectiveness at £20,000 at 6 months postnatal<br>Compared with controls, adjusted 6 months costs were £82 lower with the interventions<br><br>**Additional Findings:**<br>Little difference CBA to PCA – CBA marginally higher probability of being cost effective.	This study found that CBT had a marginally higher probability of being cost-effective than a person-centred approach.
Moore Simas et al (2020)<br><br>(Moore Simas et al., 2020)<br><br>USA	**Study Design:** Cohort study<br><br>**Type of intervention [*exposure*]:** PND.<br><br>**Data collection methods**: Administrative claims data from the IBM Watson Health MarketScan Databases	**Sample size:** 135,678<br><br>**Participants:** mother-child pairs with and without postpartum depression (PND) exposure<br><br>**Setting:** USA healthcare setting.<br><br>**Dates of data collection:** 2010 to 2016	**Primary Findings:**<br>• 33,314 mother-child pairs with PND exposure were propensity score matched to 102,364 mother-child pairs without PND exposure.<br>• During the 24-month follow-up period, HRU across most service categories was significantly higher among children in the PND exposure cohort than non-PND exposure cohort.<br>• Among outpatient services, the percentages of children with a physician specialist service (68% versus 64%), early-intervention screening (40% versus	This cohort study assessed healthcare resource utilisation (HRU) and costs in children of mothers with and without PND

1 ▪▪ Prices have been inflated and converted to GBP [53].

For peer review only - http://bmjopen.bmj.com/site/about/guidelines.xhtml

			37%), and an emergency room visit (48% versus 42%) were greater in children of mothers with PND (all *p* < .001).<br>• Furthermore, children of mothers with PND incurred 12% higher total healthcare costs in the first 24 months of life compared to children of mothers without PND ($24,572 versus $21,946; *p* < .001).<br>• After excluding mothers with preterm delivery, the proportion of children with ER visits, physician specialist services, and outpatient pharmacy claims was significantly higher in the PND exposure cohort than non-PND exposure cohort (all *p* < .001).<br>**Additional Findings:**<br>The results of this analysis suggest that HRU and costs over the first 24 months of life in children of mothers with PND exceeded that of children of mothers without evidence of PND.	
Petrou et al (2002)<br><br>(Petrou et al., 2002)<br><br>**UK**	**Study Design**: Economic evaluation in which unit costs were applied to resource-use data collected alongside a longitudinal study of women at high risk of developing PND. Unit costs were applied to estimates of health and social care resource use made by 206 women recruited from antenatal clinics and their infants. Net costs per mother-infant dyad over the first 18 months post-partum were estimated.<br><br>**Type of intervention [*exposure*]:** Preventative PND intervention.<br><br>**Data collection methods**: primiparous women attending antenatal clinics at 26–28 weeks of gestation were screened using a predictive index for PND. Women identified as being at high risk of developing PND were entered into an RCT of a preventive	**Sample size:** 206<br><br>**Participants:** Primiparous women at high risk of developing PND<br><br>**Setting:** antenatal clinics<br><br>**Dates of data collection:** May 1997 to April 1999	**Primary Findings:**<br>Mean mother-infant dyad costs were estimated at £2,419.00 for women with PND and £2026.90 for women without PND, a mean cost difference of £392.10 (P=0.17). The mean cost differences between women with and without PND reached statistical significance for community care services (P=0.01), but not for other categories of service. Economic costs were higher for women with extended experiences of the condition.	Aimed to estimate the economic costs of PND in a geographically defined cohort of women at high risk of developing the condition.

For peer review only - http://bmjopen.bmj.com/site/about/guidelines.xhtml

	intervention for PND delivered by trained health visitors. Economic data of women in the trial and in the observational study were pooled. An independent researcher assessed the mental state of all women at 8 weeks, 18 weeks, 12 months, and 18 months post-partum using the Structured Clinical Interview for DSM–III–R diagnoses (SCID–II).			
Petrou et al (2006)<br><br>(Petrou et al., 2006)<br><br>**UK**	**Study Design** A prospective economic evaluation was conducted alongside a pragmatic RCT<br><br>**Type of intervention [*exposure*]:** psychosocial and psychological interventions including counselling for the prevention of PND.<br><br>**Data collection methods:** Data on health and social care use by women and their infants up to 18 months postpartum were collected, using a combination of prospective diaries and face-to-face interviews	**Sample size:** 151 women<br><br>**Participants:** Women considered at high risk of developing PND were allocated randomly to the preventive intervention (*n* = 74) or to routine primary care (*n* = 77)<br><br>**Setting:** Health care setting.<br><br>**Dates of data collection:** c.2000	**Primary Findings:**<br>• Women in the preventive intervention group were depressed for an average of 2.21 months (9.57 weeks) during the study period, whereas women in the routine primary care group were depressed for an average of 2.70 months (11.71 weeks).<br>• The mean health and social care costs were estimated at £2,396.9 per mother–infant dyad in the preventive intervention group and £2,277.5 per mother–infant dyad in the routine primary care group, providing a mean cost difference of £119.5 (bootstrap 95 percent confidence interval [CI], −535.4, 784.9).<br>• At a willingness to pay threshold of £1,000 per month of PND avoided, the probability that the preventive intervention is cost-effective is .71 and the mean net benefit is £383.4 (bootstrap 95 percent CI, −£863.3–£1,581.5).<br><br>**Additional Findings:**<br>The preventive intervention is likely to be cost-effective even at relatively low willingness to pay thresholds for preventing 1 month of PND during the first 18 months postpartum. Given the negative impact of PND on later child development.	This cost-effectiveness analysis found that given the negative impact of PND on later child development, preventive interventions are likely to be cost-effective even at relatively low willingness to pay thresholds for preventing one month of PND during the first 18 months post-partum.
Roberts et al (2001)<br>[42]	**Study design:** Cross-sectional<br><br>**Data collection methods:** EPDS and the Health and Social Service Utilization Questionnaire (HSUQ)	**Sample size:** 1,250<br><br>**Participants:** mothers of infants.	**Primary findings:**<br>Costs were notably different for mothers with and without depression as determined by the EPDS (score of > 12). The total cost for health and social care $845 for mothers with	A cross-sectional study of 1250 mothers of infants in a Canadian setting used the EPDS to investigate the costs associated with perinatal depression. It was found that

For peer review only - http://bmjopen.bmj.com/site/about/guidelines.xhtml

**Canada**		**Setting:** Canadian healthcare setting<br><br>**Dates of data collection:** 1999	depression and their infant's vs $413 for those with lower scores. This was statistically significant difference at the (p < .01).<br>**Additional findings:**<br>Costs for social work visits were higher for mothers with depression and mothers with low incomes.<br>Total health and social care costs were double for mothers with family income below $20,000 ($788 v $399) and for mothers with clinical depression ($845 v $413). Nursing care costs were greater for mothers with high depression scores ($135 v $81).	costs were notably different for mothers with and without depression. The total cost for health and social care was $845 for mothers with depression and their infant's vs $413 for those with lower depression scores. This was statistically significant different at p < .01.
Stevenson et al (2010) (Stevenson et al., 2010)<br><br>UK	**Study Design:** cost-effectiveness analysis to assess group-CBT (gCBT) in comparison with routine primary care for women with PND in the UK.<br><br>**Type of intervention [*exposure*]:** Group-CBT<br><br>**Data collection methods:** SR	**Sample size:** 401<br><br>**Participants:** Data were analysed from 401 women with an EPDS score of 12 or greater at 6 weeks after childbirth, which had completed both the EPDS and the SF-6D questionnaire at both 6 weeks and 6 months<br><br>**Setting:** Postnatal healthcare setting in the UK<br><br>**Dates of data collection:** Pre-July 2009 (when PONDER study was published**).**	**Primary Findings:**<br>The mean cost per QALY from the stochastic analysis was estimated to be £36,062; however, there was considerable uncertainty around this value. The EVPI was estimated to be greater than £64 million; the key uncertainties were in the cost per woman of providing treatment and in the statistical relationship between changes in EPDS values and changes in SF-6D values. The expected value of perfect partial information for both of these parameters was more than £25 million.<br><br>**Additional Findings:**<br>The use of gCBT does not appear to be cost-effective; however, this decision is uncertain. The value of information analyses conducted indicates that further research to provide robust information on key parameters is needed and appears justified in cost-effective terms.	This economic evaluation found that gCBT does not appear to be cost-effective due to the lack of literature providing robust information. Only one study, an RCT, was deemed applicable to the decision problem.
Wilkinson et al (2017)<br><br>(Wilkinson et al., 2017)<br><br>**USA**	**Study Design:** Modelling study<br><br>**Type of intervention [*exposure*]:** N/A<br><br>**Data collection methods**: Hypothetical cohort	**Sample size:** 1,000<br><br>**Participants:** follows a hypothetical cohort of 1000 pregnant women experiencing one live birth over a 2-year time horizon.	**Primary Findings:**<br>• Screening for and treating postpartum depression and psychosis produced 29 more healthy women at a cost of $943 per woman.<br>• The incremental cost-effectiveness ratios of the intervention branch compared to usual care were $13,857 per QALY	This economic modelling study modelled the cost-effectiveness of physicians screening for and treating postpartum depression and psychosis in partnership with a psychiatrist.

For peer review only - http://bmjopen.bmj.com/site/about/guidelines.xhtml

		**Setting:** USA healthcare setting.<br><br>**Dates of data collection:** data were obtained from literature published between 1995 and 2015.	gained (below the commonly accepted willingness to pay threshold of $50,000/QALY gained) and $10,182 per remission achieved.<br>• These results were robust in both the deterministic and probabilistic sensitivity analyses of input parameters.<br><br>**Additional Findings:**<br>Screening for and treating postpartum depression is a cost-effective intervention and should be considered as part of usual postnatal care, which aligns with the recently proposed recommendations from the U.S. Preventive Services Task Force.	

For peer review only

For peer review only - http://bmjopen.bmj.com/site/about/guidelines.xhtml

Data extraction table for studies including maternal health and well-being

Citation (Country)	Study Details	Participants and setting	Key findings	Observations
Chojenta et al (2019)<br><br>(Chojenta et al., 2019)<br><br>Australia	**Study design:** Cross-sectional<br><br>**Data collection methods:** Health economics modelling study.<br><br>Data were taken from the Australian Longitudinal Study on Women's Health (ALSWH), an ongoing population-based study of health and well-being.	**Sample size:12,689**<br><br>**Participants:** Three cohorts of women born 1973–78, 1946–1951 and 1921–1926, with a fourth cohort born in 1989–1995 added in 2012.<br><br>**Setting:** Australian healthcare setting.<br><br>**Dates of data collection:** 1921 to 1995	**Primary findings:**<br>The healthcare costs for postnatal women who had poor mental health prior to birth was $1,792 (AUSD). This is on average 11% more than for mothers with no previous history of poor mental health.	This modelling study from Australia, utilising cohort data from 1921 to 1995 found that the healthcare costs for postnatal women who had poor mental health prior to birth was $1,792 (AUSD). This is on average 11% more than for mothers with no previous history of poor mental health.
Morrell et al (2000)<br><br>[34]<br><br>**UK**	**Study Design:** RCT<br><br>**Type of intervention [*exposure*]:**<br>Up to 10 home visits in the first postnatal month of up to three hours duration by a community postnatal support worker.<br><br>Impact of community postnatal support worker in addition to usual community midwife care on rest and recovery, health status, satisfaction with services and NHS Resource use and costs.<br><br>**Data collection methods:** Postal questionnaires (including SF36 and EPDS).	**Sample size:** 623<br><br>**Participants:** Postnatal women delivering at a university hospital<br><br>**Setting:** Home and community<br><br>**Dates of data collection:** Recruitment on labour wards from October 1996 to November 1997	**Primary Findings:** 551 completed 6 weeks questionnaire, 493 at 6 months. No evidence of use of fewer NHS services by women using the support worker versus controls at 6 weeks or 6 months.<br>Additional costs per woman at 6 weeks of £179.58 mostly due to support worker training (p<0.001).<br><br>**Additional Findings:** No diff primary outcome at 6 weeks but p<0.05 for physical and social functioning and p=005 EPDS for controls.<br>No difference in SF36 health status scores, EPDS scale or Duke Functional Social Support scale, rate of breastfeeding).	This study found that there were no savings to the NHS over six months after the introduction of a community support worker service and no improvement to the health status among the women in the intervention group, which was measured by an SF-36 questionnaire. At six weeks, the mean total NHS costs were £975□ for the intervention group and £700 for the control group. At six months, the figures were £1,250 and £980, respectively.
Ride (2018)	**Study Design:** Modelling study (health economics)	**Date of model:** 2018<br><br>The models were developed using TreeAge Pro 2015 software (TreeAge Software, Inc.,	**Primary Findings:** The results suggest that broader boundaries, particularly extension of the time horizon, could make substantial differences to	By ignoring broader sets of costs and outcomes, resources in postnatal mental health may be misallocated, and as a result, some women may not benefit as

For peer review only

(Ride, 2018)<br><br>**UK**	**Data collection methods:** Decision analytic modelling	Williamstown, MA, USA). The population of interest was postnatal women and their children in the United Kingdom, because much of the data came from that setting; this gave an explicit societal threshold of £20,000 to £30,000 per QALY for cost-effectiveness analysis in health care. A health sector perspective was taken, except for the children's model, which expanded to a public sector perspective to accommodate educational costs. A discount rate of 3.5% was applied to costs and QALYs, with discounting applied back to the child's birth. All costs were converted to 2014 pounds sterling.	estimated cost-effectiveness. Inclusion of family effects without extension of the time horizon had little impact, but where a longer time horizon was used, family effects could make a significant difference to the conclusions drawn from cost-effectiveness analysis<br><br>**Additional Findings:**<br>The authors note that it is important not only to consider caregiving but also family health effects in the outcomes of maternal health studies.	much from interventions that might be cost-effective given a broader time-horizon.

For peer review only

For peer review only - http://bmjopen.bmj.com/site/about/guidelines.xhtml

PRISMA 2020 Checklist

Section and Topic	Item #	Checklist item	Location where item is reported
**TITLE**			
Title	1	Identify the report as a systematic review.	Page 2
**ABSTRACT**			
Abstract	2	See the PRISMA 2020 for Abstracts checklist.	Figure 1
**INTRODUCTION**			
Rationale	3	Describe the rationale for the review in the context of existing knowledge.	Page 4
Objectives	4	Provide an explicit statement of the objective(s) or question(s) the review addresses.	Page 4
**METHODS**			
Eligibility criteria	5	Specify the inclusion and exclusion criteria for the review and how studies were grouped for the syntheses.	Page 5
Information sources	6	Specify all databases, registers, websites, organisations, reference lists and other sources searched or consulted to identify studies. Specify the date when each source was last searched or consulted.	Page 5
Search strategy	7	Present the full search strategies for all databases, registers and websites, including any filters and limits used.	Supplementary Material
Selection process	8	Specify the methods used to decide whether a study met the inclusion criteria of the review, including how many reviewers screened each record and each report retrieved, whether they worked independently, and if applicable, details of automation tools used in the process.	Page 6
Data collection process	9	Specify the methods used to collect data from reports, including how many reviewers collected data from each report, whether they worked independently, any processes for obtaining or confirming data from study investigators, and if applicable, details of automation tools used in the process.	Page 6
Data items	10a	List and define all outcomes for which data were sought. Specify whether all results that were compatible with each outcome domain in each study were sought (e.g. for all measures, time points, analyses), and if not, the methods used to decide which results to collect.	Page 6
	10b	List and define all other variables for which data were sought (e.g. participant and intervention characteristics, funding sources). Describe any assumptions made about any missing or unclear information.	Page 6
Study risk of bias assessment	11	Specify the methods used to assess risk of bias in the included studies, including details of the tool(s) used, how many reviewers assessed each study and whether they worked independently, and if applicable, details of automation tools used in the process.	Supplementary Material
Effect measures	12	Specify for each outcome the effect measure(s) (e.g. risk ratio, mean difference) used in the synthesis or presentation of results.	Page 8
Synthesis methods	13a	Describe the processes used to decide which studies were eligible for each synthesis (e.g. tabulating the study intervention characteristics and comparing against the planned groups for each synthesis (item #5)).	Page 6
	13b	Describe any methods required to prepare the data for presentation or synthesis, such as handling of missing summary statistics, or data conversions.	Page 6
	13c	Describe any methods used to tabulate or visually display results of individual studies and syntheses.	From Page 9
	13d	Describe any methods used to synthesize results and provide a rationale for the choice(s). If meta-analysis was performed, describe the model(s), method(s) to identify the presence and extent of statistical heterogeneity, and software package(s) used.	Page 8
	13e	Describe any methods used to explore possible causes of heterogeneity among study results (e.g. subgroup analysis, meta-regression).	N/A
	13f	Describe any sensitivity analyses conducted to assess robustness of the synthesized results.	N/A
Reporting bias assessment	14	Describe any methods used to assess risk of bias due to missing results in a synthesis (arising from reporting biases).	Supplementary Material
Certainty	15	Describe any methods used to assess certainty (or confidence) in the body of evidence for an outcome.	

For peer review only - http://bmjopen.bmj.com/site/about/guidelines.xhtml

PRISMA 2020 Checklist

Section and Topic	Item #	Checklist item	Location where item is reported
assessment			
**RESULTS**			
Study selection	16a	Describe the results of the search and selection process, from the number of records identified in the search to the number of studies included in the review, ideally using a flow diagram.	Page 8
	16b	Cite studies that might appear to meet the inclusion criteria, but which were excluded, and explain why they were excluded.	Page 8
Study characteristics	17	Cite each included study and present its characteristics.	Page 8
Risk of bias in studies	18	Present assessments of risk of bias for each included study.	Page 8
Results of individual studies	19	For all outcomes, present, for each study: (a) summary statistics for each group (where appropriate) and (b) an effect estimate and its precision (e.g. confidence/credible interval), ideally using structured tables or plots.	Page 8
Results of syntheses	20a	For each synthesis, briefly summarise the characteristics and risk of bias among contributing studies.	Page 8
	20b	Present results of all statistical syntheses conducted. If meta-analysis was done, present for each the summary estimate and its precision (e.g. confidence/credible interval) and measures of statistical heterogeneity. If comparing groups, describe the direction of the effect.	Page 8
	20c	Present results of all investigations of possible causes of heterogeneity among study results.	Page 8
	20d	Present results of all sensitivity analyses conducted to assess the robustness of the synthesized results.	N/A
Reporting biases	21	Present assessments of risk of bias due to missing results (arising from reporting biases) for each synthesis assessed.	Supplementary Material
Certainty of evidence	22	Present assessments of certainty (or confidence) in the body of evidence for each outcome assessed.	Supplementary Material
**DISCUSSION**			
Discussion	23a	Provide a general interpretation of the results in the context of other evidence.	Page 25
	23b	Discuss any limitations of the evidence included in the review.	Page 2
	23c	Discuss any limitations of the review processes used.	N/A
	23d	Discuss implications of the results for practice, policy, and future research.	Page 26
**OTHER INFORMATION**			
Registration and protocol	24a	Provide registration information for the review, including register name and registration number, or state that the review was not registered.	Page 5
	24b	Indicate where the review protocol can be accessed, or state that a protocol was not prepared.	Page 5
	24c	Describe and explain any amendments to information provided at registration or in the protocol.	N/A
Support	25	Describe sources of financial or non-financial support for the review, and the role of the funders or sponsors in the review.	Page 27
Competing interests	26	Declare any competing interests of review authors.	Page 27
Availability of data, code and other materials	27	Report which of the following are publicly available and where they can be found: template data collection forms; data extracted from included studies; data used for all analyses; analytic code; any other materials used in the review.	Page 9 onwards

For peer review only

PRISMA 2020 Checklist

From: Page MJ, McKenzie JE, Bossuyt PM, Boutron I, Hoffmann TC, Mulrow CD, et al. The PRISMA 2020 statement: an updated guideline for reporting systematic reviews. BMJ 2021;372:n71. doi: 10.1136/bmj.n71
For more information, visit: http://www.prisma-statement.org/

For peer review only

BMJ Open

Health economic evaluations of preventative care for perinatal anxiety and associated disorders: A rapid review

Journal:	*BMJ Open*
Manuscript ID	bmjopen-2022-068941.R2
Article Type:	Original research
Date Submitted by the Author:	22-Nov-2023
Complete List of Authors:	Pisavadia, Kalpa; Bangor University, School of Medical and Health Sciences<br>Spencer, Llinos; Bangor University, Centre for Health Economics and Medicine Evaluation<br>Tuersley, Lorna; Bangor University,<br>Coates, Rose ; City University of London,<br>Ayers, Susan; City University,<br>Edwards, Rhiannon; Bangor University, Centre for Health Economics & Medicines Evaluation
**Primary Subject Heading**:	Health economics
Secondary Subject Heading:	Health economics
Keywords:	HEALTH ECONOMICS, PREVENTIVE MEDICINE, MENTAL HEALTH, Postpartum Women < Postpartum Period

SCHOLARONE™
Manuscripts

I, the Submitting Author has the right to grant and does grant on behalf of all authors of the Work (as defined in the below author licence), an exclusive licence and/or a non-exclusive licence for contributions from authors who are: i) UK Crown employees; ii) where BMJ has agreed a CC-BY licence shall apply, and/or iii) in accordance with the terms applicable for US Federal Government officers or employees acting as part of their official duties; on a worldwide, perpetual, irrevocable, royalty-free basis to BMJ Publishing Group Ltd ("BMJ") its licensees and where the relevant Journal is co-owned by BMJ to the co-owners of the Journal, to publish the Work in this journal and any other BMJ products and to exploit all rights, as set out in our licence.

The Submitting Author accepts and understands that any supply made under these terms is made by BMJ to the Submitting Author unless you are acting as an employee on behalf of your employer or a postgraduate student of an affiliated institution which is paying any applicable article publishing charge ("APC") for Open Access articles. Where the Submitting Author wishes to make the Work available on an Open Access basis (and intends to pay the relevant APC), the terms of reuse of such Open Access shall be governed by a Creative Commons licence – details of these licences and which Creative Commons licence will apply to this Work are set out in our licence referred to above.

Other than as permitted in any relevant BMJ Author's Self Archiving Policies, I confirm this Work has not been accepted for publication elsewhere, is not being considered for publication elsewhere and does not duplicate material already published. I confirm all authors consent to publication of this Work and authorise the granting of this licence.

For peer review only

Health economic evaluations of preventative care for perinatal anxiety and associated disorders: A rapid review

Miss Kalpa Pisavadia[1] Kalpa.pisavadia@bangor.ac.uk

Dr Llinos Haf Spencer[1] L.spencer@bangor.ac.uk

Dr Lorna Tuersley[1] Lorna.tuersley@gmail.com

Dr Rose Coates[2] Rose.coates@city.ac.uk

Professor Susan Ayers[2] Susan.ayers.1@city.ac.uk

Professor Rhiannon Tudor Edwards[1] R.t.edwards@bangor.ac.uk

Corresponding author: Kalpa Pisavadia Research Project Support Officer, Centre for Health Economics and Medicines Evaluation (CHEME), Ardudwy Hall, Normal Site, Bangor University, Bangor, Gwynedd, Wales, UK, LL57 2PZ. Email: Kalpa.pisavadia@bangor.ac.uk

For peer review only - http://bmjopen.bmj.com/site/about/guidelines.xhtml

Abstract

Objectives: Perinatal mental health (PMH) problems affect one in five women and cost the United Kingdom (UK) £8.1 billion for every year of births, with 72% of this cost due to the long-term impact on the child. We conducted a rapid review of health economic evaluations of preventative care for perinatal anxiety and associated disorders.

Design: This study adopted a rapid review approach, using principles of the standard systematic review process to generate quality evidence. This methodology features a systematic database search, PRISMA diagram, screening of evidence, data extraction, critical appraisal, and narrative synthesis.

Data Sources: PubMed, Cumulative Index to Nursing and Allied Health Literature (CINAHL), Cochrane Library, *Applied Social Sciences Index and Abstracts* (ASSIA), PsycINFO and MEDLINE.

Eligibility criteria for selecting studies: Studies that evaluated the costs and cost-effectiveness of preventative care for perinatal anxiety and associated disorders carried out within the National Health Service (NHS) and similar healthcare systems.
Data extraction and synthesis: A minimum of two independent reviewers used standardised methods to search, screen, critically appraise and synthesise included studies.

Results: The results indicate a lack of economic evaluation specifically for perinatal anxiety, with most studies focussing on postnatal depression (PND). Interventions to prevent postnatal mental health problems are cost-effective. Modelling studies have also been conducted, which suggest that treating PND with counselling would be cost-effective.

Conclusion: The costs of not intervening in maternal mental health outweigh the costs of preventative interventions. Preventative measures such as screening and counselling for maternal mental health are shown to be cost-effective interventions to improve outcomes for women and children.

Key words: preventative, life-course, perinatal anxiety, postnatal depression, cost of illness, cost-effectiveness, economic modelling.

PROSPERO registration number: CRD42022347859

For peer review only

Article summary

Strengths and limitations of this study

- The strength of this rapid review is that it has highlighted costs associated with perinatal mental health interventions in a rigorous, novel way and has identified several gaps for future research.
- The absence of health economic studies describing the range of public sector costs and costs to individuals from Scotland and Wales in relation to perinatal anxiety is a limitation of this rapid review.
- Although health economic studies are showing the benefits of investing in PND, there are no published UK-based Randomised controlled Trials (RCT) investigating perinatal mental health interventions, which include information on costs, is a limitation of this rapid review.

Introduction

The perinatal period refers to pregnancy and the first 12 months after childbirth [1]. One in five women experience mental health problems during this time, and the cost is estimated to be £8.1 billion for every year of births in the United Kingdom (UK) [2] (see supplementary file 1 for a list of abbreviations). Maternal mental health problems include postnatal depression (PND) (also known as Postpartum Depression (PPD) internationally), characterised by depressed mood and anxiety, feelings of inadequacy, and impaired infant bonding [3].

Untreated maternal mental illness not only impacts mothers, but also adversely impacts their children, significantly contributing to wider societal and National Health Service (NHS) costs. Of the total costs of perinatal mental health difficulties in the UK, 72% is due to the long-term impact on the child [2]. Decreased maternal and infant bonding, reduced breastfeeding initiation rates and duration, low birth weight, and poorer child growth have been associated with PND [4]. Children of mothers experiencing sub-clinical and persistently high depressive symptoms were twice as likely to have emotional and behavioural difficulties than children of mothers reporting minimal symptoms [5]. Delayed or impaired cognitive, linguistic, physical, and psychological health development has been reported in infants and children with mothers with PND [4]. There is also a risk of intergenerational transmission of socio-economic disadvantage in which maternal mental illness impacts the child's quality of life by having a long-term adverse effect on education and employment prospects [6,7]. Public sector costs are likely to be significantly reduced by utilising a prevention strategy to reduce the incidence of poor maternal mental health [7].

Despite the long-term risks of untreated maternal mental health issues, as of 2014, only 30-50% of women with PMH problems were identified, and only 7% were referred to specialist care in the UK. [2]. Most women with PMH problems did not access care [2]. This may have been particularly the case for women with mild to moderate PMH problems or less commonly recognised problems, such as anxiety, obsessive-compulsive disorder (OCD), or post-traumatic stress disorder (PTSD) [2].

For peer review only - http://bmjopen.bmj.com/site/about/guidelines.xhtml

Access to care may also be limited by maternal time constraints and fears of being judged [8].

The National Institute for Health and Care Excellence recommend postnatal care for up to eight weeks after birth [9]. Since 2015, it has been recommended that UK midwives carry out emotional well-being checks at antenatal check-ups and at each postnatal contact up to eight weeks after birth. In 2018, the National Collaborating Centre for Mental Health worked with NICE to develop the Perinatal Mental Health Care Pathway [10]. The guidance in that report follows a process agreed upon by NICE and sets out pathways to deliver a strategic transformation of perinatal mental health care. Psychological interventions, either alone or in conjunction with pharmacological treatment, are recommended for complex or severe mental health problems following referral to a specialist community perinatal mental health team [1].

Since 2015, there have been improvements to funding plans and commitments in the provision of more specialist Community Perinatal Mental Health Services across the UK. For example, in 2019, the Scottish Government revealed that £52 million would be spent on improving access to perinatal and infant mental health services, and from 2018 to 2020, the Welsh Government increased recurrent annual funding from £1.5 million to £2.5 million for specialist PMH services [11]. In England, the Government committed £365 million to provide specialist perinatal community services across the country, as announced by NHS England in April 2019 [9]. It is, imperative that proactive planning and cost-effective preventative solutions are a public policy priority [6].

Aim

This review aims to investigate the type of health economic evaluations of preventative care for perinatal anxiety and associated disorders carried out within the National Health Service (NHS) and similar healthcare systems.

Methods

This review utilised principles from the standard systematic review process to generate quality evidence in a shorter time frame. This methodology included a systematic database search, PRISMA diagram [12] (see figure 1) screening of evidence, data extraction, critical appraisal, and narrative synthesis. This revised methodology is used by the Health and Care Research Wales Evidence Centre [13–15]. Cost-effectiveness outcomes are reported according to The Professional Society for Health Economics and Outcomes Research (ISPOR) guidelines [16].

Patient and Public Involvement

None

For peer review only - http://bmjopen.bmj.com/site/about/guidelines.xhtml

Search Strategy

The key evidence sources included PubMed, Cumulative Index to Nursing and Allied Health Literature (CINAHL), Cochrane Library, Applied Social Sciences Index and Abstracts (ASSIA), PsycINFO and MEDLINE. The search terms consisted of words related to perinatal anxiety and/or depression, health and psychiatric services and economic evaluation terms. The searches were conducted on 23 April 2022. Mendeley reference management software was used to manage study articles found and remove duplicates. See supplementary file 1 for the full search strategy.

The eligibility criteria for the review are presented in Table 1 and are based on the Population, Intervention, Comparison and Outcome (PICO) framework [17]. This consisted of peer-reviewed economic evaluations of perinatal anxiety and associated disorders such as PND and PTSD from Organisation for Economic Co-operation and Development (OECD) countries in English published after January 2000.

Table 1: Participants, Intervention/exposure, Comparator and Outcomes (PICO) framework

**Question**	
What is the cost of care for women experiencing perinatal anxiety and associated disorders?	
**Participants**	Pregnant women or perinatal women
**Intervention / exposure**	Perinatal anxiety and associated disorders
**Comparator**	No comparator
**Outcomes**	Costs of primary care and support services for women experiencing perinatal anxiety and associated disorders
**Study Considerations**	
Primary research, secondary research, grey literature, and preprints	
**Databases**	
PubMed, CINAHL, Cochrane Library, ASSIA, PsycINFO, and MEDLINE	

Selection of studies

One reviewer (KP) independently selected potentially eligible studies based on a screening of titles and abstracts. Two reviewers (LHS and KP) selected additional studies from existing systematic reviews. The full texts of selected studies were assessed for eligibility by three reviewers (KP and LHS, with mediation by LT) in the data extraction process.

For peer review only - http://bmjopen.bmj.com/site/about/guidelines.xhtml

Data extraction

Data extraction and study quality assessment were performed by three reviewers (KP, LHS, LT). Data was collected on country, study design, intervention type, data collection methods and dates, sample size, and type of participants (See supplementary file 2 for data extraction tables).

Quality assessment

The quality assessment was undertaken by two reviewers (LHS and KP), and four papers were checked by a third reviewer for quality assurance purposes (LT). The Drummond checklist [18] was used for the quality appraisal of health economic papers, and the checklist for critical appraisal and data extraction for systematic reviews of prediction modelling studies (CHARMS) checklist was used for the modelling studies [19]. The Joanna Briggs Institute (JBI) critical appraisal tools were used for the quality appraisal, randomised clinical trials, cohort studies and cross-sectional studies [20–22] (see supplementary file 1).

[Insert figure 1 here]

For peer review only

Results

Searches of databases yielded 3212 results, of which 1226 duplicates were removed. The remaining 1986 results were screened against titles and abstracts, and an additional four papers were retrieved from existing systematic reviews. A total of 17 papers met the criteria for full-text screening. Eleven papers were excluded due to not being able to access the full text (n=4), ineligible study design (n=5), or lack of relevancy (non-OECD country) (n=2). Seventeen studies were included in this rapid review (see Figure 1 and Table 2).

Of these seventeen included papers, there were cost-effectiveness studies (n=5), modelling studies (n=6), cost-benefit study (n=1), a cost analysis study (n=1) and cost of illness studies (n=4). All included studies were peer-reviewed. The included studies were categorised according to main intervention: children, prevention, cost of maternal health, cost of single interventions, and comparison cost of interventions. The following discussion provides a more detailed overview of the findings.

Table 2: Map of maternal cost of illness studies by evidence type (including studies on depression, anxiety and maternal health and well-being)

**Type of Evidence**	**Type of intervention**					
	**Children**	**Prevention**	**Cost of maternal health**	**Cost of single interventions**	**Comparison cost of interventions**	**Number of studies**
**Cost-effectiveness**		Petrou et al. (2006) [3]		Morrell et al. (2000) [23]	Henderson et al. (2019) [24]	**5**
		Ride et al. (2016) [25]		Stevenson et al. (2010) [26]		
**Cost-benefit**					Grote et al. (2017) [27]	**1**
**Cost-analysis**	Moore Simas et al. (2020) [4]					**1**
**Cost-of-illness**			Petrou et al. (2002) [28]			**4**
			Dagher et al. (2012) [29]			
			Ammerman et al. (2016) [30]			
			Roberts et al, (2001) [31]			
**Economic modelling studies**	Bauer et al. (2015) [6]	Counts et al. (2022) [32,33]	Franta et al. (2022) [33]			**6**
	Ride (2018) [34]	Wilkinson et al. (2017) [35]	Chojenta et al. (2019) [36]			
**Total number of studies**	**3**	**4**	**6**	**2**	**2**	**17**

For peer review only - http://bmjopen.bmj.com/site/about/guidelines.xhtml

Table 3: Methodological considerations and cost-effectiveness results

Lead author (Year)	Intervention	Perspective (reasons)	Time horizon used in economic evaluation (reasons)	Discounting	Key cost-effectiveness results
Henderson et al (2019) [24]	**Intervention group:** PoNDER: Health visitor (HV) training to assess postnatal depression (PND) and deliver psychological approaches to women at risk of depression.<br>**Control group:** Usual care	NHS and social care perspective.	Resource use data from 6 weeks to 6 months were collected on a resource use log completed by HVs based on their own and GP records	No discounting was necessary due to the duration of the follow-up period.	Costs and outcomes data were available for 1459 participants. 6-month adjusted costs were £82 lower in intervention than control groups, with 0.002 additional QALY gained. The probability of cost-effectiveness at £20,000 was very high (99%).
Morrell et al (2000) [23]	**Intervention group:** up to 10 home visits in the first postnatal month of up to three hours duration by a community postnatal support worker.<br>**Control group:** Usual care	NHS perspective	Up to 10 home visits in the first postnatal month of up to three hours duration by a community postnatal support worker, and a 6-month follow-up.	No	Cost data showed that at six weeks the mean total NHS costs were £635 for the intervention group and £456 for the control group (P = 0.001). At six months figures were £815 and £639 (P = 0.001).<br><br>However, due to there being no differences between the groups in use of social services or personal costs, no cost-effectiveness analysis was conducted.
Petrou et al (2006) [3]	**Intervention group:** counselling and specific support for the mother relationship, targeted at women at high risk of developing postnatal depression.<br>**Control group:** Usual care	The economic evaluation was conducted from a public sector perspective.	The time horizon for the economic evaluation mirrored the time horizon for the randomized controlled trial, namely the period between randomization and 18 months postpartum.	Various discounting rates were applied as necessary: 0 percent, 1.5 percent, 3 percent, 6 percent, and 10 percent.	The mean health and social care costs were estimated at £2,396.9 per mother-infant dyad in the preventive intervention group and £2,277.5 per mother-infant dyad in the routine primary care group, providing a mean cost difference of £119.5 (bootstrap 95 percent confidence interval [CI], -535.4, 784.9). At a willingness to pay threshold of £1,000 per month of postnatal depression avoided, the probability that the preventive intervention is cost-effective is.71 and the mean net benefit is £383.4 (bootstrap 95 percent CI, -£863.3-£1,581.5).
Ride et al (2016) [25]	Intervention group: What Were We Thinking (WWWT) - a psychoeducational	A range of perspectives including patient, NHS, and	The time horizon of 6 months mirrored the	No discounting was necessary due to the	The incremental cost-effectiveness ratios were $A36 451 per QALY gained and $A152 per percentage point

For peer review only - http://bmjopen.bmj.com/site/about/guidelines.xhtml

	intervention targeted at the partner relationship, management of infant behaviour and parental fatigue. **Control group:** Usual care	social services.	trial follow-up period. No	duration of the follow-up period.	reduction in 30-day prevalence of depression, anxiety, and adjustment disorders. The estimate lies under the unofficial cost-effectiveness threshold of $A55 000 per QALY; however, there was considerable uncertainty surrounding the results, with a 55% probability that WWWT would be considered cost-effective at that threshold.
Stevenson et al (2010) [26]	**Intervention group:** Cognitive Behaviour Therapy (gCBT). **Control group:** Usual care	Health sector perspective	Treatment up to 8 weeks, and a 6-month follow-up.	No discounting was necessary due to the duration of the follow-up period.	The use of gCBT does not appear to be cost-effective.<br><br>The mean cost per quality adjusted life year (QALY) from the stochastic analysis was estimated to be £36,062; however, there was considerable uncertainty around this value. The expected value of perfect information (EVPI) was estimated to be greater than £64 million; the key uncertainties were in the cost per woman of providing treatment and in the statistical relationship between changes in the Edinburgh Postnatal Depression Scale (EPDS) values and changes in the Short Form – 6 Dimensions (SF-6D) values. The expected value of perfect partial information for both of these parameters was in excess of £25 million.

The included papers are organised under three different themes: perinatal anxiety, perinatal depression, and perinatal health and well-being. These studies are detailed below, and all non-UK prices have been converted to pound sterling currency and inflated to the latest available prices [37–41].

Summary of studies including perinatal anxiety

This review found one economic evaluation focussing on perinatal anxiety [25,42]. This study consisted of a cost-effectiveness, cost-utility analysis and cluster-

For peer review only - http://bmjopen.bmj.com/site/about/guidelines.xhtml

randomised controlled trial of the What Were We Thinking (WWWT) intervention [25]. WWWT is a psychoeducational intervention targeted at the partner relationship, management of infant behaviour and parental fatigue for the prevention of postnatal maternal mental health problems (See Table 3 for further details). There were no statistically significant differences in either costs or effectiveness.

Summary of studies including perinatal depression

Fifteen studies focussed on perinatal depression [3,4,6,26–33,35,43–45]. A cross-sectional study from the USA conducted between 2006 and 2011 investigated the out-of-pocket expenses and insurer expenses of depressed mothers compared to non-depressed mothers [30]. Depressed mothers were more likely to incur insurer out-of-pocket expenses (£1,285 vs £853 □□) and have higher insurer expenses (£10,485 vs £7,508□□).

One Study used the perspective of the public sector, individuals, and society to examine some of the outcomes and long-term economic implications experienced by offspring who have been exposed to perinatal depression in a South London cohort [6]. Bauer et al. (2015) found that for each child exposed to perinatal depression, public sector costs exceeded £3,380□, costs due to reduced earnings were £1,562□, and health-related quality of life loss was valued at £3760□.

A decision analytic model used a simulated cohort of 1,000 Medicaid-enrolled pregnant individuals to evaluate the health care costs for individuals receiving PND preventive intervention or not, for 1 to 5 years post-partum [32]. This study found that providing preventive interventions for PPD resulted in an estimated 5-year saving of £602□□.

A cross-sectional study in the USA which investigated expenditure on healthcare services from hospital discharge until 11 weeks postpartum [29]. There was a significant difference in healthcare expenditure between depressed and non-depressed women. The Edinburgh Postnatal Depression Scale (EPDS) was used to measure depression [46]. The total cost of all mental health counselling visits for the depressed group (n=31) was £165□□, and the cost for the non-depressed group (n= 607) was £15.50□□ (in 2007). This was a statistically significant difference ($p < 0.001$).

Using a theoretical cohort of 180,000 individuals, a decision-analytic model compared outcomes in pregnant adolescents who received counselling interventions versus those who did not [33]. This study found that it is cost-effective to refer all pregnant adolescents for preventive counselling interventions. Within the theoretical cohort for counselling, there were 8,935 fewer cases of PND, 1,606 fewer cases of chronic depression, 166 fewer preterm deliveries, four fewer neonatal deaths, 20 fewer cases of sudden infant death syndrome (SIDS), and one fewer case of cerebral palsy. In total, there were 21,976 additional QALYs and cost savings of £183,463,169⅄, making it the dominant strategy that had better outcomes with lower costs.

An RCT trial compared a multicomponent collaborative care intervention for depression (MOMcare - a choice of brief interpersonal psychotherapy or pharmacotherapy or both) with enhanced maternity support services (MSS-Plus) in

For peer review only
For peer review only - http://bmjopen.bmj.com/site/about/guidelines.xhtml

the public health system of Seattle, USA [27]. The incremental benefit and cost and the net benefit for women with major depression and PTSD were estimated. When controlled for baseline depression severity, women with probable depression and PTSD in MOMCare had 68 more depression-free days over 18 months than those in MSS-Plus ($p<.05$). There was an additional £1,943□□ depression care cost per MOMCare participant with comorbid PTSD. The incremental net benefit of MOMCare was positive if depression free days were valued below £18□□. For women with probable major depression and PTSD, MOMCare had a significant clinical benefit over MSS-Plus, with only a moderate increase in health services cost.

A cluster RCT of health visitors trained to assess PND and deliver psychological approaches to women at risk of depression plus either a cognitive behavioural approach or a person-centred approach weekly for eight weeks was conducted in 2019 [24]. A cost-effectiveness analysis was run parallel to this for all mothers at low-risk of depression in accordance with the EPDS at six months postnatal. This study found that CBT had a marginally higher probability of being cost-effective than a person-centred approach. The short time horizon of 6 months postnatally means that the risks of long-term adverse effects were not factored into the analysis.

A cohort study with a sample size of 135,678 mother-child pairs with and without PND exposure revealed similar findings [4]. The results of this analysis suggest that the health resource utilisation and costs over the first 24 months of life in children of mothers with PND exceeded that of children of mothers without evidence of PND £22,940□□ and £20,487□□, respectively. This was a significant difference of £2,453.

A longitudinal study (18 months) conducted in 2002 estimated the economic costs of PND in cohort of women at high-risk of developing the condition with the use of an RCT to identify women considered to be of high-risk [28]. Unit costs were applied to estimates of health and social care resource use made by 206 women and their infants recruited from antenatal clinics, and net costs per mother-infant dyad over the first 18 months post-partum were estimated. This study found that costs were £587□ higher for women with PND than for women without PND. Economic costs were particularly higher for women with extended experiences of the condition.

A cost-effectiveness analysis of preventive interventions, consisting of counselling and support for the mother–infant relationship at high-risk of developing PND, was conducted in 2006 [3]. This study found that given the negative impact of PND on later child development, preventive interventions are likely to be cost-effective even at relatively low willingness to pay thresholds for preventing one month of PND during the first 18 months post-partum. The mean health and social care costs were estimated at £3,345□ per mother–infant dyad in the preventive intervention group and £3,277□ per mother–infant dyad in the routine primary care group, providing a mean cost difference of £166□.

A cross-sectional study of 1,250 mothers of infants in a Canadian setting used the EPDS to investigate the costs associated with perinatal depression [31]. It was found that costs were notably different for mothers with and without depression. The total cost for health and social care was £833□□ for mothers with depression and their

For peer review only - http://bmjopen.bmj.com/site/about/guidelines.xhtml

infants, compared to £406□□ for those with lower depression scores. This was statistically a significant difference at $p < .01$.

An economic evaluation conducted in 2010 compared the cost-effectiveness of group Cognitive Behavioural Therapy (gCBT) compared with routine primary care for women with PND in the UK [26]. This economic evaluation found that gCBT does not appear to be cost-effective due to the lack of literature providing robust information. Only one study, an RCT, was deemed applicable to the decision problem.

A cost-effectiveness analysis found that screening for and treating post-partum depression is a cost-effective intervention [35]. This study followed a hypothetical cohort of 1,000 pregnant women experiencing one live birth over a 2-year time horizon. The analysis found that screening for and treating PND and psychosis produced 29 more healthy women at the cost of £938□□ per woman. The incremental cost-effectiveness ratios (ICERs) of the intervention branch compared to usual care were £13,702□□ per quality-adjusted life year (QALY) gained (below the commonly accepted willingness to pay threshold of $50,000/QALY gained) and $10,182 per remission achieved.

Summary of studies including maternal health and well-being

This review found four studies relating to the health and well-being of perinatal women [23,34,36,47]. An RCT conducted in 2000 aimed to establish the relative cost-effectiveness of postnatal support in the community in addition to the usual care provided by the community midwives [23]. Three hundred and eleven women were allocated to the intervention of up to ten home visits by a community postnatal support worker. No health benefit was found for additional home visits by community postnatal support workers compared with traditional community midwifery visiting, as measured by the Short Form 36 measure. At six months, there was no significant improvement in health status among the women in the intervention group despite there being a significant difference in costs of £1,250□ (intervention group) and £980□ (usual care group), ($P = 0.001$). Although there were no savings to the NHS over six months after the introduction of the community postnatal support worker service, the women in the intervention group were very satisfied with the support worker visits.

Authors have suggested that prenatal interventions that do not seem cost-effective in the short term may be cost-effective over a longer time horizon [48]. A decision analytic modelling study noted that it is important to consider caregiving and family health effects in the outcomes of maternal health studies [34]. By not including broader sets of costs and outcomes, resources in postnatal mental health may be misallocated. As a result, some women may not benefit as much from interventions that might be cost-effective given a broader time horizon. The uncertainty surrounding the results in the decision analytic model may reflect decisions and investment in PND interventions.

A modelling study from Australia, published in 2019, utilised cohort data from 1921 to 1995 and found that the healthcare costs for postnatal women who had poor mental health prior to birth were £1,066˄ [36]. This is, on average, 11% more than for mothers with no previous history of poor mental health. These figures do not include out-of-pocket expenditure for the women who may have also purchased their own

For peer review only - http://bmjopen.bmj.com/site/about/guidelines.xhtml

over-the-counter medications and had other patient expenses which were not captured in the analysis.

Discussion

The aim of this review was to investigate the type of health economic evaluations of preventative care for perinatal anxiety and associated disorders carried out within the National Health Service (NHS) and similar healthcare systems. Twenty-one papers were included in this review from Australia, Canada, Ireland, the USA, and the UK, each examining maternal mental health.

The results indicate a lack of economic evaluation specifically for perinatal anxiety, with most study articles focusing on PND [26]. Only two included papers focussed on anxiety, with one being a systematic review looking at anxiety alongside depression [42]. The other was an economic evaluation of a maternal mental health intervention. Treatments for maternal mental health in the WWWT intervention consisted of health visitors with psychiatric training and group sessions focusing on parenting confidence and emotional well-being with online and face-to-face components [25]. The WWWT intervention shows promise as a preventive intervention, but uncertainty surrounding cost-effectiveness. The analysis showed no statistically significant difference in costs or outcomes between the intervention and control groups, with the intervention estimated to cost £74.48 per participant.

Most of the studies included (n=15 of the 17 included studies) focussed on the cost of services and interventions for PND. The evidence suggests significant health resource costs outside of mental health services as well as social care costs for PND for mother and mother-infant dyad. Costs were significantly higher for children of mothers with PND than for children of mothers without PND. This was a statistically significant difference of £2,453 (p <.001) [4].

Counselling was found to be a cost-effective, preventative intervention for pregnant adolescents [33]. Using a hypothetical cohort, one study found that counselling was a cost-effective preventative measure, leading to fewer cases of perinatal and chronic depression [33]. Another study estimated that group counselling (costing £114 per mother) cost around £73 less than individual counselling (£187 per mother) for mothers with PND [32]. This study found that screening for PND costs less than £2 per mother [32]. Studies that combined screening for PND with an intervention were also found to be cost-effective, resulting in 29 more healthy women at a cost of £938 per woman [35]. The incremental cost-effectiveness ratios of the intervention branch compared to usual care were $13,857 per QALY gained (below the commonly accepted willingness to pay threshold of $50,000/QALY gained) and $10,182 per remission achieved.

Within this review, the EPDS, a validated measure for postnatal depression and anxiety [46], was the most frequently used instrument to detect perinatal and PND in the included studies, followed by the SF-36 scale, postal questionnaires such as the Ontario health survey, Health and Social Service Utilisation Questionnaire (HSUQ), blinded telephone assessments and medical records, Medicaid data, resource use

For peer review only - http://bmjopen.bmj.com/site/about/guidelines.xhtml

logs completed by health visitors based on GP records, and prospective diaries and face-to-face interviews.

In summary, screening was found to be a relatively low-cost method of identifying women in need of mental health support during the perinatal period. Interventions to prevent postnatal mental health problems were found to be cost-effective [25]. Also, two modelling studies found that treating PND with counselling would be cost-effective [26,35].

Future research in this area should investigate how best to screen all mothers to prevent and treat further adverse outcomes such as anxiety, OCD, or PTSD [2]. Various psycho-social methods could be used to screen and provide treatment over the telephone, online or face-to-face. Interventions could be provided by a range of healthcare professionals, such as midwives, health visitors, counsellors, psychologists, and psychiatrists. The effectiveness and cost-effectiveness of each intervention, including screening, should be evaluated.

Web-based approaches are already promising to be cost-effective solutions to support mothers in the perinatal period. A recent cost-effectiveness study alongside an RCT in Singapore evaluated a web-based approach for delivering a psychoeducational intervention [14]. This web-based approach was cost-effective in supporting first-time mothers and provided the best improvements in self-efficacy, social support, and psychological well-being of mothers in the perinatal period. Most women of childbearing age, including women who reside in rural areas, now have access to the internet in the UK and similar health care systems. Being able to access support and treatment using online resources has widened access to care to postnatal care support.

Limitations of this study

Although this study conducted a thorough systematic search, only peer-reviewed literature was included. Relevant grey literature in this area may provide more insight into preventative interventions for maternal mental health that could be cost-effective. The findings in this area are limited by the literature available, particularly the absence of published RCTs with cost data, which would provide a rigorous method of hypothesis testing of perinatal mental health interventions.

Conclusion

This review demonstrated that very few economic evaluations have focussed on perinatal anxiety, and those which reported on cost of perinatal depression had short time horizons which did not allow for long-term outcomes for the mother and child dyad to be addressed. However, there was some evidence that preventative measures, such as postnatal depression screening, combined with treatment, such as counselling for maternal mental health, are proven to be effective interventions to improve outcomes for women and children.

For peer review only - http://bmjopen.bmj.com/site/about/guidelines.xhtml

Recommendations

It is recommended that:

- Mothers should be screened for maternal mental health issues to identify mothers at risk and provide treatment, leading to better outcomes for the mother and child dyad.
- Studies focussing on interventions for perinatal anxiety as a distinct condition to other mental health issues such as depression should be conducted.
- Cost of intervention studies related to perinatal anxiety should be conducted.

Acknowledgements: The authors thank the wider MAP ALLIANCE team from City University of London and the University of Stirling for input into the development of this review. We would also like to thank Yasmine Noorani, Academic Support Librarian at Bangor University, for her assistance in creating our search strategy. Additional thanks to Dr Catherine Lawrence for early input and feedback on this paper.

Contributors: The review was conceived by RTE, and the protocol was developed by LT, KP and LHS; searches were undertaken by KP; article screening was carried out by KP and LHS with mediation by LT; quality appraisal was undertaken by KP, LHS and LT; data were interpreted by all authors; the manuscript was drafted by KP and LHS and critically reviewed by all authors, RC, SA and RTE.

Funding: This review is to complement the MAP ALLIANCE study, funded by the National Institute for Health and Care Research (NIHR) (Award ID: NIHR133727). This rapid review was partially funded by Health and Care Economics Cymru (HCEC), an organisation funded by Health and Care Research Wales.

Conflict of interest: All authors declare that they have no conflicts of interest.

Ethical approval: The MAP ALLIANCE study received ethical approval from the West of Scotland Research Ethics Committee on 6th May 2022 (REC 3. Reference: 22/WS/0029).

Data statement: No datasets were generated and/or analysed for this study

For peer review only - http://bmjopen.bmj.com/site/about/guidelines.xhtml

References

[1] NHS England and NHS Improvement. The Perinatal Mental Health Care Pathways NHS England and NHS Improvement. 2018.

[2] Bauer A, Personage M, Knapp M, Lemmi V, Adeleja B. Costs of perinatal mental health problems. London: 2014.

[3] Petrou S, Cooper P, Murray L, Davidson LL, Lawson C, Woolgar M, et al. Cost-effectiveness of a preventive counseling and support package for postnatal depression. Int J Technol Assess Health Care 2006;22:443–53. https://doi.org/10.1017/S0266462306051361.

[4] Moore Simas TA, Huang MY, Packnett ER, Zimmerman NM, Moynihan M, Eldar-Lissai A. Matched cohort study of healthcare resource utilization and costs in young children of mothers with postpartum depression in the United States. J Med Econ 2020;23:174–83. https://doi.org/10.1080/13696998.2019.1679157.

[5] Giallo R, Woolhouse H, Gartland D, Hiscock H, Brown S. The emotional–behavioural functioning of children exposed to maternal depressive symptoms across pregnancy and early childhood: a prospective Australian pregnancy cohort study. Eur Child Adolesc Psychiatry 2015;24:1233–44. https://doi.org/10.1007/s00787-014-0672-2.

[6] Bauer A, Pawlby S, Plant DT, King D, Pariante CM, Knapp M. Perinatal depression and child development: Exploring the economic consequences from a South London cohort. Psychol Med 2015;45:51–61. https://doi.org/10.1017/S0033291714001044.

[7] Hardcastle K, Bellis MA, Ford K, Hughes K, Garner J, Ramos Rodriguez G. Measuring the relationships between adverse childhood experiences and educational and employment success in England and Wales: findings from a retrospective study. Public Health 2018;165:106–16. https://doi.org/10.1016/J.PUHE.2018.09.014.

[8] Posmontier B, Neugebauer R, Stuart S, Chittams J, Shaughnessy R. Telephone-Administered Interpersonal Psychotherapy by Nurse-Midwives for Postpartum Depression. J Midwifery Womens Health 2016;61:456–66. https://doi.org/10.1111/jmwh.12411.

[9] The National Institute of Health and Care Excellence. NICE | The National Institute for Health and Care Excellence 2022. https://www.nice.org.uk/ (accessed February 9, 2022).

[10] National Institute for Health and Care Excellence. Maternity and mental health 2022. https://www.nice.org.uk/about/what-we-do/into-practice/measuring-the-use-of-nice-guidance/impact-of-our-guidance/niceimpact-maternity/ch2-maternity-and-mental-health (accessed July 27, 2022).

For peer review only

For peer review only - http://bmjopen.bmj.com/site/about/guidelines.xhtml

[11] Maternal Mental Health Alliance. Map of Specialist Community Perinatal Mental Health Teams (UK). 2020.

[12] Page M, McKenzie J, Bossuyt P, Boutron I, Hoffmann T, Mulrow C. The PRISMA 2020 statement: an updated guideline for reporting systematic reviews. BMJ 2021;10. https://doi.org/doi.org/10.1186/s13643-021-01626-4.

[13] Health and Care Research Wales. Impact of the COVID-19 pandemic on the health and access to health care of disabled people: a rapid review 2022. https://healthandcareresearchwales.org/impact-covid-19-pandemic-disabled-people (accessed May 10, 2022).

[14] Health and Care Research Wales. Modelling studies used to evaluate the effect of population-level non-pharmaceutical interventions on the reproduction number of Severe Acute Respiratory Syndrome Coronavirus 2 (SARS-CoV-2) 2022. https://healthandcareresearchwales.org/modelling-studies-used-evaluate-effect-population-level-non-pharmaceutical-interventions (accessed May 10, 2022).

[15] Tricco AC, Khalil H, Holly C, Feyissa G, Godfrey C, Evans C, et al. Rapid reviews and the methodological rigor of evidence synthesis. JBI Evid Synth 2022;Publish Ah:944–9. https://doi.org/10.11124/jbies-21-00371.

[16] Mandrik O, Severens J, Bardach A, Ghabri S, Hamel C, Mathes T, et al. Critical Appraisal of Systematic Reviews With Costs and Cost-Effectiveness Outcomes: An ISPOR Good Practices Task Force Report 2021. https://doi.org/10.1016/j.

[17] Schardt C, Adams MB, Owens T, Keitz S, Fontelo P. Utilization of the PICO framework to improve searching PubMed for clinical questions. BMC Med Inform Decis Mak 2007;7:1–6.

[18] Drummond MF, Sculpher MJ, Claxton K, Stoddart GL, Torrance G W. Methods for the economic evaluation of health care programmes. 4th ed. Oxford: Oxford University Press; 2015.

[19] Moons KGM, de Groot JAH, Bouwmeester W, Vergouwe Y, Mallett S, Altman DG, et al. Critical Appraisal and Data Extraction for Systematic Reviews of Prediction Modelling Studies: The CHARMS Checklist. PLoS Med 2014;11. https://doi.org/10.1371/journal.pmed.1001744.

[20] Aromataris E, Fernandez R, Godfrey C, Holly C, Kahlil H, Tungpunkom P. Summarizing systematic reviews: methodological development, conduct and reporting of an Umbrella review approach. Int J Evid Based Healthc 2015 2015;13:132–40.

[21] Tufanaru C, Munn Z, Aromataris E, Campbell J, Hopp L. Systematic reviews of effectiveness. In: Aromataris E, Munn Z, editors. JBI Manual for Evidence Synthesis, JBI; 2020.

For peer review only - http://bmjopen.bmj.com/site/about/guidelines.xhtml

[22] Moola S, Munn Z, Tufanaru C, Aromataris E, Sears K, Sfetcu R, et al. Systematic reviews of etiology and risk. In: Aromataris E, Munn Z, editors. Manual for Evidence Synthesis, JBI; 2020.

[23] Morrell CJ, Spiby H, Stewart P, Walters S, Morgan A. Costs and effectiveness of community postnatal support workers: Randomised controlled trial. Br Med J 2000;321:593–8. https://doi.org/10.1136/BMJ.321.7261.593.

[24] Henderson C, Dixon S, Bauer A, Knapp M, Jane Morrell C, Slade P, et al. Cost-effectiveness of PoNDER health visitor training for mothers at lower risk of depression: Findings on prevention of postnatal depression from a cluster-randomised controlled trial. Psychol Med 2019;49:1324–34. https://doi.org/10.1017/S0033291718001940.

[25] Ride J, Lorgelly P, Tran T, Wynter K, Rowe H, Fisher J. Preventing postnatal maternal mental health problems using a psychoeducational intervention: the cost-effectiveness of What Were We Thinking. BMJ Open 2016;6. https://doi.org/10.1136/bmjopen-2016.

[26] Stevenson MD, Scope A, Sutcliffe PA. The Cost-Effectiveness of Group Cognitive Behavioral Therapy Compared with Routine Primary Care for Women with Postnatal Depression in the UK. Value in Health 2010;13:580–4. https://doi.org/10.1111/j.1524-4733.2010.00720.x.

[27] Grote NK, Simon GE, Russo J, Lohr MJ, Carson K, Katon W. Incremental benefit-cost of MOMCare: Collaborative care for perinatal depression among economically disadvantaged women. Psychiatric Services 2017;68:1164–71. https://doi.org/10.1176/APPI.PS.201600411.

[28] Petrou S, Cooper P, Murray L, Davidson LL. Economic costs of post-natal depression in a high-risk British cohort. British Journal of Psychiatry 2002;181:505–12. https://doi.org/10.1192/bjp.181.6.505.

[29] Dagher RK, McGovern PM, Dowd BE, Gjerdingen DK. Postpartum depression and health services expenditures among employed women. J Occup Environ Med 2012;54:210–5. https://doi.org/10.1097/JOM.0B013E31823FDF85.

[30] Ammerman RT, Chen J, Mallow PJ, Rizzo JA, Folger AT, van Ginkel JB. Annual direct health care expenditures and employee absenteeism costs in high-risk, low-income mothers with major depression. J Affect Disord 2016;190:386–94. https://doi.org/10.1016/J.JAD.2015.10.025.

[31] Roberts J, Sword W, Watt S, Gafni A, Krueger P, Sheehan D, et al. Costs of postpartum care examining associations from the Ontario mother and infant survey. Canadian Journal of Nursing Research 2001;33:19–34.

[32] Counts NZ, Kuklinski MR, Wong V, Feinberg ME, Creedon TB. Comparison of Estimated Incentives for Preventing Postpartum Depression in Value-Based Payment Models Using the Net Present Value of Care vs Total Cost of Care. JAMA Netw Open 2022;5:e229401. https://doi.org/10.1001/JAMANETWORKOPEN.2022.9401.

For peer review only - http://bmjopen.bmj.com/site/about/guidelines.xhtml

[33] Franta G, Hersh AR, Cirino NH, Caughey AB. Prevention of perinatal depression with counseling in adolescents: a cost-effectiveness analysis. The Journal of Maternal-Fetal & Neonatal Medicine: The Official Journal of the European Association of Perinatal Medicine 2022. https://doi.org/10.1080/14767058.2022.2049746.

[34] Ride J. Setting the Boundaries for Economic Evaluation: Investigating Time Horizon and Family Effects in the Case of Postnatal Depression. Value in Health 2018;21:573–80. https://doi.org/10.1016/j.jval.2017.10.016.

[35] Wilkinson A, Anderson S, Wheeler SB. Screening for and Treating Postpartum Depression and Psychosis: A Cost-Effectiveness Analysis. Matern Child Health J 2017;21:903–14. https://doi.org/10.1007/s10995-016-2192-9.

[36] Chojenta C, William J, Martin MA, Byles J, Loxton D. The impact of a history of poor mental health on health care costs in the perinatal period. Arch Womens Ment Health 2019;22:467–73. https://doi.org/10.1007/S00737-018-0912-4.

[37] Bank of England. Inflation calculator 2022. https://www.bankofengland.co.uk/monetary-policy/inflation/inflation-calculator (accessed August 18, 2022).

[38] U.S. Bureau of Labor Statistics. CPI Inflation Calculator 2022. https://www.bls.gov/data/inflation_calculator.htm (accessed August 18, 2022).

[39] Google. Google currency converter 2022. https://www.google.com/search?q=currency+convertor&rlz=1C1GCEA_enGB991GB991&oq=currency+convertor&aqs=chrome..69i57j0i10i131i433l2j0i10l7.4286j1j3&sourceid=chrome&ie=UTF-8 (accessed September 5, 2022).

[40] Bank of Canada. Inflation Calculator 2022. https://www.bankofcanada.ca/rates/related/inflation-calculator/ (accessed August 18, 2022).

[41] Reserve Bank of Australia 2022. https://www.rba.gov.au/calculator/annualDecimal.html (accessed March 29, 2023).

[42] Camacho EM, Shields GE. Cost-effectiveness of interventions for perinatal anxiety and/or depression: A systematic review. BMJ Open 2018;8. https://doi.org/10.1136/bmjopen-2018-022022.

[43] Gurung B, Jackson LJ, Monahan M, Butterworth R, Roberts TE. Identifying and assessing the benefits of interventions for postnatal depression: A systematic review of economic evaluations. BMC Pregnancy Childbirth 2018;18. https://doi.org/10.1186/S12884-018-1738-9.

[44] Henderson J. The cost effectiveness of prenatal care - ProQuest 1994. https://www.proquest.com/assia/docview/196971225/abstract/36297FA264EA438FPQ/1?accountid=14874 (accessed May 21, 2022).

For peer review only

[45] Morrell CJ, Sutcliffe P, Booth A, Stevens J, Scope A, Stevenson M, et al. A systematic review, evidence synthesis and meta-analysis of quantitative and qualitative studies evaluating the clinical effectiveness, the cost-effectiveness, safety and acceptability of interventions to prevent postnatal depression. Health Technol Assess 2016;20:1–414. https://doi.org/10.3310/HTA20370.

[46] Cox JL, Chapman G, Murray D, Jones P. Validation of the Edinburgh postnatal depression scale (EPDS) in non-postnatal women. vol. 39. 1996.

[47] Moran PS, Wuytack F, Turner M, Normand C, Brown S, Begley C, et al. Economic burden of maternal morbidity – A systematic review of cost-of-illness studies. PLoS One 2020;15. https://doi.org/10.1371/JOURNAL.PONE.0227377.

[48] Ride J. Setting the Boundaries for Economic Evaluation: Investigating Time Horizon and Family Effects in the Case of Postnatal Depression 2018. https://doi.org/10.1016/j.jval.2017.10.016.

For peer review only

For peer review only - http://bmjopen.bmj.com/site/about/guidelines.xhtml

Figure legend

Figure 1. PRISMA study selection flowchart (Page et al., 2021b)

For peer review only

For peer review only - http://bmjopen.bmj.com/site/about/guidelines.xhtml

Figure 1. PRISMA study selection flowchart (Page et al., 2021b)

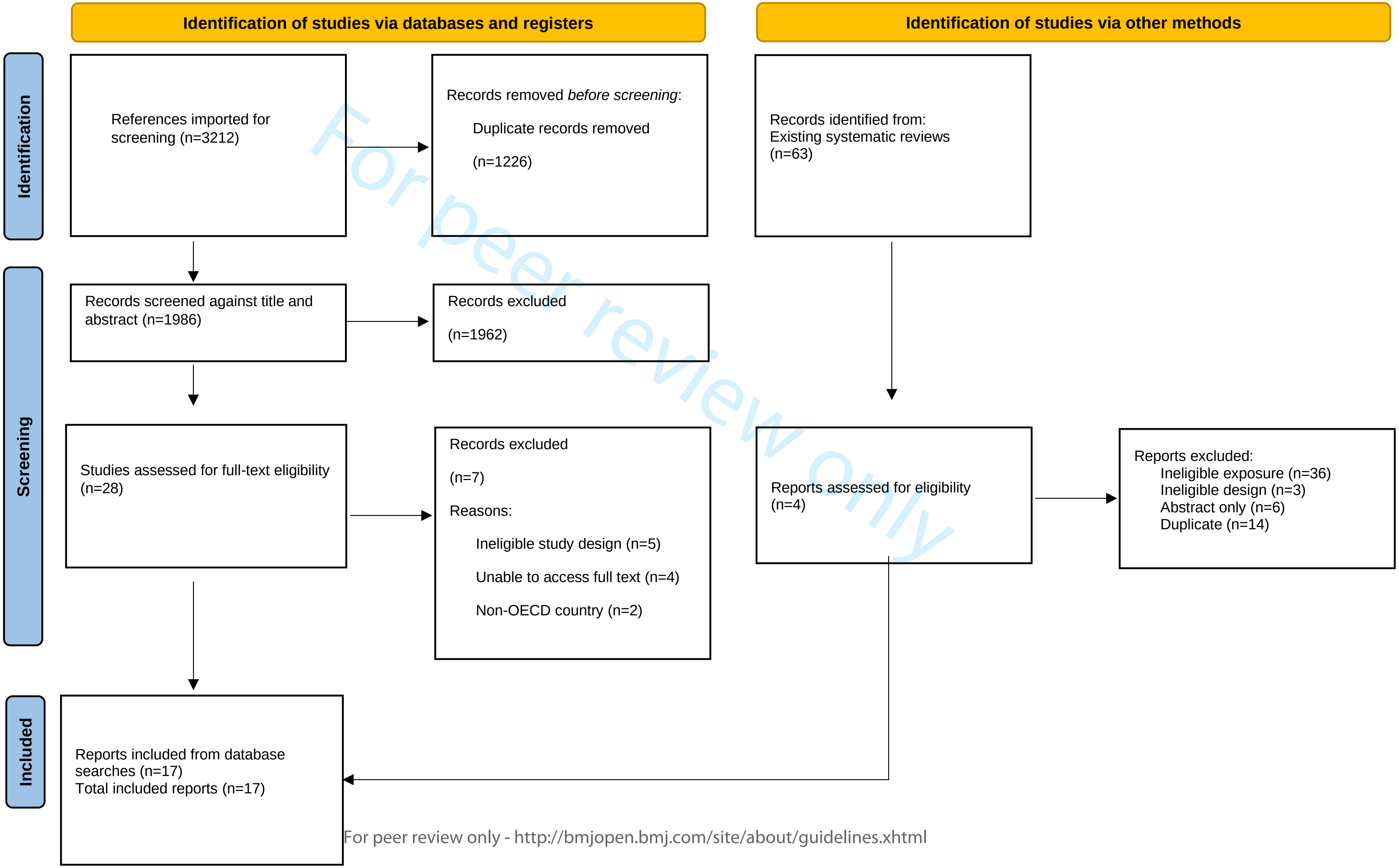

For peer review only

For peer review only - http://bmjopen.bmj.com/site/about/guidelines.xhtml

Search strategy

Below is an example of a search strategy for the Medline database.

ID	Search terms
1	exp Pregnancy/
2	(pregnan* or childbearing).ti,ab,kw.
3	(postpartum or post-partum or postnatal or postnatal or perinatal or peri-natal or prenatal or pre-natal or antenatal or ante-natal or matern*).ti,ab,kw.
4	perinatal anxiety.ti,ab,kw. or exp Perinatal anxiety/
5	exp Depression/
6	depress$.ti,ab,kw.
7	5 or 6
8	(Infant or baby or child).ti,ab,kw
9	(care* or treatment).tiab.kw
10	NHS.ti,ab,kw
11	hospitali$ation*.ti,ab,kw
12	exp Resource allocation/
13	economic evaluation$.ti,ab,kw.
14	(cost* or economic* or pharmacoeconomic*).ti.
15	13 or 14
16	exp "costs and cost analysis"/ or exp Health Care Costs/
17	exp Cost-Benefit Analysis/
18	(cost* adj2 (effective* or utility* or benefit* or consequence* or minimi*)).ti,ab,kw.
19	16 or 17 or 18
20	quality-adjusted life year$.ti,ab,kw. or exp Quality-Adjusted Life Years/
21	Or 7 and 15 and 19

For peer review only

For peer review only - http://bmjopen.bmj.com/site/about/guidelines.xhtml

Quality appraisal of health economic evaluation studies [24]

**Drummond et al checklist 2015**	**Petrou et al (2002)** [40]	**Petrou et al (2006)** [4]	**Ride et al (2016)** [32]	**Henderson et al (2019)** [38]
1. Was a well defined question posed in an answerable form?	Yes	Yes	Yes	Yes
2. Was a comprehensive description of the competing alternatives given?	n/a	n/a	Yes	Yes
3. Was the effectiveness of the programs or services established?	n/a	n/a	Yes	Yes
4. Were all the important and relevant costs and consequences for each alternative identified?	n/a	n/a	Yes	Yes
5. Were costs and consequences measured accurately in appropriate physical units?	Yes	Yes	Yes	Yes
6. Were costs and consequences valued credibly?	Yes	Yes	Yes	Yes
7. Were costs and consequences adjusted for differential timing	n/a	n/a	No	No
8. Was an incremental analysis of costs and consequences of alternatives performed?	n/a	n/a	No	Yes
9. Was allowance made for uncertainty in the estimates of costs and consequences?	Yes	Yes	Yes	Yes
10. Did the presentation and discussion of study results include all issues of concern to users?	Yes	Yes	Yes	Yes

Source of checklist: Drummond, M. F., Sculpher, M. J., Claxton, K., Stoddart, G. L., & Torrance G W. (2015). *Methods for the economic evaluation of health care programmes* (4th ed.). Oxford: Oxford University Press.

For peer review only - http://bmjopen.bmj.com/site/about/guidelines.xhtml

Quality appraisal of health economic modelling studies with CHARMS Checklist [25]

Domain	Key items	Counts et al (2022) - [34]	Franta et al (2022) - (Franta et al., 2022)	Ride (2018) - [45]	Wilkinson et al (2017) - [5]	Bauer et al (2015) (Bauer et al, 2015)	Stevenson et al, (2010) [42]
SOURCE OF DATA	Source of data (e.g., cohort, case-control, randomized trial participants.	p.3	p.2	p.575	p.3	p.52	p.581
PARTICIPANTS	Participant eligibility and recruitment method (e.g., consecutive participants, location,	p.3	p.2	p.575	p.3	p.52	p.581
	Participant description	p.3	p.2	p.575	p.3	p.52	p.581
	Details of treatments received, if	p.5	p.2	p.575	p.3	p.52	N/A
	Study dates	p.4	p.2	p.575	p.3	p.52	p.581
OUTCOME(S) TO BE PREDICTED	Definition and method for measurement of outcome	p.4	p.2	p.574	p.4	p.53	p.581-582
	Was the same outcome definition (and method for measurement) used in all	Yes p.5	p.2	p.574	p.4	p.53	p.581-582
	Type of outcome (e.g., single or combined	p.3	p.5	p.574	p.4	p.53	p.581
	Was the outcome assessed without knowledge of	No	No	No	No	No	p.581
	Were candidate predictors part of the outcome	No	No	No	No	No	p.581

For peer review only

For peer review only - http://bmjopen.bmj.com/site/about/guidelines.xhtml

	Time of outcome occurrence or summary of duration of follow-up	p.5	p.5	p.578	p.4	p.52	p.581
**CANDIDATE PREDICTORS (OR INDEX TESTS)**	Number and type of predictors (e.g., demographics, patient history, physical examination,	p.5	p.5	p.577	p.6	p.55	p.582
	Definition and method for measurement of candidate predictors	p.5	p.5	p.575	p.6	p.55	p.580-582
	Timing of predictor measurement (e.g., at patient presentation, at diagnosis,	p.5	p.5	p.577	p.6	p.55	p.581
	Were predictors assessed blinded for outcome, and for each other	No	No	No	No	No	p.582
	Handling of predictors in the modelling (e.g., continuous, linear, non-linear transformatio	Unclear	Unclear	Unclea r	Unclear	p.52	p.582
**SAMPLE SIZE**	Number of participants and number of outcomes/ev	p.3	p.2	p.575	P.3	p.55	p.582
	Number of outcomes/ev ents in relation to the number of candidate predictors (Events Per Variable)	p.5	p.3	p.577	p.20	p.57	p.582
**MISSING DATA**	Number of participants with any missing value	p.4	Unclear	Unclea r	Unclear	Unclea r	Unclear

For peer review only - http://bmjopen.bmj.com/site/about/guidelines.xhtml

	Number of participants with missing data for each predictor	Unclear	Unclear	Unclea r	Unclear	Unclea r	Unclear
	Handling of missing data (e.g., complete-case analysis, imputation, or	Unclear	Unclear	Unclea r	Unclear	Unclea r	Unclear
**MODEL DEVELOPMEN T**	Modelling method (e.g., logistic, survival, neural network, or	Simulate d cohort model	Simulat ed cohort model	Decisi on analyti c model	Simulat ed cohort model	Decisi on analyti c model	Mathemati cal model
	Modelling assumptions satisfied	See Appendix 1 in the supplem ent	p.5	p.577	p.4	p.53	p.580
	Method for selection of predictors **for inclusion** in multivariable modelling (e.g., all candidate predictors, pre-selection based on unadjusted association with the	Unclear	Unclear	p.577	p.4	p.53	p.581
	Method for selection of predictors **during multivariable modelling** (e.g., full model approach, backward or forward selection) and criteria used (e.g., p-value, Akaike Information Criterion)	Unclear	Unclear	Unclea r	Unclear	p.53	Unclear

For peer review only - http://bmjopen.bmj.com/site/about/guidelines.xhtml

	Shrinkage of predictor weights or regression coefficients (e.g., no shrinkage, [illegible]	Unclear	Unclear	Unclea r	Unclear	Unclea r	Unclear
**MODEL PERFORMANCE**	Calibration (calibration plot, calibration slope, Hosmer-Lemeshow test) and Discrimination (C-statistic, D-statistic, log-rank)	p.5	Unclear	Unclea r	Unclear	Unclea r	Unclear
	Classification measures (e.g., sensitivity, specificity, predictive values, net reclassification improvement) and whether a-priori cut [illegible]	See e-appendix 3	p.6	p.577	p.6	No	p.581
**MODEL EVALUATION**	Method used for testing model performance: development dataset only (random split of data, resampling methods e.g. bootstrap or cross-validation, none) or separate external validation (e.g. temporal, geographical	See e-appendix 3	Unclear	Unclea r	p.6	No	Unclear

For peer review only

	In case of poor validation, whether model was adjusted or updated (e.g., intercept recalibrated, predictor effects	Unclear	Unclear	Unclea r	Unclear	No	Unclear
RESULTS	Final and other multivariable models (e.g., basic, extended, simplified) presented, including predictor weights or regression coefficients, intercept, baseline survival, model performance measures (with	Unclear	Unclear	Unclea r	Unclear	No	No
	Any alternative presentation of the final prediction models, e.g., sum score, nomogram, score chart, predictions for specific risk subgroups	No	No	p.578	p.23	No	No
	Comparison of the distribution of predictors (including missing data) for development and validation	No	No	No	No	No	No

For peer review only - http://bmjopen.bmj.com/site/about/guidelines.xhtml

For peer review only

**INTERPRETATION AND DISCUSSION**	Interpretation of presented models (confirmatory, i.e., model useful for practice versus exploratory, i.e., more research	p.7	p.5	p.577	p.6	p.56	p.583
	Comparison with other studies, discussion of generalizability, strengths and limitations.	p.7	p.5	p.577	p.6	p.58	p.583

JBI critical appraisal checklist for Systematic Reviews and Research Syntheses [26]

Citation	Q1. Is the review question clearly and explicitly stated?	Q2. Were the inclusion criteria appropriate for the review question?	Q3. Was the search strategy appropriate?	Q4. Were the sources and resources used to search for studies adequate?	Q5. Were the criteria for appraising studies appropriate?	Q6. Was critical appraisal conducted by two or more reviewers independently?	Q7. Were there methods to minimize errors in data extraction?	Q8. Were the methods used to combine studies appropriate?	Q9. Was the likelihood of publication bias assessed?	Q10. Were recommendations for policy and/or practice supported by the reported data?	Q11. Were the specific directives for new research appropriate?
(Camacho & Shields, 2018)	Yes	Yes	Yes	Yes	Yes	Unclear	Yes	Yes	Yes	Yes	Yes
[37]	Yes	Yes	Yes	Yes	Yes	Unclear	Yes	Unclear	Yes	Yes	Yes
(Moran et al., 2020)	Yes	Yes	Yes	Yes	Yes	Yes	Yes	Yes	Yes	Unclear	Yes
(Morrell et al., 2016)	Yes	Yes	Yes	Yes	Yes	Yes	Yes	Yes	Yes	Yes	Yes

JBI Critical appraisal checklist for randomized controlled trials [27]

Citation	Q1. Was true randomization used for assignment of participants to treatment groups?	Q2. Was allocation to treatment groups concealed?	Q3. Were treatment groups similar at the baseline?	Q4. Were participants blind to treatment assignment?	Q5. Were those delivering treatment blind to treatment	Q6. Were outcomes assessors blind to treatment	Q7. Were treatment groups treated identically other than the	Q8. Was follow up complete and if not, were differences	Q9. Were participants analyzed in the groups to which they were	Q10. Were outcomes measured in the same way for	Q11. Were outcomes measured in a reliable way?	Q12. Was appropriate statistical analysis used?	Q13. Was the trial design appropriate, and any deviations from the standard

8

For peer review only - http://bmjopen.bmj.com/site/about/guidelines.xhtml

					assignment?	assignment?	intervention of interest?	between groups in terms of their follow up adequately described and analyzed?	randomized?	treatment groups?			RCT design (individual randomization, parallel groups) accounted for in the conduct and analysis of the trial?
[29]	Yes	Yes	Yes	Unclear	Yes	Unclear	Yes	Yes	No	Yes	Unclear	Yes	N/A
[44]	Yes	Yes	Yes	Yes	Yes	Unclear	Yes	Yes	Yes	Yes	Yes	Yes	Yes

JBI Critical Appraisal Checklist for Cohort Studies [28]

**Citation**	Q1. Were the two groups similar and recruited from the same population?	Q2. Were the exposures measured similarly to assign people to both exposed and unexposed groups?	Q3. Was the exposure measured in a valid and reliable way?	Q4. Were confounding factors identified?	Q5. Were strategies to deal with confounding factors stated?	Q6. Were the groups/ participants free of the outcome at the start of the study (or at the moment of exposure)?	Q7. Were the outcomes measured in a valid and reliable way?	Q8. Was the follow up time reported and sufficient to be long enough for outcomes to occur?	Q9. Was follow up complete, and if not, were the reasons to loss to follow up described and explored?	Q10. Were strategies to address incomplete follow up utilized?	Q11. Was appropriate statistical analysis used?
(Moore Simas et al., 2020)	Yes	Yes	Yes	No	No	Yes	Yes	Yes	Unclear	N/A	Yes

JBI Critical Appraisal Checklist for Cross-sectional studies [28]

Citation	Q1. Were the criteria for inclusion in the sample clearly defined?	Q2. Were the study subjects and the setting described in detail?	Q3. Was the exposure measured in a valid and reliable way?	Q4. Were objective, standard criteria used for measurement of the condition?	Q5. Were confounding factors identified?	Q6. Were strategies to deal with confounding factors stated?	Q7. Were the outcomes measured in a valid and reliable way?	Q8. Was appropriate statistical analysis used?
Dagher et al., 2012	Yes	Yes	Yes	Yes	No	No	Yes	Yes
Chojenta et al., 2019	Yes	Yes	Yes	Yes	Yes	Yes	Yes	Unclear
Ammerman et al., 2016	Yes	Yes	Yes	Yes	Yes	Yes	Yes	Yes
Roberts et al., 2001	Yes	Yes	Yes	Yes	No	N/A	Yes	Yes

For peer review only - http://bmjopen.bmj.com/site/about/guidelines.xhtml

Abbreviations

Abbreviation	Full	Aspect
ANOVA	Analysis of Variance	Analysis
ANRQ-R	Antenatal Risk Questionnaire	Tool
CATi	Computer Assisted Telephone Interviews	Research
CBA	Cognitive Behavioural Approach	Intervention
CBT	Cognitive Behavioural Therapy	Intervention
CEA	Cost Effectiveness Analysis	Analysis
CIDI	Composite International Diagnostic Interview	Research
CUA	Cost Utility Analysis	Analysis
DASS21	Depression, Anxiety and Stress Scale	Tool
DCS	Depression Care Specialist	Staff
DFD	Disease Free Day	Research
DSM-IV	Diagnostic and Statistical Manual for Mental Disorders 4th Edition	Source
eMBI	electronic Mindfulness-based Intervention	Intervention
EPDS	Edinburgh Postnatal Depression Scale	Tool
ePRO	electronic Patient Reported Outcomes	Research
EQ-5D-3L	EuroQol 5 Dimension 3 Level	Tool
GP	General Practitioner	Staff
gCBT	Group cognitive behavioural therapy	Intervention
HRU	Healthcare resource utilization	Analysis
HV	Health Visitor	Staff
ICD	International Classification of Diseases	Source
ICER	Incremental Cost-Effectiveness Ratio	Analysis
IG	Intervention Group	Research
IPT	Interpersonal psychotherapy	Intervention
ITT	Intention to Treat	Research
LGA	Local Government Area	Organisation
MBS	Medical Benefits Schedule	Source
MCH	Maternal and Child Health	Setting
MFAS	Maternal-Fetal Attachment Scale	Tool
MOMcare		*Study name*
MINI	Mini-International Neuropsychiatric Interview	Tool
NHS	National Health Service	Setting
OOP	Out of Pocket	Research
PAD	perinatal anxiety and/or depression	Diagnosis
PBS	Pharmaceutical Benefits Scheme	Source
PCA	Personalised Care Approach	Intervention
PHQ-9	Patient Health Questionnaire	Tool
PND	Postnatal depression	Diagnosis
PND	Post-partum depression	Diagnosis
PoNDER trial	POstNatal Depression Economic evaluation and Randomised trial	*Study name*
PRAQ-R	Pregnancy-Related Anxiety Questionnaire	Tool
PTSD	Post-Traumatic Stress Disorder	Diagnosis
QALY	Quality Adjusted Life Year	Analysis

For peer review only - http://bmjopen.bmj.com/site/about/guidelines.xhtml

RCT	Randomised controlled trial	Research
SCL-20	Hopkins Symptom Checklist-20	Tool
SF36	Short-Form 36	Tool
SIDS	Sudden infant death syndrome	Diagnosis
SPARCS	*Sleep, Parenting and Relationships in a Community Setting*	*Study name*
STAI	State-Trait Anxiety Questionnaire	Tool
TAU	Treatment as Usual	Research
TENS	Transcutaneous Electrical Nerve Stimulation	Intervention
WHO	World Health Organisation	Organisation
WWWT	What Were We Thinking	Tool

For peer review only

For peer review only - http://bmjopen.bmj.com/site/about/guidelines.xhtml

Data extraction table for studies including perinatal anxiety

Citation (Country)	Study Details	Participants and setting	Key findings	Observations
Ride et al (2016)<br><br>(Ride et al., 2016)<br><br>**Australia**	**Study Design:** Economic evaluation, including cost-effectiveness and cost-utility analyses, conducted alongside a cluster-randomised trial<br><br>**Type of intervention [*exposure*]:** What Were We Thinking (WWWT) - a psychoeducational intervention targeted at the partner relationship, management of infant behaviour and parental fatigue.<br><br>**Data collection methods:** Data were collected from participants via computer-assisted telephone interview at baseline (6 weeks postpartum) and follow-up (26 weeks postpartum).	**Sample size:** 359<br><br>**Participants:** English-speaking first-time mothers who had recently given birth and attended participating Maternal and Child Health Centres (MCHCs)<br><br>**Setting:** 48 Maternal and Child Health Centres in Victoria, Australia.<br><br>**Dates of data collection:** Baseline interviews took place between May 2013 and April 2014, and follow-up interviews between September 2013 and August 2014.	**Primary Findings:**<br>The intervention was estimated to cost $A118.16 per participant. The analysis showed no statistically significant difference between the intervention and control groups in costs or outcomes. The incremental cost-effectiveness ratios were $A36 451 per QALY gained and $A152 per percentage point reduction in 30-day prevalence of depression, anxiety, and adjustment disorders. The estimate lies under the unofficial cost-effectiveness threshold of $A55 000 per QALY; however, there was considerable uncertainty surrounding the results, with a 55% probability that WWWT would be considered cost-effective at that threshold.<br>**Additional Findings:**<br>The results suggest that, although WWWT shows promise as a preventive intervention for postnatal maternal mental health problems, further research is required to reduce the uncertainty over its cost-effectiveness as there were no statistically significant differences in costs or outcomes.	Ride et al (2016) investigated the cost-effectiveness of the What Were We Thinking (WWWT) intervention, for the prevention of postnatal maternal mental health problems. The intervention was estimated to cost $A118.16 per participant. The analysis showed no statistically significant difference between the intervention and control groups in costs or outcomes. The incremental cost-effectiveness ratios were $A36 451 per QALY gained and $A152 per percentage point reduction in 30-day prevalence of depression, anxiety, and adjustment disorders. The estimate lies under the unofficial cost-effectiveness threshold of $A55 000 per QALY; however, there was considerable uncertainty surrounding the results, with a 55% probability that WWWT would be considered cost-effective at that threshold

For peer review only - http://bmjopen.bmj.com/site/about/guidelines.xhtml

Data extraction table for studies including maternal depression

Citation (Country)	Study Details	Participants and setting	Key findings	Observations
Ammerman et al (2016)<br><br>(Ammerman et al., 2016)<br><br>USA	**Study design:** Cross-sectional<br><br>**Data collection methods:** MEPS database, a subset of the National Health Interview Survey (NHIS) that includes information on health care utilisation and expenditures for the civilian, non-institutionalised population in the USA.	**Sample size:** 20,531<br><br>**Participants:** 2,310 high-risk mothers with depression and 18,221 high-risk mothers without depression<br><br>**Setting:** USA healthcare setting<br><br>**Dates of data collection:** 1996 to 2011	**Primary findings:**<br>Depressed mothers were more likely to incur insurer (0.88 vs. 0.80) and out of pocket expenses (0.86 vs. 0.77) and to have higher insurer expenses ($4916 vs. $3521) and out of pocket expenses ($786 vs. $522) (in 2015).<br>**Additional findings:**<br>A higher proportion of the depressed sample was Caucasian which were in relatively worse health than women from other ethnic groups. The depressed sample was more likely to have public insurance, to be English-speaking and to have a usual health care provider.	The cross-sectional study from Ammerman et al (2016) the USA conducted between 2006 and 2011 investigated the out-of-pocket expenses and insurer expenses of depressed vs non-depressed mothers. Depressed mothers were more likely to incur insurer and out of pocket expenses and to have higher insurer expenses ($4916 vs. $3521) and out of pocket expenses ($786 vs. $522) (in 2015).
Bauer et al (2015)<br><br>(Bauer et al., 2015)<br><br>UK	**Study Design:** The economic analysis takes a life-course perspective from the viewpoints of the public sector, individual and society. The study analysed the effects of perinatal depression on child development outcomes of children at ages 11 and 16 years from the community-based South London Child Development Study. Economic consequences were attached to those outcomes through simple decision-analytic techniques, building on evidence from studies of epidemiology, health-related quality of life, public sector costs and employment.	**Sample size:** 120<br><br>**Participants:** Mothers and children<br><br>**Setting:** Two antenatal clinics in the UK<br><br>**Dates of data collection:** January to December 1986	**Primary Findings:**<br>Additional risks that children exposed to perinatal depression develop emotional, behavioural, or cognitive problems ranged from 5% to 21%. In addition, there was a high risk (24%) that children would have special educational needs.<br><br>For each child exposed to perinatal depression, public sector costs exceeded £3,030, costs due to reduced earnings were £1,400 and health-related quality of life loss was valued at £3,760.	The study examined some of the outcomes and long-term economic implications experienced by offspring who have been exposed to perinatal depression.
Counts et al (2022)<br><br>(Counts et al., 2022)<br><br>USA	**Study Design:** Modelling study. A decision analytic model used a simulated cohort of 1,000 Medicaid-enrolled pregnant individuals. Health care costs for individuals receiving postpartum depression preventive intervention or not, over 1 or 5 years postpartum, in a variety of scenarios, including varying rates of Medicaid churn (i.e., transitions to a new Medicaid managed care plan, commercial insurance plan, or loss of coverage) were	**Sample size:** 1,000<br><br>**Participants:** simulated cohort of 1,000 Medicaid enrolled pregnant individuals<br><br>**Setting:** USA healthcare system.	**Primary Findings:**<br>The main outcome was the amount of clinician incentive shared in a Value-based payment (VBP) model from providing preventive interventions. The likelihood of the health care payer realising a positive return on investment if it shared 50% of 5-year	This economic modelling study found that providing preventive interventions for PND resulted in an estimated 5-year saving of £602▫

	estimated for the period 2020 to 2025. The model was developed between March 5 2021 and July 30 2021.<br><br>**Type of intervention [*exposure*]:** Individual counselling and group-based counselling.<br><br>**Data collection methods:** Simulation based on collected Medicaid data.	**Dates of data collection:** Model developed between March 5 2021 and July 30 2021.	expected savings with a clinician up front was also measured.<br><br>The simulated cohort was designed to be reflective of the demographics characteristics of pregnant individuals receiving Medicaid; however, no specific demographic features were simulated. Providing preventive interventions for postpartum depression resulted in an estimated 5-year savings of $734.12 (95% credible interval [CrI], $217.21-$1235.67) per person. Without health insurance churn, sharing 50% of 5-year expected savings could offer more than double the financial incentives for clinicians to prevent postpartum depression compared with traditional VBP ($367.06 [95% CrI, $108.61-$617.83] vs $177.74 [95% CrI, $52.66-$296.60], respectively), with a high likelihood of positive return for the health care payer (91%). As health insurance churn increased, clinician incentives from sharing estimated savings decreased (73% reduction with 50% annual churn).	
Dagher et al (2012)<br><br>(Dagher et al., 2012<br><br>USA	**Study design:** Cross-sectional<br><br>**Data collection methods:** Prices of service use and EPDS	**Sample size:** 638 women.<br><br>**Participants:** Women receiving maternal healthcare services, from hospital discharge to 11 weeks postpartum.<br><br>**Setting:** USA healthcare setting.<br><br>**Dates of data collection:** The year 2001.	**Primary findings:**<br>The total cost of all mental health counselling visits for the depressed group n =31 was $138 and the cost for the non-depressed group n= 607 was $13. This was a statistically significant difference (p < 0.001).<br><br>**Additional findings:** The total cost of emergency department visits for the postpartum women was $84 for the depressed group n = 31 and $13 for the non-depressed group n = 607. This was a statistically significant difference (p < 0.001).	The Dagher et al., (2012) cross-sectional study from the USA investigated expenditure from health care service from discharge until 11 weeks postpartum. There was a significant difference in healthcare expenditure between depressed and non-depressed women. The EPDS was used to measure depression. The total cost of all mental health counselling visits for the depressed group n =31 was $138 and the cost for the non-depressed group n= 607 was $13. This was a statistically significant difference (p < 0.001).

For peer review only - http://bmjopen.bmj.com/site/about/guidelines.xhtml

Franta et al (2022)<br><br>(Franta et al., 2022)<br><br>USA	**Study Design:**<br>Modelling study<br><br>**Type of intervention [*exposure*]:**<br>Comparison of outcomes in pregnant adolescents who received versus did not receive counselling interventions<br><br>**Data collection methods**: Decision-analytic model using TreeAge Pro software	**Sample size:** Theoretical cohort of 180,000 individuals<br><br>**Participants:** pregnant adolescents<br><br>**Setting:** Obstetric setting<br><br>**Dates of data collection:**<br>2018	**Primary Findings:**<br>• A strategy of referral to counselling interventions was cost effective in the theoretical cohort, with 8,935 fewer cases of perinatal depression, 1,606 fewer cases of chronic depression, 166 fewer preterm deliveries, 4 fewer neonatal deaths, 1 fewer case of cerebral palsy, 20 fewer cases of SIDS. In total, there were 21,976 additional QALYs and cost savings of $223,549,872, making it the dominant strategy (better outcomes with lower costs).<br>• Counselling interventions remained cost saving until the annual direct and indirect cost of chronic, severe depression was set below $30,000, at which point it became cost effective (baseline input: $182,309).<br>• It is cost effective to refer all pregnant adolescents for preventive counselling interventions.	Using a theoretical cohort, Franta et al. (2022) found that counselling was a cost-effective preventative measure, leading to fewer cases of perinatal and chronic depression
Grote et al (2017)<br><br>Grote et al., 2017)<br><br>**USA**	**Study Design:** RCT, cost-benefit study<br><br>**Type of intervention [*exposure*]:** 18 months **MOMCare** collaborative care depression intervention (choice of brief interpersonal psychotherapy or pharmacotherapy or both) with enhanced maternity support services (MSS-Plus).<br><br>**Data collection methods:** Blinded telephone assessments, including depression severity on SCL-20. Unit costs of MOMCare intervention actual salary rate + fringe benefits + 30% overheads	**Sample size:** 152<br><br>**Participants:** 152 pregnant women 12-32 wks. gestation with probable major depression or dysthymia (PTSD). Plus 12 excluded from analysis due to missing final data.<br><br>**Setting:** 10 county public health centres<br><br>**Dates of data collection:** Recruited Jan 2010 – July 2012. Study ended 2014	**Primary Findings:** when controlled for baseline depression severity, women with probable depression and PTSD in MOMCare had 68 more depression-free days over 18 months than those in MSS-Plus (p,.05). Additional $1,312. depression care cost per MOMCare participant with comorbid PTSD. Incremental net benefit of MOMCare was positive if a depression free days was valued at ≥ $20<br><br>**Additional Findings:**<br>Unit costs used 2013:<br>$80 per 45-50 min depression care specialist (DCS) visit<br>$31 per 20-30 min DCS phone call<br>(Both included time for outreach efforts and record keeping)<br>$247 fixed cost per patient for caseload supervision and info support<br>Other references to US-based data sources	In this RCT, cost-benefit study, a multicomponent collaborative care intervention for depression (MOMcare - a choice of brief interpersonal psychotherapy or pharmacotherapy or both) with enhanced maternity support services (MSS-Plus) in the public health system of Seattle, USA. The incremental benefit and cost and the net benefit for women with major depression and PTSD was estimated. When controlled for baseline depression severity, women with probable depression and PTSD in MOMCare had 68 more depression-free days over 18 months than those in MSS-Plus (p<.05). There was an additional £1,943[illegible] depression care cost per MOMCare participant with comorbid PTSD. The incremental net benefit of MOMCare was positive if

For peer review only

				depression free days was valued below £18▪▪. For women with probable major depression and PTSD, MOMCare had a significant clinical benefit over MSS-Plus, with only a moderate increase in health services cost.[1]
Henderson et al (2019)<br><br>(Henderson et al., 2019)<br><br>**UK**	**Study Design:** PONDER Cluster RCT<br><br>**Type of intervention [*exposure*]:** GP practices assigned to usual health visitor (HV) care, HV trained to assess for PND plus offering either a CBA or a person-centred approach (PCA) weekly for 8 weeks<br><br>**Data collection methods:** Postal questionnaires: Baseline including EPDS and SF36 at 6 weeks, Postnatal questionnaires at 6, 12 and 18 months postnatal. Resource use logs were completed by HVs based on their and GP records	**Sample size: From** 101 GP practices, 4,084 participants consented, baseline data from 3,449 participants.<br><br>**Participants:** 2,241 lower risk women completed EPDS at 6 months – 767 control, 1,474 intervention. 1,459 women provided economic data.<br><br>**Setting:** GP practices<br><br>**Dates of data collection:** April 2003 for 3 years	**Primary Findings:** 99% probability of cost effectiveness at £20,000 at 6 months postnatal<br>Compared with controls, adjusted 6 months costs were £82 lower with the interventions<br><br>**Additional Findings:**<br>Little difference CBA to PCA – CBA marginally higher probability of being cost effective.	This study found that CBT had a marginally higher probability of being cost-effective than a person-centred approach.
Moore Simas et al (2020)<br><br>(Moore Simas et al., 2020)<br><br>USA	**Study Design:** Cohort study<br><br>**Type of intervention [*exposure*]:** PND.<br><br>**Data collection methods**: Administrative claims data from the IBM Watson Health MarketScan Databases	**Sample size:** 135,678<br><br>**Participants:** mother-child pairs with and without postpartum depression (PND) exposure<br><br>**Setting:** USA healthcare setting.<br><br>**Dates of data collection:** 2010 to 2016	**Primary Findings:**<br>• 33,314 mother-child pairs with PND exposure were propensity score matched to 102,364 mother-child pairs without PND exposure.<br>• During the 24-month follow-up period, HRU across most service categories was significantly higher among children in the PND exposure cohort than non-PND exposure cohort.<br>• Among outpatient services, the percentages of children with a physician specialist service (68% versus 64%), early-intervention screening (40% versus	This cohort study assessed healthcare resource utilisation (HRU) and costs in children of mothers with and without PND

1 ▪▪ Prices have been inflated and converted to GBP [53].

			37%), and an emergency room visit (48% versus 42%) were greater in children of mothers with PND (all *p* < .001).<br>• Furthermore, children of mothers with PND incurred 12% higher total healthcare costs in the first 24 months of life compared to children of mothers without PND ($24,572 versus $21,946; *p* < .001).<br>• After excluding mothers with preterm delivery, the proportion of children with ER visits, physician specialist services, and outpatient pharmacy claims was significantly higher in the PND exposure cohort than non-PND exposure cohort (all *p* < .001).<br>**Additional Findings:**<br>The results of this analysis suggest that HRU and costs over the first 24 months of life in children of mothers with PND exceeded that of children of mothers without evidence of PND.	
Petrou et al (2002)<br><br>(Petrou et al., 2002)<br><br>**UK**	**Study Design**: Economic evaluation in which unit costs were applied to resource-use data collected alongside a longitudinal study of women at high risk of developing PND. Unit costs were applied to estimates of health and social care resource use made by 206 women recruited from antenatal clinics and their infants. Net costs per mother-infant dyad over the first 18 months post-partum were estimated.<br><br>**Type of intervention [*exposure*]:** Preventative PND intervention.<br><br>**Data collection methods**: primiparous women attending antenatal clinics at 26–28 weeks of gestation were screened using a predictive index for PND. Women identified as being at high risk of developing PND were entered into an RCT of a preventive	**Sample size:** 206<br><br>**Participants:** Primiparous women at high risk of developing PND<br><br>**Setting:** antenatal clinics<br><br>**Dates of data collection:** May 1997 to April 1999	**Primary Findings:**<br>Mean mother-infant dyad costs were estimated at £2,419.00 for women with PND and £2026.90 for women without PND, a mean cost difference of £392.10 (P=0.17). The mean cost differences between women with and without PND reached statistical significance for community care services (P=0.01), but not for other categories of service. Economic costs were higher for women with extended experiences of the condition.	Aimed to estimate the economic costs of PND in a geographically defined cohort of women at high risk of developing the condition.

For peer review only - http://bmjopen.bmj.com/site/about/guidelines.xhtml

	intervention for PND delivered by trained health visitors. Economic data of women in the trial and in the observational study were pooled. An independent researcher assessed the mental state of all women at 8 weeks, 18 weeks, 12 months, and 18 months post-partum using the Structured Clinical Interview for DSM–III–R diagnoses (SCID–II).			
Petrou et al (2006)<br><br>(Petrou et al., 2006)<br><br>**UK**	**Study Design** A prospective economic evaluation was conducted alongside a pragmatic RCT<br><br>**Type of intervention [*exposure*]:** psychosocial and psychological interventions including counselling for the prevention of PND.<br><br>**Data collection methods:** Data on health and social care use by women and their infants up to 18 months postpartum were collected, using a combination of prospective diaries and face-to-face interviews	**Sample size:** 151 women<br><br>**Participants:** Women considered at high risk of developing PND were allocated randomly to the preventive intervention (*n* = 74) or to routine primary care (*n* = 77)<br><br>**Setting:** Health care setting.<br><br>**Dates of data collection:** c.2000	**Primary Findings:**<br>• Women in the preventive intervention group were depressed for an average of 2.21 months (9.57 weeks) during the study period, whereas women in the routine primary care group were depressed for an average of 2.70 months (11.71 weeks).<br>• The mean health and social care costs were estimated at £2,396.9 per mother–infant dyad in the preventive intervention group and £2,277.5 per mother–infant dyad in the routine primary care group, providing a mean cost difference of £119.5 (bootstrap 95 percent confidence interval [CI], −535.4, 784.9).<br>• At a willingness to pay threshold of £1,000 per month of PND avoided, the probability that the preventive intervention is cost-effective is .71 and the mean net benefit is £383.4 (bootstrap 95 percent CI, −£863.3–£1,581.5).<br><br>**Additional Findings:**<br>The preventive intervention is likely to be cost-effective even at relatively low willingness to pay thresholds for preventing 1 month of PND during the first 18 months postpartum. Given the negative impact of PND on later child development.	This cost-effectiveness analysis found that given the negative impact of PND on later child development, preventive interventions are likely to be cost-effective even at relatively low willingness to pay thresholds for preventing one month of PND during the first 18 months post-partum.
Roberts et al (2001)<br>[42]	**Study design:** Cross-sectional<br><br>**Data collection methods:** EPDS and the Health and Social Service Utilization Questionnaire (HSUQ)	**Sample size:** 1,250<br><br>**Participants:** mothers of infants.	**Primary findings:**<br>Costs were notably different for mothers with and without depression as determined by the EPDS (score of > 12). The total cost for health and social care $845 for mothers with	A cross-sectional study of 1250 mothers of infants in a Canadian setting used the EPDS to investigate the costs associated with perinatal depression. It was found that

For peer review only - http://bmjopen.bmj.com/site/about/guidelines.xhtml

**Canada**		**Setting:** Canadian healthcare setting<br><br>**Dates of data collection:** 1999	depression and their infant's vs $413 for those with lower scores. This was statistically significant difference at the (p < .01).<br>**Additional findings:**<br>Costs for social work visits were higher for mothers with depression and mothers with low incomes.<br>Total health and social care costs were double for mothers with family income below $20,000 ($788 v $399) and for mothers with clinical depression ($845 v $413). Nursing care costs were greater for mothers with high depression scores ($135 v $81).	costs were notably different for mothers with and without depression. The total cost for health and social care was $845 for mothers with depression and their infant's vs $413 for those with lower depression scores. This was statistically significant different at p < .01.
Stevenson et al (2010) (Stevenson et al., 2010)<br><br>UK	**Study Design:** cost-effectiveness analysis to assess group-CBT (gCBT) in comparison with routine primary care for women with PND in the UK.<br><br>**Type of intervention [*exposure*]:** Group-CBT<br><br>**Data collection methods:** SR	**Sample size:** 401<br><br>**Participants:** Data were analysed from 401 women with an EPDS score of 12 or greater at 6 weeks after childbirth, which had completed both the EPDS and the SF-6D questionnaire at both 6 weeks and 6 months<br><br>**Setting:** Postnatal healthcare setting in the UK<br><br>**Dates of data collection:** Pre-July 2009 (when PONDER study was published**).**	**Primary Findings:**<br>The mean cost per QALY from the stochastic analysis was estimated to be £36,062; however, there was considerable uncertainty around this value. The EVPI was estimated to be greater than £64 million; the key uncertainties were in the cost per woman of providing treatment and in the statistical relationship between changes in EPDS values and changes in SF-6D values. The expected value of perfect partial information for both of these parameters was more than £25 million.<br><br>**Additional Findings:**<br>The use of gCBT does not appear to be cost-effective; however, this decision is uncertain. The value of information analyses conducted indicates that further research to provide robust information on key parameters is needed and appears justified in cost-effective terms.	This economic evaluation found that gCBT does not appear to be cost-effective due to the lack of literature providing robust information. Only one study, an RCT, was deemed applicable to the decision problem.
Wilkinson et al (2017)<br><br>(Wilkinson et al., 2017)<br><br>**USA**	**Study Design:** Modelling study<br><br>**Type of intervention [*exposure*]:** N/A<br><br>**Data collection methods**: Hypothetical cohort	**Sample size:** 1,000<br><br>**Participants:** follows a hypothetical cohort of 1000 pregnant women experiencing one live birth over a 2-year time horizon.	**Primary Findings:**<br>• Screening for and treating postpartum depression and psychosis produced 29 more healthy women at a cost of $943 per woman.<br>• The incremental cost-effectiveness ratios of the intervention branch compared to usual care were $13,857 per QALY	This economic modelling study modelled the cost-effectiveness of physicians screening for and treating postpartum depression and psychosis in partnership with a psychiatrist.

For peer review only - http://bmjopen.bmj.com/site/about/guidelines.xhtml

		**Setting:** USA healthcare setting.<br><br>**Dates of data collection:** data were obtained from literature published between 1995 and 2015.	gained (below the commonly accepted willingness to pay threshold of \$50,000/QALY gained) and \$10,182 per remission achieved.<br>• These results were robust in both the deterministic and probabilistic sensitivity analyses of input parameters.<br><br>**Additional Findings:**<br>Screening for and treating postpartum depression is a cost-effective intervention and should be considered as part of usual postnatal care, which aligns with the recently proposed recommendations from the U.S. Preventive Services Task Force.	

For peer review only

Data extraction table for studies including maternal health and well-being

Citation (Country)	Study Details	Participants and setting	Key findings	Observations
Chojenta et al (2019)<br><br>(Chojenta et al., 2019)<br><br>Australia	**Study design:** Cross-sectional<br><br>**Data collection methods:** Health economics modelling study.<br><br>Data were taken from the Australian Longitudinal Study on Women's Health (ALSWH), an ongoing population-based study of health and well-being.	**Sample size:12,689**<br><br>**Participants:** Three cohorts of women born 1973–78, 1946–1951 and 1921–1926, with a fourth cohort born in 1989–1995 added in 2012.<br><br>**Setting:** Australian healthcare setting.<br><br>**Dates of data collection:** 1921 to 1995	**Primary findings:**<br>The healthcare costs for postnatal women who had poor mental health prior to birth was $1,792 (AUSD). This is on average 11% more than for mothers with no previous history of poor mental health.	This modelling study from Australia, utilising cohort data from 1921 to 1995 found that the healthcare costs for postnatal women who had poor mental health prior to birth was $1,792 (AUSD). This is on average 11% more than for mothers with no previous history of poor mental health.
Morrell et al (2000)<br><br>[34]<br><br>**UK**	**Study Design:** RCT<br><br>**Type of intervention [*exposure*]:**<br>Up to 10 home visits in the first postnatal month of up to three hours duration by a community postnatal support worker.<br><br>Impact of community postnatal support worker in addition to usual community midwife care on rest and recovery, health status, satisfaction with services and NHS Resource use and costs.<br><br>**Data collection methods:** Postal questionnaires (including SF36 and EPDS).	**Sample size:** 623<br><br>**Participants:** Postnatal women delivering at a university hospital<br><br>**Setting:** Home and community<br><br>**Dates of data collection:** Recruitment on labour wards from October 1996 to November 1997	**Primary Findings:** 551 completed 6 weeks questionnaire, 493 at 6 months. No evidence of use of fewer NHS services by women using the support worker versus controls at 6 weeks or 6 months.<br>Additional costs per woman at 6 weeks of £179.58 mostly due to support worker training ($p<0.001$).<br><br>**Additional Findings:** No diff primary outcome at 6 weeks but $p<0.05$ for physical and social functioning and p=005 EPDS for controls.<br>No difference in SF36 health status scores, EPDS scale or Duke Functional Social Support scale, rate of breastfeeding).	This study found that there were no savings to the NHS over six months after the introduction of a community support worker service and no improvement to the health status among the women in the intervention group, which was measured by an SF-36 questionnaire. At six weeks, the mean total NHS costs were £975▯ for the intervention group and £700 for the control group. At six months, the figures were £1,250 and £980, respectively.
Ride (2018)	**Study Design:** Modelling study (health economics)	**Date of model:** 2018<br><br>The models were developed using TreeAge Pro 2015 software (TreeAge Software, Inc.,	**Primary Findings:** The results suggest that broader boundaries, particularly extension of the time horizon, could make substantial differences to	By ignoring broader sets of costs and outcomes, resources in postnatal mental health may be misallocated, and as a result, some women may not benefit as

(Ride, 2018)<br><br>**UK**	**Data collection methods:** Decision analytic modelling	Williamstown, MA, USA). The population of interest was postnatal women and their children in the United Kingdom, because much of the data came from that setting; this gave an explicit societal threshold of £20,000 to £30,000 per QALY for cost-effectiveness analysis in health care. A health sector perspective was taken, except for the children's model, which expanded to a public sector perspective to accommodate educational costs. A discount rate of 3.5% was applied to costs and QALYs, with discounting applied back to the child's birth. All costs were converted to 2014 pounds sterling.	estimated cost-effectiveness. Inclusion of family effects without extension of the time horizon had little impact, but where a longer time horizon was used, family effects could make a significant difference to the conclusions drawn from cost-effectiveness analysis<br><br>**Additional Findings:** The authors note that it is important not only to consider caregiving but also family health effects in the outcomes of maternal health studies.	much from interventions that might be cost-effective given a broader time-horizon.

For peer review only

PRISMA 2020 Checklist

Section and Topic	Item #	Checklist item	Location where item is reported
**TITLE**			
Title	1	Identify the report as a systematic review.	Page 2
**ABSTRACT**			
Abstract	2	See the PRISMA 2020 for Abstracts checklist.	Figure 1
**INTRODUCTION**			
Rationale	3	Describe the rationale for the review in the context of existing knowledge.	Page 4
Objectives	4	Provide an explicit statement of the objective(s) or question(s) the review addresses.	Page 4
**METHODS**			
Eligibility criteria	5	Specify the inclusion and exclusion criteria for the review and how studies were grouped for the syntheses.	Page 5
Information sources	6	Specify all databases, registers, websites, organisations, reference lists and other sources searched or consulted to identify studies. Specify the date when each source was last searched or consulted.	Page 5
Search strategy	7	Present the full search strategies for all databases, registers and websites, including any filters and limits used.	Supplementary Material
Selection process	8	Specify the methods used to decide whether a study met the inclusion criteria of the review, including how many reviewers screened each record and each report retrieved, whether they worked independently, and if applicable, details of automation tools used in the process.	Page 6
Data collection process	9	Specify the methods used to collect data from reports, including how many reviewers collected data from each report, whether they worked independently, any processes for obtaining or confirming data from study investigators, and if applicable, details of automation tools used in the process.	Page 6
Data items	10a	List and define all outcomes for which data were sought. Specify whether all results that were compatible with each outcome domain in each study were sought (e.g. for all measures, time points, analyses), and if not, the methods used to decide which results to collect.	Page 6
	10b	List and define all other variables for which data were sought (e.g. participant and intervention characteristics, funding sources). Describe any assumptions made about any missing or unclear information.	Page 6
Study risk of bias assessment	11	Specify the methods used to assess risk of bias in the included studies, including details of the tool(s) used, how many reviewers assessed each study and whether they worked independently, and if applicable, details of automation tools used in the process.	Supplementary Material
Effect measures	12	Specify for each outcome the effect measure(s) (e.g. risk ratio, mean difference) used in the synthesis or presentation of results.	Page 8
Synthesis methods	13a	Describe the processes used to decide which studies were eligible for each synthesis (e.g. tabulating the study intervention characteristics and comparing against the planned groups for each synthesis (item #5)).	Page 6
	13b	Describe any methods required to prepare the data for presentation or synthesis, such as handling of missing summary statistics, or data conversions.	Page 6
	13c	Describe any methods used to tabulate or visually display results of individual studies and syntheses.	From Page 9
	13d	Describe any methods used to synthesize results and provide a rationale for the choice(s). If meta-analysis was performed, describe the model(s), method(s) to identify the presence and extent of statistical heterogeneity, and software package(s) used.	Page 8
	13e	Describe any methods used to explore possible causes of heterogeneity among study results (e.g. subgroup analysis, meta-regression).	N/A
	13f	Describe any sensitivity analyses conducted to assess robustness of the synthesized results.	N/A
Reporting bias assessment	14	Describe any methods used to assess risk of bias due to missing results in a synthesis (arising from reporting biases).	Supplementary Material
Certainty assessment	15	Describe any methods used to assess certainty (or confidence) in the body of evidence for an outcome.	

For peer review only

PRISMA 2020 Checklist

Section and Topic	Item #	Checklist item	Location where item is reported
assessment			
**RESULTS**			
Study selection	16a	Describe the results of the search and selection process, from the number of records identified in the search to the number of studies included in the review, ideally using a flow diagram.	Page 8
	16b	Cite studies that might appear to meet the inclusion criteria, but which were excluded, and explain why they were excluded.	Page 8
Study characteristics	17	Cite each included study and present its characteristics.	Page 8
Risk of bias in studies	18	Present assessments of risk of bias for each included study.	Page 8
Results of individual studies	19	For all outcomes, present, for each study: (a) summary statistics for each group (where appropriate) and (b) an effect estimate and its precision (e.g. confidence/credible interval), ideally using structured tables or plots.	Page 8
Results of syntheses	20a	For each synthesis, briefly summarise the characteristics and risk of bias among contributing studies.	Page 8
	20b	Present results of all statistical syntheses conducted. If meta-analysis was done, present for each the summary estimate and its precision (e.g. confidence/credible interval) and measures of statistical heterogeneity. If comparing groups, describe the direction of the effect.	Page 8
	20c	Present results of all investigations of possible causes of heterogeneity among study results.	Page 8
	20d	Present results of all sensitivity analyses conducted to assess the robustness of the synthesized results.	N/A
Reporting biases	21	Present assessments of risk of bias due to missing results (arising from reporting biases) for each synthesis assessed.	Supplementary Material
Certainty of evidence	22	Present assessments of certainty (or confidence) in the body of evidence for each outcome assessed.	Supplementary Material
**DISCUSSION**			
Discussion	23a	Provide a general interpretation of the results in the context of other evidence.	Page 25
	23b	Discuss any limitations of the evidence included in the review.	Page 2
	23c	Discuss any limitations of the review processes used.	N/A
	23d	Discuss implications of the results for practice, policy, and future research.	Page 26
**OTHER INFORMATION**			
Registration and protocol	24a	Provide registration information for the review, including register name and registration number, or state that the review was not registered.	Page 5
	24b	Indicate where the review protocol can be accessed, or state that a protocol was not prepared.	Page 5
	24c	Describe and explain any amendments to information provided at registration or in the protocol.	N/A
Support	25	Describe sources of financial or non-financial support for the review, and the role of the funders or sponsors in the review.	Page 27
Competing interests	26	Declare any competing interests of review authors.	Page 27
Availability of data, code and other materials	27	Report which of the following are publicly available and where they can be found: template data collection forms; data extracted from included studies; data used for all analyses; analytic code; any other materials used in the review.	Page 9 onwards

For peer review only

PRISMA 2020 Checklist

From: Page MJ, McKenzie JE, Bossuyt PM, Boutron I, Hoffmann TC, Mulrow CD, et al. The PRISMA 2020 statement: an updated guideline for reporting systematic reviews. BMJ 2021;372:n71. doi: 10.1136/bmj.n71
For more information, visit: http://www.prisma-statement.org/

For peer review only
